# Synthesis and In Vitro Biological Evaluation of *p*-Carborane-Based Di-*tert*-butylphenol Analogs

**DOI:** 10.3390/molecules28114547

**Published:** 2023-06-04

**Authors:** Sebastian Braun, Sanja Jelača, Markus Laube, Sven George, Bettina Hofmann, Peter Lönnecke, Dieter Steinhilber, Jens Pietzsch, Sanja Mijatović, Danijela Maksimović-Ivanić, Evamarie Hey-Hawkins

**Affiliations:** 1 Institut für Anorganische Chemie, Universität Leipzig, Johannisallee 29, 04103 Leipzig, Germany; braun1993@gmx.net (S.B.); loenneck@rz.uni-leipzig.de (P.L.); 2 Department of Immunology, Institute for Biological Research “Siniša Stanković”, National Institute of Republic of Serbia, University of Belgrade, Bul. Despota Stefana 142, 11060 Belgrade, Serbia; sanja.jelaca@ibiss.bg.ac.rs (S.J.); sanjamama@ibiss.bg.ac.rs (S.M.); nelamax@ibiss.bg.ac.rs (D.M.-I.); 3 Department of Radiopharmaceutical and Chemical Biology, Institute of Radiopharmaceutical Cancer Research, Helmholtz-Zentrum Dresden-Rossendorf, Bautzner Landstrasse 400, 01328 Dresden, Germany; m.laube@hzdr.de (M.L.); j.pietzsch@hzdr.de (J.P.); 4 Institute of Pharmaceutical Chemistry, University of Frankfurt, Max-von-Laue-Straße 9, 60438 Frankfurt, Germany; s.george@em.uni-frankfurt.de (S.G.); hofmann@pharmchem.uni-frankfurt.de (B.H.); steinhilber@em.uni-frankfurt.de (D.S.); 5 Faculty of Chemistry and Food Chemistry, Technische Universität Dresden, School of Science, Mommsenstrasse 4, 01062 Dresden, Germany

**Keywords:** carboranes, inflammation, cyclooxygenases, lipoxygenases, cancer

## Abstract

Targeting inflammatory mediators and related signaling pathways may offer a rational strategy for the treatment of cancer. The incorporation of metabolically stable, sterically demanding, and hydrophobic carboranes in dual cycloxygenase-2 (COX-2)/5-lipoxygenase (5-LO) inhibitors that are key enzymes in the biosynthesis of eicosanoids is a promising approach. The di-*tert*-butylphenol derivatives **R-830**, **S-2474**, **KME-4**, and **E-5110** represent potent dual COX-2/5-LO inhibitors. The incorporation of *p*-carborane and further substitution of the *p*-position resulted in four carborane-based di-*tert*-butylphenol analogs that showed no or weak COX inhibition but high 5-LO inhibitory activities in vitro. Cell viability studies on five human cancer cell lines revealed that the *p*-carborane analogs **R-830-Cb**, **S-2474-Cb**, **KME-4-Cb**, and **E-5110-Cb** exhibited lower anticancer activity compared to the related di-*tert*-butylphenols. Interestingly, **R-830-Cb** did not affect the viability of primary cells and suppressed HCT116 cell proliferation more potently than its carbon-based **R-830** counterpart. Considering all the advantages of boron cluster incorporation for enhancement of drug biostability, selectivity, and availability of drugs, **R-830-Cb** can be tested in further mechanistic and in vivo studies.

## 1. Introduction

Besides genetic mutations, epigenetic changes, diet, and lifestyle, chronic inflammatory processes are a significant factor in carcinogenesis [1,2]. The process of cancer formation is a stepwise progress which can be split into three phases, namely initiation, promotion, and progression. Cancer progression relies on the control of tumor microenvironment which is essential to provoke further tumor invasiveness and metastasis [3,4,5]. Hence, there is need for a balance between immunosuppressive and immunostimulatory mediators in the tumor- and inflammatory microenvironment [2,6,7,8]. Inflammatory mediators, such as cytokines, chemokines, and eicosanoids, can stimulate tumor cell proliferation and cancer progression by disrupting homeostatic mechanisms [3,9]. Eicosanoids, in particular prostaglandins (PGs) and leukotrienes (LTs), are hormone-like lipids that play a pivotal part in mediating the crosstalk between cells in the tumor microenvironment [2]. Targeting these inflammatory mediators by interfering with related signaling pathways may offer a rational strategy for the treatment of cancer. Pro-inflammatory PGs and LTs are mainly formed from arachidonic acid (AA) via cyclooxygenase-2 (COX-2) and 5-lipoxygenase (5-LO) signaling pathways [10,11,12]. Cyclooxygenases (COX) catalyze the rate-determining step in the biosynthesis of PGs [13,14,15]. The constitutively expressed COX-1 is involved in physiological “housekeeping” functions, such as the homeostasis of the gastrointestinal (GI) tract [16,17]. COX-2 is highly inducible by pro-inflammatory stimuli through macrophage activation or tumor promoters [12,18]. It can be found in macrophages, leukocytes, and fibroblasts, and COX-2 dependent production of pro-inflammatory PGs is upregulated during inflammatory diseases, such as rheumatoid arthritis, cardiovascular diseases, and asthma [10,12,15,19]. Furthermore, COX-2, in particular the downstream product prostaglandin E_2_ (PGE_2_), is able to promote carcinogenesis and progression in different cancer types, such as skin, breast, colon, and lung cancer, that are associated with poor prognosis [2,15,20,21,22]. As COX-1 and COX-2 share nearly homologous amino acid sequence identity, selective COX-2 inhibition remains challenging [17,19]. COX-2 is physiologically repressed by the active form of vitamin D and glucocorticoids [23,24]. Artificial COX inhibitors are categorized into two groups, namely non-steroidal anti-inflammatory drugs (NSAIDs) and COXIBs. As a result, of two crucial changes in the amino acid sequence, the COX-2 active site is ca. 17% larger and has a slightly different form than that of COX-1 [25]. Thus, size enlargement of inhibitors has been shown to enable COX-2 selective inhibition [19,20]. While non-selective COX inhibition by NSAIDs leads to the reduction of gastroprotective PGs, COX-2 selective inhibition suffers from adverse cardiovascular effects caused by dysbalanced prostaglandin I_2_ (PGI_2_) and thromboxane A_2_ (TXA_2_) levels [2,25,26,27,28].

5-LO is a non-heme iron-containing oxidoreductase that belongs to a heterogeneous family of lipid peroxidizing enzymes and catalyzes the conversion of AA into LTs with the help of 5-lipoxygenase-activating protein (FLAP) [10,29,30,31]. These important lipid mediators are involved in host defense, inflammatory processes, and cellular signaling [2,32]. 5-LO is highly inducible and can be stimulated by growth factors and pro-inflammatory stimuli [20,28,33]. Indeed, 5-LO upregulation and LTs overproduction are related to hypersensitivity reactions, inflammatory diseases, and allergic disorders, such as arthritis or psoriasis. It has also been observed in different types of epithelial cancers, such as lung, colon, and prostate cancer [9,12,33,34,35,36]. 5-LO can be downregulated by four different types of inhibitors, namely redox (non-competitive), iron-chelating, non-redox (competitive), and allosteric inhibitors [37,38,39]. 5-LO inhibitors, in general, suffer from having a short half-life and exhibiting hepatic toxicity [10,32].

PGs and LTs have complementary effects on the pathogenesis of cancer and tumor progression, and thus dual COX-2/5-LO inhibition may represent a promising strategy to treat cancer and to prevent the pathogenesis of cancer [33,34,40]. Dual COX-2/5-LO inhibitors block the release of both PGs and LTs from the corresponding COX-2 and 5-LO signaling pathways [10,17]. Dual COX-2/5-LO inhibition may prevent the upregulation of the respective opposed signaling pathway by blocking both targets. Consequently, balanced COX-2/5-LO inhibition may lower the risk for the appearance of severe adverse effects, such as GI injury and hypersensitive reactions [21,38,41,42,43,44].

**Figure 1:**
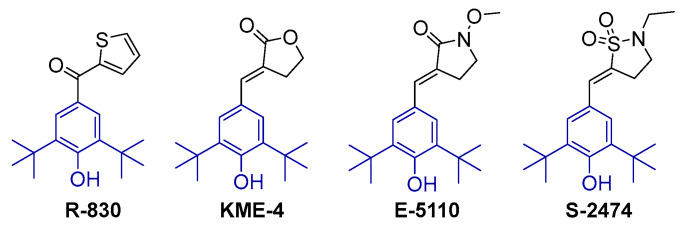
Chemical structures of selected di-*tert*-butylphenols showing the conserved 2,6-di-*tert*-butylphenol motif (blue).

Di-*tert*-butylphenols represent the predominant class of dual COX-2/5-LO inhibitors and share a conserved 2,6-di-*tert*-butylphenol motif substituted at the *para (p)*-position (Figure 1). Di-*tert*-butylphenols show a superior therapeutic index, i.e., the ratio of anti-inflammatory effect to GI safety profile, compared to that of classical NSAIDs [10,33,34]. Several di-*tert*-butylphenols have been synthesized and pharmacologically evaluated. **R-830** (3,5-di-*tert*-butyl-4-(2′-thienoyl)-phenol) produces anti-edemic, analgesic, antipyretic, and anti-erythemic effects in several animal models [45]. It further reduces PGE_2_ production by stimulated rat synovial cells in the nanomolar range and significantly inhibited leukotriene B_4_ (LTB_4_) production by human neutrophils at micromolar concentrations [45,46]. The dual COX-2/5-LO inhibitor **KME-4** (*α*-3,5-di-*tert*-butyl-4-hydroxybenzylidene)-*γ*-butyrolactone) inhibits both guinea pig peritoneal polymorphonuclear leukocytes (PMNL) and rabbit platelet COX at low micromolar concentrations [47,48]. Moreover, in vivo experiments revealed that **KME-4** possess anti-arthritic activity in a dose-dependent manner, while showing lower ulcerogenic potential compared to other NSAIDs, such as indomethacin or naproxen [49,50].

The pyrrolidone derivative **E-5110** (*N*-methoxy-3-(3,5-di-*tert-*butyl-4-hydroxybenzylidene)-2-pyrrolidone) inhibits both PGE_2_ production by cultured rat synovial cells at nanomolar concentrations and LTB_4_ generation by human leukocytes stimulated in the micromolar range [51,52,53,54]. Further in vivo studies revealed that **E-5110** exhibits analgesic activity similar to indomethacin with lower ulcerogenic effect on rat gastric mucosa than indomethacin and piroxicam [54,55]. Both in vitro and in vivo toxicokinetic studies on the metabolism of **E-5110** demonstrated induction of cytochrome P_450_ 3A (CYP3A) activity, which is therefore expected to mainly contribute to its metabolism as well as its bioavailability [56]. **S-2474** ((*E*)-5-(3,5-di-*tert*-butyl-4-hydroxybenzylidene)-2-ethylisothiazo-lidine-1,1-dioxide) is a selective inhibitor of COX-2 and 5-lipoxygenase in vitro with half-maximal inhibitory concentration (IC_50_) values in the low micromolar and nanomolar range, respectively [57]. Furthermore, it shows in vivo anti-inflammatory activity in rats with low ulcerogenic activity due to its COX-2 selectivity [58].

A promising approach to increase metabolic stability of carbon-based drugs is the incorporation of hydrophobic boron clusters, such as the 12-vertex dicarba-*closo*-dodecaborane (carborane), as bioisosters of substituted or unsubstituted phenyl rings [59,60,61]. Carboranes are icosahedral boron clusters in which at least one BH^−^ vertex in (B_12_H_12_)^2−^ is replaced by an isolobal CH group [61,62,63]. As their distorted icosahedral shape reflects the shape of a rotating phenyl ring, they can be considered benzene analogs [63,64,65,66]. In analogy to the nomenclature of the substitution of a benzene ring, three different regioisomers, namely 1,2-(*ortho*), 1,7-(*meta*), or 1,12-carborane (*para*), are reported [64,67,68]. Due to a slightly larger van-der-Waals diameter compared to a phenyl ring (carborane: 5.25 Å, phenyl ring: 4.72 Å) [59], carboranes are frequently used as phenyl mimetics for biologically active compounds [61,62,65,69]. Multiple non-covalent interactions, like dihydrogen bond formation, may increase the affinity to biological targets, including enzymes or receptor proteins [61,62,65]. Due to their remarkable properties, carboranes offer a wide range of possibilities for drug design in medicinal chemistry [61,70].

Our group has intensively investigated the introduction of carborane moieties into NSAIDs, COXIBs, and 5-LO inhibitors [71,72,73,74,75,76,77]. We have recently reported the first carborane-based dual COX-2/5-LO inhibitors that are derived from RWJ-63556 showing excellent inhibitory potential toward COX-2 and 5-LO, accompanied by high anticancer activity on the A375 melanoma cell line [78].

Herein, we report the synthesis of *p*-carborane-containing analogs of the di-*tert*-butylphenol derivatives **R-830**, **KME-4**, **E-5110**, and **S-2474**, their inhibitory potential toward COX-1, COX-2, and 5-LO, as well as their cytotoxicity on five human cancer cell lines.

## 2. Results and Discussion

### 2.1. Design and Synthesis of Di-tert-butylphenol Analogs

**Scheme 1:**
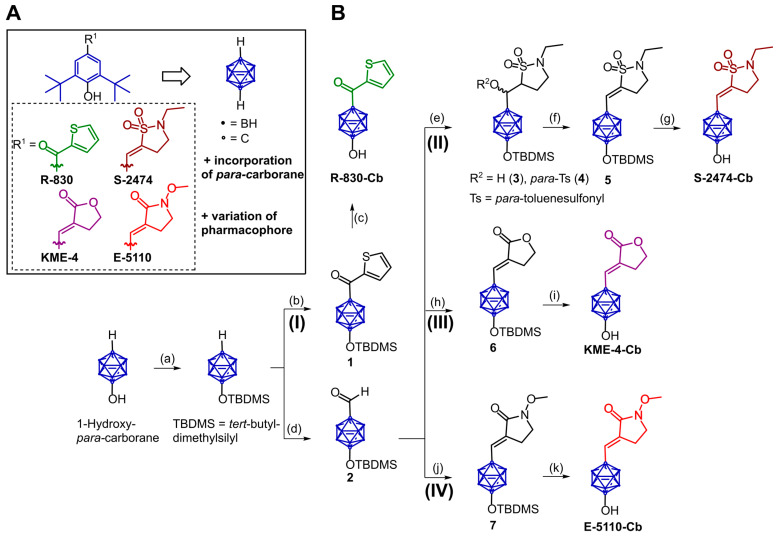
(**A**) Synthesis of carborane-based di-*tert*-butylphenol analogs **R-830-Cb**, **S-2474-Cb**, **KME-4-Cb**, and **E-5110-Cb**. (**B**) Reagents and conditions: (**I**) Synthetic entry towards *p*-substituted di-*tert*-butylphenol analog **R-830-Cb**. (a) 1. TBDMSCl, NEt_3_ and 4-dimethylaminopyridine (DMAP), DCM, 0 °C → RT, 48 h; 2. HCl_(aq)_, RT (97% yield); (b) 1. *n*BuLi, Et_2_O, 0 °C → RT, 2 h; 2. 2-thiophenecarbonyl chloride, 0 °C → RT, 24 h; 3. HCl_(aq)_, RT (64% yield); (c) 1. TBAF, THF, 0 °C, 20–30 min; 2. H_2_O, RT (90% yield); Key intermediate: (d) 1. *n*BuLi, Et_2_O, 0 °C → RT, 2 h; 2. methyl formate, 0 °C → RT, 24 h; 3. HCl_(aq)_, RT (86% yield); (**II**) Synthetic entry towards *p*-substituted di-*tert*-butylphenol analog **S-2474-Cb**. (e) 1. 2-ethylisothiazolidine-1,1-dioxide, THF, −78 °C, 30 min; 2. *n*BuLi, THF, −78 °C, 1.5 h → RT; 3. HCl_(aq)_, RT (53% yield); 4. NaH, THF, RT, 2 h; 5. *p*-toluenesulfonyl chloride, 0 °C → RT, 24 h; 6. HCl_(aq)_, RT (87% yield); (f) 1. NaH, THF, RT, 2 h; 2. H_2_O, RT (64% yield); (g) 1. TBAF, THF, 0 °C, 20–30 min; 2. H_2_O, RT (96% yield); (**III**) Synthetic entry towards *p*-substituted di-*tert*-butylphenol analog **KME-4-Cb**; (h) 1. 3-(triphenylphosphoranylidene)-*γ*-butyrolactone, THF/DMF, 120 °C, 48 h; 2. H_2_O, RT (84% yield); (i) 1. TBAF, THF, 0 °C, 25 min; 2. H_2_O, RT (99% yield); (**IV**) Synthetic entry towards *p*-substituted di-*tert*-butylphenol analog **E-5110-Cb**; (j) 1. (1-methoxy-2-oxo-3-pyrrolidinyl)triphenyl-phosphonium bromide, NEt_3_, THF, RT, 45 min; 2. DMF, 75 °C, 24 h; 3. H_2_O, RT (50% yield); (k) 1. TBAF, THF, 0 °C, 30 min; 2. H_2_O, RT (93% yield).

We recently reported the preparation and biological evaluation of eight meta (*m*)- and *p*-carborane analogs of the di-*tert*-butylphenol derivative tebufelone [79]. There, the incorporation of carborane moieties led to loss of COX inhibition, but retained 5-LO inhibitory potential, and to an overall enhanced anticancer activity. Inspired by these results, in particular by the *p*-carborane-containing analogs, we have now extended the investigation on the bioisosteric replacement of the bulky di-*tert*-butylphenyl motif with a *p*-carborane moiety to the di-*tert*-butylphenols **R-830**, **KME-4**, **E-5110**, and **S-2474**. These compounds are potent dual COX-2/5-LO inhibitors sharing a conserved 2,6-di-*tert*-butylphenol motif, but they differ in the *p*-position bearing different pharmacophores, such as thiophene, *γ*-sulfonyl lactam, lactam, and *γ*-lactones (Scheme 1). Katsumi and co-workers demonstrated that the structural combination of at least one *tert*-butyl group at the *ortho*-position and an oxygen atom at the *p*-position is essential for anti-inflammatory activity [51,53]. Accordingly, four *p*-carborane-containing di-*tert*-butylphenol analogs were synthesized by replacing the 2,6-di-*tert*-butylphenol motif with a 1-hydroxy-*p*-carborane moiety while keeping the pharmacophoric group (Scheme 1). 1-Hydroxy-*p*-carborane was prepared according to a published procedure [80] and converted quantitatively into the corresponding *tert*-butyldimethylsilyl (TBDMS) ether by a conventional silylation reaction with excess of *tert*-butyldimethylsilyl chloride (TBDMSCl) in the presence of triethylamine (NEt_3_) catalyzed by 4-dimethylaminopyridine (DMAP). The TBDMS-protected *p*-carborane-containing **R-830** analog **1** was obtained by deprotonation and lithiation of the TBDMS-protected hydroxy-*p*-carborane with *n*BuLi followed by reaction with excess of the corresponding 2-thiophenecarbonyl chloride in good yield (64%). **1** was subsequently deprotected with tetra-*n*-butylammonium fluoride (TBAF) to obtain the desired analog **R-830-Cb** in almost quantitative yield (90%, Scheme 1, (I)). The key intermediate 1-(*tert*-butyl-dimethylsiloxy)-12-formyl-1,12-dicarba-*closo*-dodecaborane(12) (*p*-carboranylaldehyde **2**) was obtained by deprotonation and lithiation of the TBDMS-protected 1-hydroxy-*p*-carborane with *n*BuLi followed by addition of excess methyl formate in almost quantitative yield (86%). The *p*-carborane analog **S-2474-Cb** was prepared in a four-step sequence starting from 2-ethylisothiazolidine-1,1-dioxide and *p*-carboranylaldehyde **2** (Scheme 1, (II)). Selective *α*-proton abstraction of the *γ*-sulfonyl lactam precursor by *n*BuLi under kinetic control followed by addition of the *p*-carboranylaldehyde **2** led to formation of the intermediate alcohol **3** in moderate yield (53%). Compound **3** was further converted to the corresponding tosylate **4** by deprotonation of the hydroxyl group with sodium hydride (NaH) and subsequent reaction with excess *p*-toluenesulfonyl chloride (87% yield) to introduce an excellent leaving group. Reaction with NaH, a strong non-nucleophilic base, gave the TBDMS-protected *p*-carborane analog of **S-2474 5** in good yield (64%). Deprotection of the alcohol **5** with TBAF gave the *p*-carborane analog **S-2474-Cb** in excellent yield (96%, Scheme 1). The *p*-carborane-containing analogs **KME-4-Cb** and **E-5110-Cb** were prepared in two steps starting from *p*-carboranylaldehyde **2** and the corresponding Wittig reagents 3-(triphenylphosphoranylidene)-*γ*-butyrolactone and (1-methoxy-2-oxo-3-pyrrolidinyl)triphenyl-phosphonium bromide, respectively (Scheme 1, (III) and (IV)). The Wittig reagents were prepared according to literature procedures und represent key intermediates in the synthesis of both target compounds **KME-4-Cb** [81] and **E-5110-Cb** [54]. The TBDMS-protected analogs **6** and **7** were obtained by reaction of the *p*-carboranylaldehyde **2** and the corresponding Wittig reagents with or without the presence of NEt_3_ in good to excellent yields (50–84%). Finally, the isolated TBDMS-protected compounds **6** and **7** were quantitatively converted to the final products **KME-4-Cb** (99%) and **E-5110-Cb** (93%) by TBAF-mediated deprotection of the hydroxyl groups.

**Figure 2:**
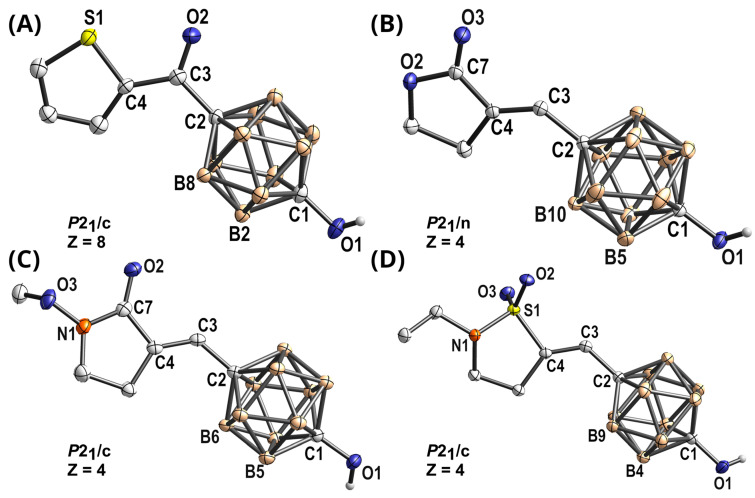
Molecular structure of compounds: (**A**) **R-830-Cb**, (**B**) **KME-4-Cb**, (**C**) **E-5110-Cb**, and (**D**) **S-2474-Cb**. Hydrogen atoms, except for O-H bonds, are omitted for clarity. Thermal ellipsoids are drawn at 50% probability level (beige = B, grey = C, blue = O, orange = N, yellow = S). Selected bond lengths, distances and bond angles are given in the Supplementary Materials, Tables S3–S6.

All compounds were fully characterized by NMR and IR spectroscopy, mass spectrometry and elemental analysis. Three-dimensional structures of intermediates **1**, **5**, **6**, and **7**, as well as of *p*-carborane-containing analogs **R-830-Cb**, **S-2474-Cb**, **KME-4-Cb**, and **E-5110-Cb**, were determined by single-crystal X-ray crystallography (Figure 2; for further details, see Supplementary Materials, Tables S3–S6).

Solubility and chemical stability in organic solvents, such as aqueous dimethyl sulfoxide (DMSO), are crucial for biological investigations. Therefore, the stability of the *p*-carborane-containing compounds **R-830-Cb**, **S-2474-Cb**, **KME-4-Cb**, and **E-5110-Cb** in aqueous DMSO-d_6_ at room temperature in air was studied by ^1^H- and ^11^B{^1^H}-NMR spectroscopy over four weeks and confirmed that all compounds are stable (see Supplementary Materials, Figure S24).

### 2.2. Determination of Lipophilicity (logD) by HPLC

The lipophilicity was determined as log*D*_7.4,HPLC_ value by a high-performance liquid chromatography (HPLC) method originally described by Donovan and Pescatore (Table 1) [82]. **R-830**, **KME-4**, **S-2474** and **E-5110** have log*D* values in the range of 3.35–3.91. Interestingly, the introduction of the lipophilic carborane cluster in **R-830-Cb**, **KME-4-Cb**, **S-2474-Cb**, and **E-5110-Cb** instead of the bulky but also lipophilic di-*tert-*butylphenyl motif resulted in a marked decrease in lipophilicity and log*D* values in the range of 1.50–2.37.

### 2.3. Evaluation of Inhibitory Potential toward COX

All compounds were tested in vitro at a concentration of 100 µM for their inhibitory potential toward ovine COX-1 and human recombinant COX-2 using the COX Fluorescent Inhibitor Screening Assay Kit (Cayman Chemical Company) employing the selective COX-2 inhibitor celecoxib as well as the COX-1 inhibitor SC-560 as references (Table 1). While all di-*tert-*butylphenol derivatives showed the expected inhibition of COX-2 and COX-1 as reported in the literature [46,47,48,49,57], no or only weak inhibition (<26%) of both isoforms was observed for the respective *p*-carborane analogs (Table 1).

This finding is in line with previous reports claiming that the presence of at least one *tert*-butyl and a hydroxyl group is essential for anti-inflammatory activity [51,53] which could not be compensated by the hydroxy-substituted *p*-carborane cluster.

### 2.4. Evaluation of Inhibitory Potential toward 5-LO

All compounds were tested for their 5-LO inhibitory activity in an intact cell assay (polymorphonuclear leukocytes, PMNL) by using the selective 5-LO inhibitor BWA4C as control (Table 2). The four carborane-based analogs inhibited 5-LO product formation, with IC_50_ values in the nanomolar range, comparable to those of the respective reference compounds. These results confirm that the substitution of the di-*tert*-butylphenyl motif by *p*-carborane is well tolerated and leads to strong inhibitors of 5-LO product formation.

### 2.5. In Vitro Determination of Cell Viability

Five human cell lines (A375 melanoma, A549 lung adenocarcinoma, HCT116 and HT29 colorectal carcinoma, MDA-MB-231 triple-negative breast adenocarcinoma) were used to study the antitumor effects of the di-*tert*-butylphenols and their respective carborane analogs. All selected cell lines were derived from inflammation-associated tumors [83,84]. Culturing cells in the presence of **R-830**, **KME-4**, **E-5110**, and **S-2474** resulted in a dose-dependent decrease in cell viability determined after 72 h (Table 3, Supplementary Materials, Figures S27 and S28).

Incorporation of a *p*-carborane moiety resulted in a significant decrease in cytotoxic potential for all tested compounds, except **R-830-Cb**, which showed antitumor potential in a similar micromolar range as the parent compound. In general, values obtained with the 3-(4,5-dimethylthiazol-2-yl)-2,5-diphenyltetrazolium bromide (MTT) assay were significantly lower than those obtained with the crystal violet (CV) assay, as expected, because dual COX-2/5-LO inhibitors affect the redox status of cells [85]. Since HCT116 cells were highly sensitive to the carborane analog **R-830-Cb** and the di-*tert*-butylphenols, the antitumor effects of the compounds might be independent of the inhibition of COX-2 and 5-LO [86,87]. Accordingly, **R-830-Cb** was selected for further in-detail investigation of the mechanism of action. Treatment of primary peritoneal exudate cells isolated from healthy mice with **R-830** or its carborane counterpart **R-830-Cb** showed that the latter one was completely non-toxic in the applied dose range, whereas **R-830** diminished the viability of primary cells at 100 µM (selectivity index (SI) ≈ 3, Supplementary Materials, Figure S29). These results suggest that the introduction of *p*-carborane preserves the cytotoxic potential for the malignant phenotype without affecting normal cells in the tested dose range.

**Figure 3:**
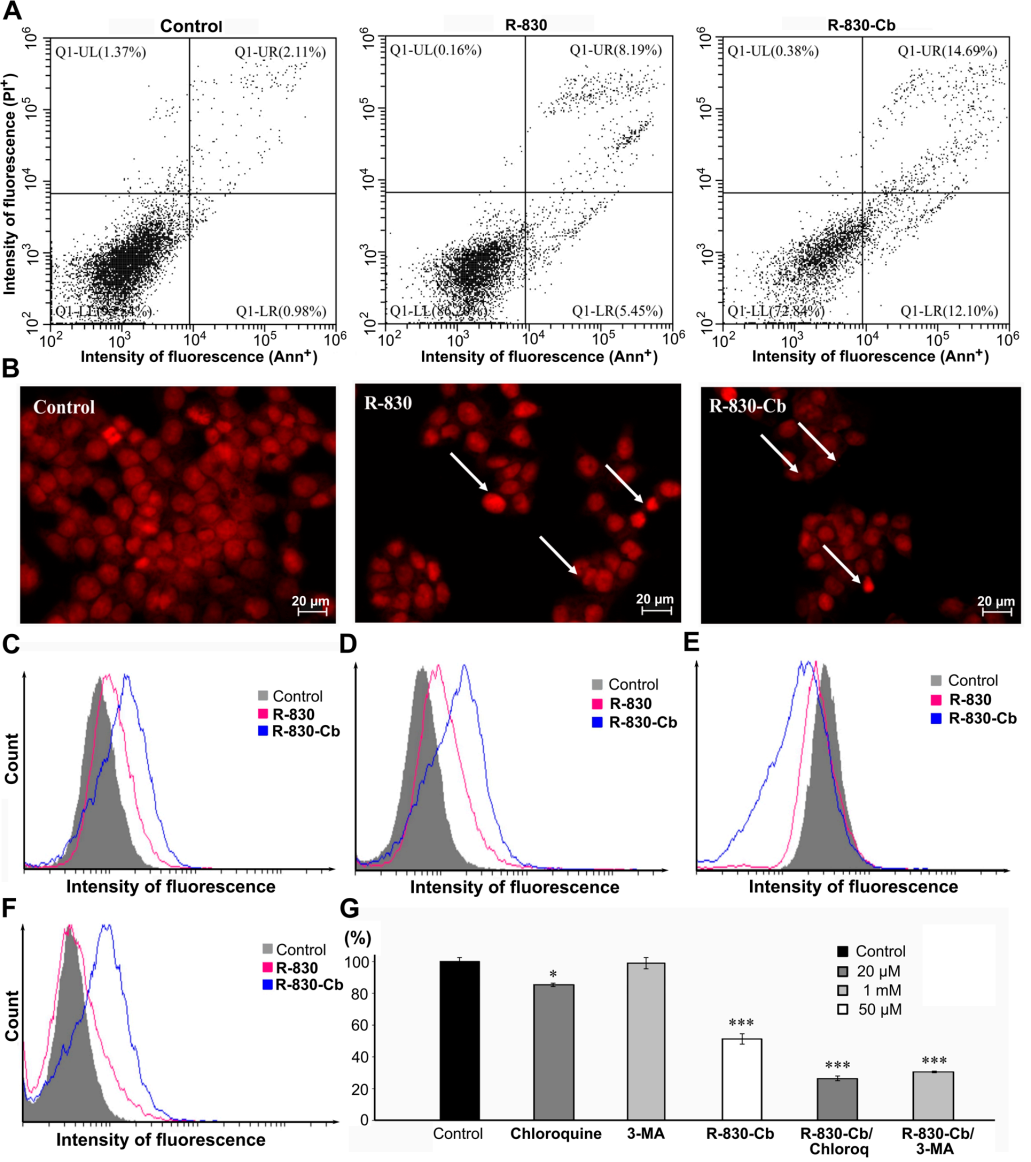
Mode of action of **R-830** and **R-830-Cb**. HCT116 cells were treated with an IC_50_ dose of **R-830** and **R-830-Cb** for 72 h and stained with (**A**) Annexin V (AnnV)/propidium iodide (PI), (**B**) PI (white arrows mark apoptotic nuclei), (**C**) Apostat, (**D**) Carboxyfluorescein diacetate succinimidyl ester (CFSE), (**E**) Dihydrorhodamine 123 (DHR 123), and (**F**) Acridine orange (AO). (**G**) HCT116 cells were co-treated with **R-830-Cb** and 3-methyladenine (3-MA) or chloroquine and viability was estimated by crystal violet (CV) assay. * p < 0.05; *** *p* < 0.001 in comparison to relevant control.

Analysis of apoptotic cell death by flow cytometry and fluorescent microscopy showed that the mechanism of antitumor action of **R-830** was preserved and even enhanced by the incorporation of *p*-carborane (Figure 3A,B). Treatment with **R-830-Cb** not only resulted in lower cell density, but also revealed the presence of numerous cells with abnormally shaped nuclei and condensed chromatin (Figure 3B).

Apostat staining confirmed that apoptosis was in correlation with the activation of caspases (Figure 3C). At the same time, and in parallel with the presence of apoptotic cells, cell division was more severely affected by **R-830-Cb** (Figure 3D). The decreased production of reactive oxygen species explains the observed effects of the applied treatments, namely inhibition of proliferation and induction of apoptosis (Figure 3E). It is widely accepted that drug-mediated hyperproduction of reactive oxygen species that are not buffered by cellular anti-oxidative protection is responsible for cell death induction [88]. However, the mechanisms of action of some therapeutics rely on the fact that tumor cells, especially colon carcinomas, produce a significantly higher amount of reactive oxygen species necessary for their metabolic and proliferative demands [89]. The described feature is acquired during disease progression, and it is tightly connected with abnormal blood supply and a hypoxic microenvironment [90]. Therefore, elimination of these molecules results in a rapid decrease in cell viability. Importantly, the intense autophagy detected by acridine orange (AO) supravital staining of cells exposed to **R-830-Cb**, revealed cytoprotective character for this compound, but not for **R-830** (Figure 3F,G).

Ultimately, simultaneous treatment of cells with **R-830-Cb** and specific autophagy inhibitors, chloroquine or 3-methyladenine (3-MA), showed that suppression of the autophagy process caused a drug-induced decrease in cell viability (Figure 3G). This means that the cytotoxic activity of the tested drug is enhanced by the inhibition of autophagy.

Considering the disadvantages of 2D cell cultures, since the cells are in a monolayer without all the components of the tumor microenvironment, the real improvement and potential of **R-830-Cb** should be discussed in a more complex experimental setting [91].

## 3. Experimental Section

### 3.1. Materials and Methods

All reactions were carried out under nitrogen using standard Schlenk techniques. All solvents were degassed, dried and purified with the solvent purification system SPS-800 by MBraun. Molecular sieves (3 and 4 Å) were activated at 300 °C in vacuum for a minimum of three hours for storage of dry solvents. Hexane has been used as a mixture of different isomers. For column chromatography, silica gel 60 with a particle size in the range of 0.035–0.070 mm (Acros) was used. A gradient of hexane and ethyl acetate (EtOAc) was usually used as the eluent system. Reactions were monitored with thin-layer chromatography using glass plates coated with silica gel (0.25 mm, silica gel 60, F_254_, Merck). Carborane-containing areas were stained and identified with a 5% solution of palladium(II) chloride in methanol. All NMR spectra were recorded on a Bruker Avance DRX 400 spectrometer at the following measurement frequencies at 26 °C: ^1^H-NMR 400.16 MHz, ^11^B-NMR 128.38 MHz, ^13^C-NMR 100.63 MHz. Tetramethylsilane (TMS) served as an internal standard for ^1^H-NMR spectra, and deuterated chloroform (CDCl_3_) (7.26 ppm) was used for referencing. Signal multiplicities were indicated by the following abbreviations and combinations: br = broad, s = singlet, d = doublet, t = triplet, q = quartet, dd = doublet of doublets, dt = doublet of triplets, td = triplet of doublets, ddd = doublet of doublets of doublets, dtd = doublet of triplets of doublets. The unit of the coupling constant *J* is given in hertz (Hz). The chemical shifts in the ^13^C{^1^H}-NMR spectra could be extracted from the spectra by ^1^H broadband decoupling in the proton channel. Residual signals of CDCl_3_, acetone-d_6_, deuterated dimethyl sulfoxide (DMSO-d_6_) at 77.2, 29.9, 39.5 ppm served as a reference. All ^11^B{^1^H} NMR spectra were also internally referenced to TMS with the aid of the Ξ scale [92]. The analysis of the data of the measurement was carried out using the software Mestrenova 14. ESI mass spectra were measured using a Bruker Daltonics Apex II FT-ICR spectrometer. All IR spectra were obtained using an ATR-IR spectrometer (Nicolet iS5, Thermo Fisher Scientific, Waltham, MA, USA) in the range of 400–4000 cm^−1^. Elemental analyses were carried out with a Heraeus Vario El analyser. The melting points were determined in glass capillaries using a Gallenkamp MPD350·BM2.5 apparatus and are uncorrected. 1,12-Dicarba-*closo*-dodecaborane(12) (Katchem, Prague, Czech Republic), 2-ethylisothiazolidine-1,1-dioxide (BLD Pharmatech Ltd., Shanghai, China), 2-thiophenecarbonyl chloride (Alfa Aesar, Ward Hill, MA, USA) are commercially available. All chemicals were used without further purification. 2,6-Bis(1,1-dimethylethyl)-4-[(*E*)-(2-ethyl-1,1-dioxido-5-isothiazolidinylidene)methyl]phenol (**S-2474**) [58], 3-([3,5-bis(1,1-dimethylethyl)-4-hydroxyphenyl]methylene)dihydro-2(3*H*)-furanone (**KME-4**) [53], 3-([3,5-bis(1,1-dimethylethyl)-4-hydroxyphenyl]methylene)-1-methoxy-2-pyrrolidinone (**E-5110**) [54], 2,6-di-*tert*-butyl-4-(2’-thenoyl)phenol (**R-830**) [93], (1-methoxy-2-oxo-3-pyrrolidinyl)triphenyl-phosphonium bromide [54], 1-(*tert*-butyl-dimethylsiloxy)-1,12-dicarba-*closo*-dodecaborane(12) [94], and 3-(triphenylphosphoranylidene)-*γ*-butyrolactone [81] were prepared according to the literature.

### 3.2. Synthetic Procedures

#### 3.2.1. 1-(*tert*-Butyl-dimethylsiloxy)-12-(thiophen-2′-carbonyl)-1,12-dicarba-*closo*-dodecaborane(12) (**1**)

1-(*tert*-Butyl-dimethylsiloxy)-1,12-dicarba-*closo*-dodecaborane(12) (1.00 eq., 0.83 g, 3.02 mmol) was dissolved in 25 mL dry diethyl ether. At 0 °C, *n*BuLi (1.05 eq., 1.43 M in hexane, 2.32 mL, 3.32 mmol) was added dropwise. Afterwards, the solution was stirred at room temperature for two hours. At 0 °C, a solution of 2-thiophenecarbonyl chloride in 10 mL dry diethyl ether (1.50 eq., 0.48 mL, 4.53 mmol) was added dropwise. The solution was stirred at room temperature for 24 h, then 20 mL of a 1 M HCl was added and the phases were separated. The aqueous phase was extracted three times with 25 mL diethyl ether, the combined organic phases were washed twice with 25 mL brine and then dried over magnesium sulfate. The solvent was removed under reduced pressure and the crude product was purified by column chromatography (SiO_2_, hexane → hexane/EtOAc, 30:1, *v*/*v*). Colorless solid, yield 64% (0.74 g, 1.92 mmol); m.p.: 102–103 °C; TLC (hexane/EtOAc, 30:1, *v*/*v*): *R*_f_ = 0.51. ^1^H-NMR (400.16 MHz, CDCl_3_): *δ* [ppm] = 0.10 (s, 6H, 8-C**H**_3_), 0.76 (s, 9H, 10-C**H**_3_), 1.60–3.52 (br m, 10H, B**H**), 7.06 (dd, ^3^*J*_HH_ = 5.0; 4.0 Hz, 2H, 2-C**H**), 7.64 (dd, ^3^*J*_HH_ = 5.0, 1.1 Hz, 1H, 1-C**H**), 7.83 (dd, ^3^*J*_HH_ = 4.0, 1.1 Hz, 1H, 3-C**H**); ^13^C{^1^H}-NMR (100.63 MHz, CDCl_3_): *δ* [ppm] =−4.18 (8-**C**H_3_), 17.8 (9-**C**_q_), 25.1 (10-**C**H_3_), 71.5 (6-**C**_q_), 113.4 (7-**C**_q_), 128.2 (2-**C**_q_), 135.8 (1-**C**_q_), 135.9 (3-**C**_q_), 140.6 (4-**C**_q_), 179.8 (5-**C**_q_); ^11^B{^1^H}-NMR (128.37 MHz, CDCl_3_): *δ* [ppm] = −15.4 (s, 5**B**), −12.8 (s, 5**B**); FTIR (ATR): $\tilde{\nu }$ [cm^−1^] = 3126 (m, *ν*_C–H_), 2959 (m, *ν*_C–H_), 2929 (m, *ν*_C–H_), 2857 (m, *ν*_C–H_), 2620 (s, *ν*_B–H_), 2599 (s, *ν*_B–H_), 1644 (s, *ν*_C=O_), 1515 (m, v_c=c_), 1470 (m, *δ*_C–H_), 1462 (m, *δ*_C–H_), 1409 (s, *δ*_C–H_), 1355 (m, *δ*_C–H_), 1247 (s, *ν*_C=S_), 1115 (s), 1064 (m), 1021 (s), 947 (s), 866 (w), 839 (m), 803 (m), 781 (s), 755 (m), 725 (s, *ν*_B-B_), 688 (s), 673 (s), 656 (s), 603 (m), 574 (m), 493 (w), 466 (m); HR-ESI-MS (negative mode, CH_3_CN/CH_2_Cl_2_): *m/z* [M–SiMe_2_*t*Bu]^−^, calcd. for C_7_H_13_B_10_O_2_S: 271.1572, found: 271.1579; elemental analysis: calcd. (%) for C_13_H_28_B_10_O_2_SSi: C 40.60, H 7.34, found: C 40.75, H 7.14.

#### 3.2.2. 1-Hydroxy-12-(thiophen-2′-carbonyl)-1,12-dicarba-*closo*-dodecaborane(12) (**R-830-Cb**)

TBDMS (*tert*-butyl-dimethylsilyl)-protected 1-hydroxy-1,12-dicarba-*closo*-dodecaborane derivative (**1**) (1.00 eq., 0.50 g, 1.11 mmol) was dissolved in 4.00 mL dry tetrahydrofuran (THF). At 0 °C, a tetra-*n*-butylammonium fluoride (TBAF) solution (1.05 eq., 1.00 M in THF, 1.36 mL, 1.36 mmol) was added dropwise. The reaction mixture was stirred for 20 min at 0 °C. The reaction was then stopped by adding 20 mL distilled water. After dilution with 50 mL EtOAc, the phases were separated. The aqueous phase was extracted three times with 25 mL EtOAc. The combined organic phases were washed with 25 mL brine and dried over magnesium sulfate. The volatiles were removed under reduced pressure and the crude product was purified by column chromatography (SiO_2_, hexane/EtOAc, 2:1, *v*/*v*). Colorless solid, yield 90% (0.32 g, 1.18 mmol); m.p.: 131–132 °C; TLC (hexane/EtOAc, 2:1, *v*/*v*): *R*_f_ = 0.56. ^1^H-NMR (400.16 MHz, CDCl_3_): *δ* [ppm] = 1.56–3.43 (br m, 10H, B**H**), 3.56 (br s, 1H, 7-O**H**), 7.08 (t, ^3^*J*_HH_ = 4.5 Hz, 1H, 2-C**H**), 7.66 (d, ^3^*J*_HH_ = 5.0, 1.1 Hz, 1H, 1-C**H**), 7.83 (d, ^3^*J*_HH_ = 4.0 Hz, 1H, 3-C**H**); ^13^C{^1^H}-NMR (100.63 MHz, CDCl_3_): *δ* [ppm] = 72.1 (6-**C**_q_), 110.5 (7-**C**_q_), 128.4 (2-**C**_q_), 136.1 (1-**C**_q_), 136.4 (3-**C**_q_), 140.3 (4-**C**_q_), 179.6 (5-**C**_q_); ^11^B{^1^H}-NMR (128.37 MHz, CDCl_3_): *δ* [ppm] = −15.1 (s, 5**B**), −13.1 (s, 5**B**); FTIR (ATR): *ṽ* [cm^−1^] = 3328 (b, *ν*_O–H_), 2615 (s, *ν*_B–H_), 2601 (s, *ν*_B–H_), 1646 (s, *ν*_C=O_)_,_ 1629 (s, *ν*_C=O_), 1510 (m, *ν*_C=C_), 1449 (w, *δ*_C–H_), 1403 (s, *δ*_C–H_), 1355 (s, *δ*_C–H_), 1345 (s, *δ*_C–H_), 1261 (s, *ν*_C=S_), 1215 (s),1192 (s, *ν*_C–O_), 1115 (s), 1063 (m), 1035 (w), 1010 (s), 935 (m), 865 (m), 741 (s), 722 (s, *ν*_B–B_), 691 (s), 665 (s), 573 (w), 556 (w), 447 (m); HR-ESI-MS (negative mode, MeOH): *m/z* [M−H]^−^ calcd. for C_7_H_13_B_10_O_2_S: 271.1572, found: 271.1570; elemental analysis: calcd. (%) for C_7_H_14_B_10_O_2_S: C 31.19, H 5.22, found: C 31.04, H 5.21.

#### 3.2.3. 1-(*tert*-Butyl-dimethylsiloxy)-12-formyl-1,12-dicarba-*closo*-dodecaborane(12) (**2**)

1-(*tert*-Butyl-dimethylsiloxy)-1,12-dicarba-*closo*-dodecaborane(12) (1.00 eq., 2.14 g, 7.79 mmol) was dissolved in 100 mL dry diethyl ether. At 0 °C, *n*BuLi (1.05 eq., 1.43 M in hexane, 5.72 mL, 8.18 mmol) was added dropwise. Afterwards, the solution was stirred at room temperature for two hours. At −78 °C, methyl formate (3.50 eq., 2.20 mL, 27.3 mmol) was added dropwise. The solution was first stirred for one hour at −78 °C, then warmed to room temperature and stirred for 24 h. After addition of 30 mL 1 M HCl, the phases were separated. The aqueous phase was extracted three times with 50 mL diethyl ether, and the combined organic phases were washed twice with 25 mL brine and then dried over magnesium sulfate. The solvent was removed under reduced pressure and the crude product was purified by column chromatography (SiO_2_, hexane → hexane/EtOAc, 20:1, *v*/*v*). Colorless solid, yield 86% (2.02 g, 6.68 mmol); m.p.: 40–41 °C; TLC (hexane): *R*_f_ = 0.57. ^1^H-NMR (400.16 MHz, CDCl_3_): *δ* [ppm] = 0.09 (s, 6H, 4-C**H**_3_), 0.75 (s, 9H, 6-C**H**_3_), 1.30–3.36 (br m, 10H, B**H**), 8.96 (s, 1H, 1-C**H**); ^13^C{^1^H}-NMR (100.63 MHz, CDCl_3_): *δ* [ppm] = −4.20 (4-**C**H_3_), 17.7 (5-**C**_q_), 25.1 (6-**C**H_3_), 70.7 (2-**C**_q_), 113.1 (3-**C**_q_), 187.2 (1-**C**_q_); ^11^B{^1^H}-NMR (128.37 MHz, CDCl_3_): *δ* [ppm] = −16.9 (s, 5**B**), −12.5 (s, 5**B**); FTIR (ATR): *ṽ* [cm^−1^] = 2955 (m, *ν*_C–H_), 2930 (m, *ν*_C–H_), 2859 (m, *ν*_C–H_), 2609 (s, *ν*_B–H_), 1970 (w), 1735 (s, *ν*_C=O_), 1471 (m, *δ*_C–H_), 1463 (w, *δ*_C–H_),1390 (w, *δ*_C–H_), 1362 (w, *δ*_C–H_), 1252 (s, *ν*_C–O_), 1168 (s, *ν*_C–O_), 1031 (s, *ν*_C–O_), 1004 (w), 962 (m), 781 (s), 730 (m, *ν*_B-B_), 669 (m), 642 (m), 575 (w), 557 (m); HR-ESI-MS (negative mode, CH_3_CN): *m/z* [M+Cl]^−^, calcd. for C_9_H_26_B_10_ClO_2_Si: 339.2326, found: 339.2302; elemental analysis: calcd. (%) for C_9_H_26_B_10_O_2_Si: C 35.74, H 8.66, found: C 35.70, H 8.67.

#### 3.2.4. 1-(*tert*-Butyl-dimethylsiloxy)-12-(hydroxymethyl-[2′-ethylisothiazolidine-1′,1′-dioxide])-1,12-dicarba-*closo*-dodecaborane(12) (**3**)

2-Ethylisothiazolidine-1,1-dioxide (1.10 eq., 1.08 g, 7.27 mmol) was dissolved in 15 mL dry THF. At −78 °C, *n*BuLi (1.15 eq., 1.36 M in hexane, 5.61 mL, 7.63 mmol) was added dropwise. Afterwards, the solution was stirred at −78 °C for 30 min. At −78 °C, aldehyde **2** (1.00 eq., 2.00 g, 6.61 mmol) was added dropwise. The solution was first stirred for 1.5 h at −78 °C, then it was warmed to room temperature and stopped by adding 15 mL 1 M HCl. The phases were separated, and the aqueous phase was extracted three times with 75 mL diethyl ether. The combined organic phases were washed twice with 25 mL brine and then dried over magnesium sulfate. The solvent was removed under reduced pressure and the crude product was purified by column chromatography (SiO_2_, hexane/EtOAc 3:1 → 2:1, *v*/*v*). Colorless solid, yield 53% (1.58 g, 3.49 mmol); m.p.: 149–150 °C; TLC (hexane/EtOAc 3:1, *v*/*v*): *R*_f_ = 0.55. ^1^H-NMR (400.16 MHz, CDCl_3_): *δ* [ppm] = 0.09 (s, 6H, 9-C**H**_3_), 0.74 (s, 9H, 11-C**H**_3_), 1.29–3.33 (br m, 10H, B**H**), 1.18 (t, ^3^*J*_HH_ = 7.2 Hz, 2H, 1-C**H**_3_), 2.22 (m, 1H, 4-C**H**_2_), 2.37 (m, 1H, 4-C**H**_2_), 2.97 (d, ^3^*J*_HH_ = 3.9 Hz, 2H, 3-C**H**_2_), 3.08 (m, 4H, 2/3-C**H**_2_), 4.33 (d, ^3^*J*_HH_ = 3.6 Hz, 1H, 6-C**H**); ^13^C{^1^H}-NMR (100.63 MHz, CDCl_3_): *δ* [ppm] = −4.3 (9-**C**H_3_), 13.2 (1-**C**H_3_), 17.7 (10-**C**_q_), 18.1 (4-**C**H_2_), 39.4 (5-**C**H), 44.4 (2-**C**H_2_), 60.8 (3-**C**H_2_), 68.1 (6-**C**-OH), 77.3 (7-**C**_q_), 111.6 (8-**C**_q_); ^11^B{^1^H}-NMR (128.37 MHz, CDCl_3_): *δ* [ppm] = −16.0 (s, 5**B**), −12.8 (s, 5**B**); FTIR (ATR): *ṽ* [cm^−1^] = 3447 (b, *ν*_O–H_), 2958 (m, *ν*_C–H_), 2929 (m, *ν*_C-H_), 2858 (m, *ν*_C–H_), 2598 (s, *ν*_B–H_), 1466 (m, *δ*_C–H_), 1409 (w, *δ*_C–H_), 1361 (w, *δ*_C–H_), 1329 (w, *δ*_C–H_), 1284 (s, *ν*_S=O_), 1266 (m), 1216 (s), 1183 (w, *ν*_C–O_), 1129 (s, *ν*_C–O_), 1109 (s, *ν*_C–O_), 1078 (w), 1032 (s), 1004 (w), 989 (m), 953 (w), 940 (m), 887 (w), 837 (w), 783 (s), 741 (s, *ν*_B–B_), 707 (w), 672 (m), 656 (w), 640 (w), 613 (w), 579 (m), 503 (w), 482 (w), 452 (w); HR-ESI-MS (positive mode, MeOH): *m/z* [M + H]^+^, calcd. for C_14_H_38_B_10_NO_4_SSi: 454.3215, found: 454.3234; elemental analysis: calcd. (%) for C_14_H_37_B_10_NO_4_SSi: C 37.23, H 8.26, N 3.10, found: C 37.90, H 8.25, N 3.89.

#### 3.2.5. 1-(*tert*-Butyl-dimethylsiloxy)-12-(*p*-toluenesulfonylmethyl-[2′-ethylisothiazolidine-1′,1′-dioxide])-1,12-dicarba-*closo*-dodecaborane(12) (**4**)

1-(*tert*-Butyl-dimethylsiloxy)-12-(hydroxymethyl-[2′-ethylisothiazolidine-1′,1′-dioxide])-1,12-dicarba-*closo*-dodecaborane(12) (**3**) (1.00 eq., 1.00 g, 2.21 mmol) was dissolved in 7.00 mL dry THF. At 0 °C, NaH (1.05 eq., 60% dispersion in mineral oil, 93.1 mg, 0.69 mmol) was added. The reaction mixture was stirred for two hours at room temperature. At 0 °C, *p*-toluenesulfonyl chloride (2.00 eq., 0.84 g, 4.42 mmol) was added and the solution was stirred for 24 h at room temperature. The reaction was then stopped by adding 30 mL of a 1 M HCl. After dilution with 75 mL diethyl ether, the phases were separated. The aqueous phase was extracted three times with 25 mL diethyl ether. The combined organic phases were washed with 25 mL brine and dried over magnesium sulfate. The volatiles were removed under reduced pressure and the crude product was purified by column chromatography (SiO_2_, hexane/EtOAc, 4:1 → 3:1, *v*/*v*). Colorless solid, yield 87% (1.17 g, 1.93 mmol); m.p.: 181–182 °C; TLC (hexane/EtOAc, 4:1, *v*/*v*): *R*_f_ = 0.54. ^1^H-NMR (400.16 MHz, CDCl_3_): *δ* [ppm] = 0.05 (s, 6H, 13-C**H**_3_), 0.72 (s, 9H, 15-C**H**_3_), 1.09–3.24 (br m, 10H, B**H**), 1.00 (t, ^3^*J*_HH_ = 7.3 Hz, 2H, 1-C**H**_3_), 2.26 (dtd, ^3^*J*_HH_ = 13.1, 7.0, 6.2 Hz, ^4^*J*_HH_ = 3.2 Hz, 2H, 4-C**H**_2_), 2.44 (s, 3H, 10-C**H**_3_), 2.71 (m, 1H, 3-C**H**_2_), 2.82 (m, 1H, 3-C**H**_2_), 2.92 (m, 2H, 2-C**H**_2_), 3.21 (ddd, ^3^*J*_HH_ = 8.6, 6.4 Hz, ^4^*J*_HH_ = 2.3 Hz, 1H, 5-C**H**), 5.29 (d, ^3^*J*_HH_ = 2.3 Hz, 1H, 6-C**H**), 7.31 (d, ^3^*J*_HH_ = 8.1 Hz, 1H, 8-C**H**), 7.77 (d, ^3^*J*_HH_ = 8.2 Hz, 1H, 9-C**H**); ^13^C{^1^H}-NMR (100.63 MHz, CDCl_3_): *δ* [ppm] = −4.3 (13-**C**H_3_), 12.7 (1-**C**H_3_), 17.7 (14-**C**_q_), 20.5 (4-**C**H_2_), 21.8 (10-**C**H_3_), 25.1 (15-**C**H_3_), 38.9 (3-**C**H_2_), 43.5 (2-**C**H_2_), 60.6 (5-**C**H), 70.1 (11-**C**_q_), 77.3 (6-**C**H), 112.6 (12-**C**_q_), 128.5 (9-**C**H), 129.4 (8-**C**H), 134.2 (10-**C**H_3_), 145.0 (7-**C**_q_); ^11^B{^1^H}-NMR (128.37 MHz, CDCl_3_): *δ* [ppm] = −15.8 (s, 5**B**), −12.6 (s, 5**B**); FTIR (ATR): *ṽ* [cm^−1^] = 2956 (m, *ν*_C–H_), 2930 (m, *ν*_C–H_), 2857 (m, *ν*_C–H_), 2619 (s, *ν*_B–H_), 2592 (s, *ν*_B–H_), 1595 (w), 1463 (w, *δ*_C–H_), 1450 (w, *δ*_C–H_), 1372 (m, *δ*_C–H_), 1341 (w, *δ*_C–H_), 1293 (m, *δ*_C–H_), 1254 (s, *ν*_S=O_), 1189 (m, *ν*_C–O_),1172 (s, *ν*_C-O_), 1132 (s), 1109 (s), 1095 (w), 1076 (w), 1034 (w), 1019 (w), 1005 (w), 972 (s), 894 (m), 870 (w), 838 (s), 810 (s), 783 (s), 751 (s, *ν*_B–B_), 720 (w), 670 (s), 639 (w), 631 (w), 577 (m), 559 (m), 544 (s), 498 (w), 455 (w), 442 (w), 435 (w), 429 (w); HR-ESI-MS (positive mode, MeOH): *m/z* [M + H]^+^, calcd. for C_21_H_44_B_10_NO_6_S_2_Si: 608.3304, found: 608.3350; elemental analysis: calcd. (%) for C_21_H_43_B_10_NO_6_S_2_Si: C 41.63, H 7.51, N 2.31 found: C 42.18, H 7.28, N 2.22.

#### 3.2.6. (3*E*)-1-(*tert*-Butyl-dimethylsiloxy)-12-(methylene-[2′-ethylisothiazolidine-1′,1′-dioxide])-1,12-dicarba-*closo*-dodecaborane(12) (**5**)

1-*(tert-*Butyl-dimethylsiloxy*)-*12-(*p-*toluenesulfonylmethyl-[2′-ethylisothiazolidine-1′,1′-dioxide])*-*1,12-dicarba-*closo*-dodecaborane(12) (**4**) (1.00 eq., 0.46 g, 0.75 mmol) was dissolved in 3.80 mL dry THF. At 0 °C, NaH (1.00 eq., 60% dispersion in mineral oil, 30.4 mg, 0.75 mmol) was added. The reaction mixture was stirred for two hours at room temperature. The reaction was then stopped by adding 25 mL distilled water. After dilution with 25 mL EtOAc, the phases were separated. The aqueous phase was extracted three times with 25 mL EtOAc. The combined organic phases were washed with 25 mL brine and dried over magnesium sulfate. The volatiles were removed under reduced pressure and the crude product was purified by column chromatography (SiO_2_, hexane/EtOAc, 4:1 → 0:1, *v*/*v*). Colorless solid, yield 64% (0.21 g, 0.48 mmol); m.p.: 147–148 °C; TLC (hexane/EtOAc, 4:1, *v*/*v*): *R*_f_ = 0.50. ^1^H-NMR (400.16 MHz, CDCl_3_): *δ* [ppm] = 0.08 (s, 6H, 9-C**H**_3_), 0.75 (s, 9H, 11-C**H**_3_), 1.14–3.34 (br m, 10H, B**H**), 1.23 (t, ^3^*J*_HH_ = 7.2 Hz, 2H, 1-C**H**_3_), 2.89 (td, ^3^*J*_HH_ = 6.6 Hz, ^4^*J*_HH_ = 2.9 Hz, 2H, 4-C**H**_2_), 3.08 (q, ^3^*J*_HH_ = 7.3 Hz, 2H, 2-C**H**_2_), 3.14 (^3^*J*_HH_ = 6.6 Hz, 2H, 3-C**H**_2_), 6.09 (t, ^4^*J*_HH_ = 2.8 Hz, 1H, 6-C**H**); ^13^C{^1^H}-NMR (100.63 MHz, CDCl_3_): *δ* [ppm] = −4.4 (9-**C**H_3_), 12.7 (1-**C**H_3_), 17.6 (10-**C**_q_), 21.9 (4-**C**H_2_), 24.9 (11-**C**H_3_), 39.1 (2-**C**H_2_), 42.8 (3-**C**H_2_), 63.9 (7-**C**_q_), 112.6 (12-**C**_q_), 111.8 (8-**C**_q_), 126.9 (6-**C**H), 140.5 (5-**C**_q_); ^11^B{^1^H}-NMR (128.37 MHz, CDCl_3_): *δ* [ppm] = −15.0 (s, 5**B**), −12.4 (s, 5**B**); FTIR (ATR): *ṽ* [cm^−1^] = 2948 (m, *ν*_C–H_), 2930 (m, *ν*_C–H_), 2859 (w, *ν*_C–H_), 2619 (s, *ν*_B–H_), 1667 (w, *ν*_C=C_), 1471 (w, *δ*_C–H_), 1461 (m, *δ*_C–H_), 1422 (w, *δ*_C–H_), 1390 (m, *δ*_C–H_), 1361 (w, *δ*_C–H_), 1309 (m, *δ*_C–H_), 1297 (s, *ν*_S=O_), 1262 (s, *ν*_S=O_), 1250 (s, *ν*_S=O_), 1208 (m, *ν*_C–O_),1189 (m, *ν*_C–O_), 1172 (m), 1156 (m), 1136 (w), 1102 (w), 1073 (m), 1037 (s), 1023 (m), 1005 (w), 972 (w), 939 (m), 837 (s), 781 (s), 750 (w), 729 (s, *ν*_B-B_), 698 (m), 670 (m), 643 (w), 596 (m), 572 (m), 561 (m), 533 (s), 509 (s), 466 (m), 448 (m), 441 (w), 435 (w); HR-ESI-MS (positive mode, MeOH): *m/z* [M + H]^+^, calcd. for C_14_H_36_B_10_NO_3_SSi: 436.3110, found: 436.3120; elemental analysis: calcd. (%) for C_14_H_35_B_10_NO_3_SSi: C 38.77, H 8.13, N 3.23 found: C 39.49, H 8.27, N 2.98.

#### 3.2.7. (3*E*)-1-Hydroxy-12-(methylene-[2′-ethylisothiazolidine-1′,1′-dioxide])-1,12-dicarba-*closo*-dodecaborane(12) (**S-2474-Cb**)

(3*E*)*-*1-*(tert-*Butyl-dimethylsiloxy)-12-(methylene-[2′-ethylisothiazolidine-1′,1′-dioxide])-1,12-dicarba-*closo*-dodecaborane(12) (**5**) (1.00 eq., 100 mg, 0.23 mmol) was dissolved in 0.80 mL dry THF. At 0 °C, a TBAF solution (1.05 eq., 1.00 M in THF, 0.24 mL, 0.24 mmol) was added dropwise. The reaction mixture was stirred for 20 min at 0 °C. The reaction was then stopped by adding 10 mL distilled water. After dilution with 25 mL EtOAc the phases were separated. The aqueous phase was extracted three times with 25 mL EtOAc. The combined organic phases were washed twice with 20 mL brine and dried over magnesium sulfate. The volatiles were removed under reduced pressure and the crude product was purified by column chromatography (SiO_2_, hexane/EtOAc, 1:1 → 0:1, *v*/*v*). Colorless solid, yield 96% (70.0 mg, 0.22 mmol); m.p.: 220–222 °C; TLC (hexane/EtOAc, 2:1, *v*/*v*): *R*_f_ = 0.46. ^1^H-NMR (400.16 MHz, acetone-d_6_): *δ* [ppm] = 1.48–3.30 (br m, 10H, B**H**), 1.17 (t, ^3^*J*_HH_ = 7.3 Hz, 2H, 1-C**H**_3_), 2.95 (dt, ^3^*J*_HH_ = 6.5 Hz, ^4^*J*_HH_ = 3.3 Hz, 2H, 4-C**H**_2_), 3.00 (q, ^3^*J*_HH_ = 7.3 Hz, 2H, 2-C**H**_2_), 3.20 (t, ^3^*J*_HH_ = 6.5 Hz, 2H, 3-C**H**_2_), 5.96 (t, ^4^*J*_HH_ 2.9 Hz, 1H, 6-C**H**); ^13^C{^1^H}-NMR (100.63 MHz, acetone-d_6_): *δ* [ppm] = 13.2 (1-**C**H_3_), 22.9 (4-**C**H_2_), 40.0 (2-**C**H_2_), 43.8 (3-**C**H_2_), 65.0 (7-**C**_q_), 111.8 (8-**C**_q_), 126.0 (6-**C**H), 143.6 (5-**C**_q_); ^11^B{^1^H}-NMR (128.37 MHz, acetone-d_6_): *δ* [ppm] = −14.7 (s, 5**B**), −12.7 (s, 5**B**); FTIR (ATR): *ṽ* [cm^−1^] = 3284 (b, *ν*_OH_), 2987 (m, *ν*_C–H_), 2933 (m, *ν*_C–H_), 2880 (m, *ν*_C–H_), 2611 (s, *ν*_B–H_), 2595 (s, *ν*_B–H_), 1665 (m, *ν*_C=C_), 1466 (w, *δ*_C–H_), 1454 (m, *δ*_C–H_), 1423 (w, *δ*_C–H_), 1389 (m, *δ*_C–H_), 1352 (w, *δ*_C–H_), 1278 (s, *ν*_C–O_), 1235 (w, *ν*_C–O_), 1151 (s, *ν*_S=O_), 1132 (s, *ν*_S=O_), 1098 (s), 1023 (s), 959 (m), 935 (w), 897 (w), 832 (m), 795 (m), 728 (s, v_B-B_), 700 (w), 602 (m), 570 (w), 546 (s), 507 (s), 487 (m), 457 (m); HR-ESI-MS (positive mode, MeOH): *m/z* [M + H]^+^, calcd. for C_8_H_22_B_10_NO_2_S: 322.2245, found: 322.2255; elemental analysis: calcd. (%) for C_8_H_21_B_10_NO_2_S: C 30.08, H 6.63, N 4.39, found: C 30.05, H 6.53, N 4.42.

#### 3.2.8. (3*E*)-1-(*tert*-Butyldimethylsiloxy)-12-(methylene-[dihydrofurane-2′(3*H*)-one])-1,12-dicarba-*closo*-dodecaborane(12) (**6**)

Aldehyde **2** (1.00 eq., 0.70 g, 2.31 mmol) and 3-(triphenylphosphoranylidene)-*γ*-butyrolactone(1.50 eq., 1.20 g, 3.47 mmol) were dissolved in 11 mL dry THF and 1.40 mL dry (*N*,*N*)-dimethylformamide (DMF). The reaction mixture was heated under reflux for 48 h. After cooling to room temperature, the solvent was removed under reduced pressure. The residue was then dissolved in 100 mL EtOAc and 75 mL distilled water. The phases were separated and the aqueous phase was extracted three times with 50 mL EtOAc. The combined organic phases were washed twice with 50 mL brine and then dried over magnesium sulfate. The solvent was removed under reduced pressure and the crude product was purified by column chromatography (SiO_2_, hexane/EtOAc, 5:1 → 3:1, *v*/*v*). Colorless solid, yield 84% (0.72 g, 1.94 mmol); m.p.: 149–151 °C; TLC (hexane/EtOAc, 5:1, *v*/*v*): *R*_f_ = 0.62. ^1^H-NMR (400.16 MHz, CDCl_3_): *δ* [ppm] = 0.08 (s, 6H, 8-C**H**_3_), 0.74 (s, 9H, 10-C**H**_3_), 1.34–3.31 (br m, 10H, B**H**), 2.95 (td, ^3^*J*_HH_ = 7.2 Hz; ^4^*J*_HH_ = 3.0 Hz, 2H, 3-C**H**_2_), 4.30 (t, ^3^*J*_HH_ = 7.2 Hz, 2H, 1-C**H**_2_), 6.38 (t, ^4^*J*_HH_ = 3.1 Hz, 1H, 5-C**H**); ^13^C{^1^H}-NMR (100.63 MHz, CDCl_3_): *δ* [ppm] = −4.2 (8-**C**H_3_), 17.7 (9-**C**_q_), 25.1 (3-**C**H_3_), 64.7 (6-**C**_q_), 65.5 (1-**C**H_2_), 111.8 (7-**C**_q_), 128.6 (4-**C**_q_), 134.0 (5-**C**H), 170.8 (2-**C**_q_); ^11^B{^1^H}-NMR (128.37 MHz, CDCl_3_): *δ* [ppm] = −15.1 (s, 5**B**), −12.5 (s, 5**B**); FTIR (ATR): *ṽ* [cm^−1^] = 2925 (m, *ν*_C–H_), 2885 (w, *ν*_C–H_), 2856 (m, *ν*_C–H_), 2608 (s, *ν*_B–H_), 1725 (s, *ν*_C=O_), 1676 (m, v_c=c_), 1470 (m, *δ*_C–H_), 1428 (w, *δ*_C–H_), 1380 (m, *δ*_C–H_), 1360 (m, *δ*_C–H_), 1350 (m, *δ*_C–H_), 1261 (s, *ν*_C–O–C_), 1261 (s, *ν*_C–O–C_), 1250 (s, *ν*_C–O–C_), 1207 (s), 1072 (m), 1029 (s), 965 (m), 939 (w), 836 (s), 779 (s), 781 (s), 736 (m, v_B–B_), 695 (m), 646 (m), 628 (w), 574 (m), 517 (m), 461 (m); HR-ESI-MS (positive mode, CH_3_CN): *m/z* [M + NH_4_]^+^, calcd. for C_13_H_34_B_10_NO_3_Si: 390.3233, found: 390.3228; elemental analysis: calcd. (%) for C_13_H_30_B_10_O_3_Si: C 42.14, H 8.16, found: C 42.22, H 8.04.

#### 3.2.9. (3*E*)-1-Hydroxy-12-(methylene-[dihydrofurane-2′(3*H*)-one])-1,12-dicarba-*closo*-dodecaborane(12) (**KME-4-Cb**)

(3*E*)-1-(*tert*-Butyl-dimethylsiloxy)-12-(methylene-[dihydrofuran-2′(3*H*)-one])-1,12-dicarba-*closo*-dodecaborane(12) (**6**) (1.00 eq., 0.29 g, 0.78 mmol) was dissolved in 2.50 mL dry THF. At 0 °C, a TBAF solution (1.05 eq., 1.00 M in THF, 0.82 mL, 0.82 mmol) was added dropwise. The reaction mixture was stirred for 25 min at 0 °C. The reaction was then stopped by adding 20 mL distilled water. After dilution with 25 mL EtOAc the phases were separated. The aqueous phase was extracted three times with 25 mL EtOAc. The combined organic phases were washed with 20 mL brine and dried over magnesium sulfate. The volatiles were removed under reduced pressure and the crude product was purified by column chromatography (SiO_2_, hexane/EtOAc, 1:1, *v*/*v*). Colorless solid, yield 99% (0.19 g, 0.77 mmol); m.p.: 210–212 °C; TLC (hexane/EtOAc, 1:1, *v*/*v*): *R*_f_ = 0.62. ^1^H-NMR (400.16 MHz, acetone-d_6_): *δ* [ppm] = 1.47–3.27 (br m, 10H, B**H**), 3.03 (td, ^3^*J*_HH_ = 7.2 Hz, ^4^*J*_HH_ = 3.1 Hz, 2H, 3-C**H**_2_), 4.35 (t, ^3^*J*_HH_ = 7.1 Hz, 2H, 1-C**H**_2_), 6.20 (t, ^4^*J*_HH_ = 3.1 Hz, 1H, 5-C**H**); ^13^C{^1^H}-NMR (100.63 MHz, acetone-d_6_): *δ* [ppm] = 26.0 (3-**C**H_3_), 66.0 (6-**C**_q_), 66.3 (1-**C**H_2_), 111.5 (7-**C**_q_), 131.5 (4-**C**_q_), 132.1 (5-**C**H), 170.9 (2-**C**_q_); ^11^B{^1^H}-NMR (128.37 MHz, acetone-d_6_): *δ* [ppm] = −14.8 (s, 5**B**), −12.6 (s, 5**B**); FTIR (ATR): *ṽ* [cm^−1^] = 3157 (b, *ν*_O-H_), 2977 (w, *ν*_C–H_), 2920 (w, *ν*_C–H_), 2603 (s, *ν*_B–H_), 1727 (s, *ν*_C=O_), 1672 (m, *ν*_C=C_), 1577 (w, *ν*_C=C_), 1480 (m, *δ*_C–H_), 1452 (w, *δ*_C–H_), 1427 (m, *ν*_C=C_), 1387 (s), 1358 (s, *δ*_C–H_),1282 (w, *ν*_C–O_), 1209 (s, *ν*_C–O–C_), 1088 (m), 1032 (s), 976 (m), 866 (w), 776 (w), 754 (w), 732 (w), 715 (w), 693 (m), 634 (m), 588 (w), 572 (w), 524 (w), 483 (w); HR-ESI-MS (positive mode, CH_3_CN): *m/z* [M + H]^+^, calcd. for C_7_H_17_B_10_O_3_: 257.2102, found: 257.2184; elemental analysis: calcd. (%) for C_7_H_16_B_10_O_3_: C 32.80, H 6.29, found: C 32.19, H 6.20.

#### 3.2.10. (3*E*)-1-(*tert*-Butyl-dimethylsiloxy)-12-(methylene-[1′-methoxypyrrolidine-2′-one])-1,12-dicarba-*closo*-dodecaborane(12) (**7**)

(1-Methoxy-2-oxo-3-pyrrolidinyl)triphenyl-phosphonium bromide (1.50 eq., 0.95 g, 2.08 mmol) and NEt_3_ (1.50 eq., 0.29 mL, 2.08 mmol) were dissolved in 7.00 mL dry THF. The solution was stirred for 45 min at room temperature. Afterwards, a solution of aldehyde **2** (1.00 eq., 0.38 g, 1.38 mmol) in 2.00 mL dry DMF was added dropwise. The reaction mixture was stirred for 24 h at 75 °C. After cooling to room temperature, the solvent was removed under reduced pressure. The residue was then dissolved in 50 mL EtOAc and 30 mL distilled water. The phases were separated, and the aqueous phase was extracted three times with 25 mL EtOAc. The combined organic phases were washed twice with 30 mL brine and then dried over magnesium sulfate. The solvent was removed under reduced pressure and the crude product was purified by column chromatography (SiO_2_, hexane/EtOAc, 1:1 → 0:1, *v*/*v*). Colorless solid, yield 50% (0.28 g, 0.69 mmol); m.p.: 168–170 °C; TLC (hexane/EtOAc, 1:1, *v*/*v*): *R*_f_ = 0.60. ^1^H-NMR (400.16 MHz, CDCl_3_): *δ* [ppm] = 0.07 (s, 6H, 9-C**H**_3_), 0.74 (s, 9H, 11-C**H**_3_), 1.42–3.30 (br m, 10H, B**H**), 2.79 (td, ^3^*J*_HH_ = 6.4 Hz, ^4^*J*_HH_ = 2.9 Hz, 2H, 4-C**H**_2_), 3.50 (t, ^3^*J*_HH_ = 6.4 Hz, 2H, 3-C**H**_2_), 3,78 (s, 3H, 1-C**H**_3_), 6.19 (t, ^4^*J*_HH_ = 2,8 Hz, 1H, 6-C**H**); ^13^C{^1^H}-NMR (100.63 MHz, CDCl_3_): *δ* [ppm] = −4.2 (9-**C**H_3_), 17.7 (10-**C**_q_), 21.1 (4-**C**H_2_), 25.1 (11-**C**H_3_), 42.8 (3-**C**H_2_), 62.2 (1-**C**H_3_), 65.2 (7-**C**_q_), 111.3 (8-**C**_q_), 128.5 (6-**C**_q_), 131.3 (5-**C**_q_), 163.7 (2-**C**_q_); ^11^B{^1^H}-NMR (128.37 MHz, CDCl_3_): *δ* [ppm] = −15.0 (s, 5**B**), −12.5 (s, 5**B**); FTIR (ATR): *ṽ* [cm^−1^] = 2953 (w, *v*_C–H_), 2932 (m, *ν*_C–H_), 2896 (w, *ν*_C–H_), 2858 (w, *ν*_C–H_), 2603 (s, *ν*_B–H_), 1994 (w), 1708 (s, *ν*_C=O_), 1672 (m, *v*_C=C_), 1472 (m, *v*_C=C_), 1461 (w, *δ*_C–H_), 1428 (w, *δ*_C–H_), 1399 (w, *δ*_C–H_), 1362 (w, *δ*_C–H_), 1344 (m, *δ*_C–H_), 1249 (s, *v*_C–O–C_), 1119 (w), 1038 (s), 1003 (w), 970 (m), 926 (m), 837 (s), 779 (s), 731 (m, *v*_B–B_), 694 (m), 666 (w), 628 (w), 603 (m), 521 (w), 482 (m), 466 (m), 449 (m), 431 (m), 461 (m); HR-ESI-MS (positive mode, CH_3_CN): *m/z* [M + H]^+^, calcd. for C_14_H_34_B_10_NO_3_Si: 402.3233, found: 402.3248; elemental analysis: calcd. (%) for C_14_H_33_B_10_NO_3_Si: C 42.08, H 8.32, N 3.51, found: C 42.39, H 8.07, N 3.53.

#### 3.2.11. (3*E*)-1-Hydroxy-12-(methylene-[1′-methoxypyrrolidine-2′-one])-1,12-dicarba-*closo*-dodecaborane(12) (**E-5110-Cb**)

(3*E*)-1-(*tert*-Butyl-dimethylsiloxy)-12-(methylene-[1′-methoxypyrrolidine-2′-one])-1,12-dicarba-*closo*-dodecaborane(12) (**7**) (1.00 eq., 0.18 g, 0.45 mmol) was dissolved in 1.50 mL dry THF. At 0 °C, a TBAF solution (1.05 eq., 1.00 M in THF, 0.47 mL, 0.47 mmol) was added dropwise. The reaction mixture was stirred for 30 min at 0 °C. The reaction was then stopped by adding 20 mL distilled water. After dilution with 25 mL EtOAc the phases were separated. The aqueous phase was extracted three times with 25 mL EtOAc. The combined organic phases were washed twice with 20 mL brine and dried over magnesium sulfate. The volatiles were removed under reduced pressure and the crude product was purified by column chromatography (SiO_2_, hexane/EtOAc, 2:1, *v*/*v*). Colorless solid, yield 93% (0.12 g, 0.42 mmol); m.p.: 222–224 °C; TLC (hexane/EtOAc, 2:1, *v*/*v*): *R*_f_ = 0.52. ^1^H-NMR (400.16 MHz, acetone-d_6_): *δ* [ppm] = 1.48–3.28 (br m, 10H, B**H**), 2.84 (ddd, ^3^*J*_HH_ = 6.8, 5.9 Hz, ^4^*J*_HH_ = 2.9 Hz, 2H, 4-C**H**_2_), 3.58 (dd, ^3^*J*_HH_ = 6.7, 5.9 Hz, 2H, 3-C**H**_2_), 3.73 (s, 3H, 1-C**H**_3_), 5.99 (t, ^4^*J*_HH_ = 2.9 Hz, 1H, 6-C**H**); ^13^C{^1^H}-NMR (100.63 MHz, acetone-d_6_): *δ* [ppm] = 21.9 (4-**C**H_2_), 43.3 (3-**C**H_2_), 62.1 (1-**C**H_3_), 66.4 (7-**C**_q_), 111.1 (8-**C**_q_), 126.8 (6-**C**_q_), 134.5 (5-**C**_q_), 163.2 (2-**C**_q_); ^11^B{^1^H}-NMR (128.37 MHz, acetone-d_6_): *δ* [ppm] = −14.7 (s, 5**B**), −12.6 (s, 5**B**); FTIR (ATR): *ṽ* [cm^−1^] = 3165 (b, *v*_O–H_), 2941 (w, *v*_C–H_), 2603 (s, *ν*_B–H_), 1686 (s, *ν*_C=O_), 1662 (s, *ν*_C=O_), 1487 (w, *v*_C=C_), 1458 (w, *δ*_C–H_), 1439 (m, *δ*_C–H_), 1393 (w, *δ*_C–H_), 1355 (w, *δ*_C-H_), 1286 (m), 1215 (s), 1126 (m), 1036 (s), 1003 (m), 955 (m), 923 (w), 894 (w), 840 (w), 749 (w, *v*_B–B_), 692 (s), 618 (s), 571 (w), 522 (w), 489 (w); HR-ESI-MS (positive mode, MeOH): *m/z* [M + H]^+^, calcd. for C_8_H_20_B_10_NO_3_: 288.2368, found: 288.2388; elemental analysis: calcd. (%) for C_8_H_19_B_10_NO_3_: C 33.67, H 6.71, N 4.91, found: C 33.22, H 6.68, N 4.80.

### 3.3. HPLC Measurements

#### 3.3.1. Analysis of Purity Ultra Performance Liquid Chromatography (UPLC)

Samples were monitored at 254 nm using the following UPLC system: column Aquity UPLC^®^ BEH C18 column (waters, 150 × 2.1 mm, 1.7 µM, 130 Å), UPLC (waters, Milford, MA, USA): binary solvent manager UPB, sample manager UPA, column manager UPM, and diode array detector PDA, *γ* detector Gabi Star (Raytest), flow rate 0.4 mL/min, eluent: (A) 0.1% trifluoroacetic acid in H_2_O, (B) MeCN; gradient: *t*_0 min_ 95/5—*t*_0.3 min_ 95/5—*t*_5.3 min_ 5/95—*t*_6.5 min_ 5/95—*t*_6.8 min_ 95/5—*t*_10 min_ 95/5). All compounds were found to have a purity of >97% (see Supplementary Materials, Figures S21–S23).

#### 3.3.2. Determination of Lipophilicity (log*D*) by HPLC

The log*D*_7.4, HPLC_ value was determined as previously reported by us [95] utilizing an HPLC method originally described by Donovan and Pescatore [82]. The following HPLC system was used: Agilent 1100 HPLC (binary pump G1312A, autosampler G1313A, column oven G1316A, degasser G1322A, UV detector G1314A, *γ* detector Gabi Star (Raytest); column ODP-50 4B (Shodex Asahipak 50 × 4.6 mm) with C18 pre-column; eluent: MeOH/phosphate buffer (10 mM, pH 7.4), gradient *t*_0_ min 30/70—*t*_25 min_ 95/5—*t*_27 min_ 95/5—*t*_28 min_ 30/70—*t*_40 min_ 30/70, flow rate = 0.6 mL/min. Oxycarboxin (t_R_ 9.02 min, log*D*_7.4_ 1.13) and triphenylene (*t*_R_ 29.47 min, log*D*_7.4_ 5.49) served as references. Toluene was used as control and log*D*_7.4_ was found to be 2.72 (literature log*D*_7.4_ 2.72) [81].

### 3.4. COX Inhibition Studies

COX inhibition activity against ovine COX-1 and human COX-2 was determined using the fluorescence-based COX assay COX Fluorescent Inhibitor Screening Assay Kit (Cayman Chemical Company, Ann Arbor, MI, USA) according to the manufacturer’s instructions as previously reported by us. All compounds were screened at a concentration of 100 µM in duplicate [76].

### 3.5. 5-LO Inhibitory Studies

For the determination of 5-LO inhibitory activities in intact cells, freshly isolated polymorphonuclear leukocytes (PMNL) (5 × 10^6^) were re-suspended in phosphate-buffered saline (PBS) (pH 7.4) containing 1 mg/mL glucose and 1 µM CaCl_2_. After preincubation with **R-830**, **S-2474**, **KME-4**, and **E-5110** in DMSO for 15 min at 37 °C, 5-LO product formation was stimulated by addition of calcium ionophore A23187 (2.5 µM in MeOH) and exogenous arachidonic acid (20 µM in EtOH). After 10 min at 37 °C, the reaction was stopped by addition of ice-cold methanol (1 mL). HCl (30 μL, 1 M), prostaglandin B_1_ (200 ng) and PBS (500 μL) were added and the formed metabolites were extracted and analyzed by HPLC as described previously [96]. 5-LO product formation was determined as the number of 5-LO products produced (nanograms) per 10^6^ cells, which includes leukotriene B_4_ (LTB_4_), its all-*trans* isomers and 5-hydroperoxyicosatetraenoic acid (5-H(_P_)ETE). Cysteinyl LTs C_4_, D_4_, and E_4_ as well as oxidation products of LTB_4_ were not detected. Data were normalized to vehicle control (DMSO) and IC_50_ values and 95% confidence intervals (CIs) of at least three independent measurements were calculated (Table 2, see Supplementary Materials, Table S2, Figures S25 and S26). The selective 5-LO inhibitor BWA4C (0.1 µM) was used as control and inhibited 5-LO product formation by 92.5% ± 0.9.

### 3.6. Cell Viability Studies—MTT and CV Assays

All cell lines were seeded overnight and treated with **R-830**, **R-830-Cb**, **KME-4**, **KME-4-Cb**, **E-5110**, **E-5110-Cb**, **S-2474**, and **S-2474-Cb** for 72 h. After the incubation period, the supernatant was discarded from the wells, and the cells were washed two times with 200 µL of PBS. Next, MTT solution at a final concentration of 0.5 mg/mL was added and incubated at 37 °C until purple formazan crystals were formed (30 min to one hour). After incubation, the dye was discarded. In order to dissolve the formed formazan crystals DMSO was added, and the absorbance was measured at *λ*_max_ = 540 nm, with the reference/background wavelength of 670 nm. All results are expressed as a percentage of the control value which was arbitrary set to 100%. After 72 h treatment, cells were washed with 200 µL of PBS and fixed with 4% paraformaldehyde (PFA) for 15 min at room temperature (RT). Next, cells were stained with 1% CV solution. After 20 min, cells were washed in tap water and dried on air. Dye was dissolved in 33% acetic acid and the absorbance was measured at *λ*_max_ = 540 nm, with the reference/background wavelength of 670 nm. Results were expressed as a percentage of the control value which was arbitrary set to 100%.

### 3.7. Statistical Analyses

All experiments were repeated at least three independent times and data presented represents the means  ±  SD of replicates. To evaluate the significance between groups Student’s *t*-test was used and two-sided *p* values of less than 0.05 were considered statistically significant.

Further materials, methods, and procedures for physicochemical characterization and biological evaluation: see Supplementary Materials (i.e., Supplementary Materials) for further details.

## 4. Conclusions

The substitution of the bulky di-*tert*-butylphenyl moiety in the potent dual COX-2/5-LO inhibitors **R-830**, **KME-4**, **E-5110**, and **S-2474** by *p*-carborane gave rise to the carborane-based analogs **R-830-Cb**, **KME-4-Cb**, **E-5110-Cb**, and **S-2474-Cb**. Replacing the bulky but also hydrophobic di-*tert*-butylphenyl moiety with the hydrophobic boron cluster resulted in a significant decrease in lipophilicity, suggesting that intermolecular interactions, such as hydrogen bonds or dihydrogen bonds, are contributing to the compounds’ lipophilicity [97]. The carborane analogs show no or only weak COX inhibition, as anticipated based on previous reports about the anti-inflammatory activity of related mono- or di-*tert*-butylphenols.

However, in vitro studies on intact cells revealed that all carborane-based analogs are strong inhibitors of 5-LO product formation with IC_50_ values in the nanomolar range, comparable to the respective organic counterparts, indicating that the bioisosteric replacement of the di-*tert*-butylphenyl motif by *p*-carborane is well tolerated.

Furthermore, the introduction of *p*-carborane generally decreased the cytotoxic potential of dual inhibitors, except in the case of **R-830**. Interestingly, the *p*-carborane analog **R-830-Cb** was non-toxic to primary cells but effective against tested cell lines derived from various types of inflammation-related tumors. Its cytotoxic potential was mediated by potent inhibition of ROS, resulting in inhibited proliferation and caspase-dependent apoptosis.

Thus, carborane-based analog **R-830-Cb** is a promising candidate for further assessment and detailed mechanistic as well as in vivo studies.

## Figures and Tables

**Table 1:** Results of COX assay and log*D*_7.4, HPLC_ determination.

Compound	% Inhibition at 100 µM ^(A,B)^		log*D*_7.4, HPLC_
	COX-1	COX-2	
**R-830**	93	85	3.91
**R-830-Cb**	18	6	2.37
**KME-4**	64	97	3.79
**KME-4-Cb**	26	n.i.	1.87
**E-5110**	66	105	3.35
**E-5110-Cb**	n.i.	n.i.	1.50
**S-2474**	38	86	3.75
**S-2474-Cb**	n.i.	n.i.	1.88

^(A)^ n.i. = no inhibition (%inhibition below 5%); ^(B)^ Celecoxib served as reference for COX-2: pIC_50_ *(*pIC_50_ = −log_10_ (IC_50_ [M])) was found to be 7.05 ± 0.08 (mean ± SD, *n* = 5, resembles IC_50_ of 89 nM); SC-560 served as reference for COX-1: pIC_50_ was found to be 8.07 ± 0.63 (mean ± SD, *n* = 3, resembles IC_50_ of 8.4 nM).

**Table 2:** IC_50_ values for inhibition of 5-LO product formation in intact PMNL for the carborane derivatives **R-830-Cb**, **KME-4-Cb**, **E-5110-Cb**, **S-2474-Cb**, and their respective reference compounds **R-830**, **KME-4**, **E-5110**, and **S-2474** (see Supplementary Materials, Figures S25 and S26 and (for 95% CIs) Table S2).

Compound	IC_50_ [µM] ^(A,B)^	Compound	IC_50_ [µM]
**R-830-Cb**	0.65	**R-830**	0.26
**KME-4-Cb**	0.07	**KME-4**	0.15
**E-5110-Cb**	0.22	**E-5110**	0.12
**S-2474-Cb**	<0.03	**S-2474**	<0.03

^(A)^ The selective 5-LO inhibitor BWA4C (0.1 µM) was used as control and inhibited 5-LO product formation by 92.5% ± 0.9; ^(B)^ Data are presented as the mean of at least three independent experiments with 95% CIs (CIs = confidence intervals).

**Table 3:** IC_50_ values [µM] of di-*tert*-butylphenol derivatives and their carborane analogs for specific cancer cell lines. Data are presented as mean ± SD of three independent experiments for 3-(4,5-dimethylthiazol-2-yl)-2,5-diphenyltetrazolium bromide (MTT) and crystal violet (CV) tests. * Carbon-based reference compounds.

Compounds	Assays	A375	A549	HCT116	HT-29	MDA-MB-231
R-830 *	MTT	16.7 ± 3.1	19.0 ± 0.7	20.6 ± 0.4	19.4 ± 0.4	33.3 ± 1.2
	CV	29.4 ± 2.3	32.6 ± 1.3	30.8 ± 3.2	24.9 ± 1.3	34.0 ± 1.6
**R-830-Cb**	MTT	35.9 ± 0.8	66.5 ± 1.7	40.9 ± 3.1	65.6 ± 5.3	46.5 ± 0.1
	CV	47.2 ± 0.7	77.3 ± 1.7	49.1 ± 6	73.3 ± 3.4	55.6 ± 0.7
KME-4 *	MTT	13.8 ± 0.6	21.9 ± 0.7	18.4 ± 4.6	18.5 ± 1.5	80.9 ± 7.3
	CV	36.4 ± 1.3	36.3 ± 2.8	36.2 ± 1.9	36.2 ± 1.9	88.6 ± 2.4
**KME-4-Cb**	MTT	83.3 ± 2.3	119.7 ± 7.0	88.5 ± 4.3	139.3 ± 5.4	119.7 ± 3.9
	CV	155.2 ± 5.8	157.8 ± 3.0	127.0 ± 8.0	164.7 ± 8.5	132.7 ± 4.1
E-5110 *	MTT	12.9 ± 1.1	22.1 ± 1.6	25.1 ± 1.1	25.6 ± 2.6	34.2 ± 1.2
	CV	33.5 ± 1.7	41.1 ± 4.3	39.6 ± 2.8	39.8 ± 2.1	47.3 ± 1.7
**E-5110-Cb**	MTT	69.3 ± 1.7	176.6 ± 14.5	143.9 ± 10.5	119.9 ± 9.7	200 ± 0.0
	CV	103.2 ± 10.9	197.7 ± 5.7	193.1 ± 3.0	155.4 ± 6.0	>200
S-2474 *	MTT	13.2 ± 2.7	16.2 ± 1.2	17.5 ± 1.0	16.1 ± 1.1	34.8 ± 1.7
	CV	19.2 ± 0.7	17.9 ± 1.2	23.7 ± 1.9	23.8 ± 1.7	43.5 ± 1.8
**S-2474-Cb**	MTT	106.0 ± 6.3	121.8 ± 10.4	108.1 ± 11.5	81.8 ± 6.1	>200
	CV	135.5 ± 6.3	149.7 ± 4.3	133.4 ± 7.1	137.5 ± 7.8	>200

## Data Availability

The data presented in this study are available in the article or supplementary Materials.

## Supplementary Materials

The following Supplementary Materials can be downloaded at https://www.mdpi.com/article/10.3390/molecules28114547/s1. Section 1: General methods, materials, and procedures; Section 2: Chemical structures of intermediate compounds and selected spectra of analogs **R-830-Cb**, **KME-4-Cb**, **E-5110-Cb**, and **S-2474-Cb**; Section 3: Determination of purity by HPLC measurements; Section 4: NMR spectroscopic stability studies; Section 5: Determination of lipophilicity (log*D*) by HPLC; Section 6: Biological evaluation; Section 7: Single-crystal X-ray diffraction data for compounds **1**, **5**, **6**, **7**, **R-830-Cb**, **S-2474-Cb**, **KME-4-Cb**, and **E-5110-Cb**. Additional references are cited within the Supplementary Materials [98,99,100,101,102].

## Abbreviations

**Abbreviation**	**Meaning**
A375	Human melanoma cell line
A549	Human lung carcinoma cell line
AA	Arachidonic acid
AnnV	Annexin V
AO	Acridine orange
Apostat	FITC-conjugated pan-caspase inhibitor
ATR-IR	Attenuated total reflection infrared spectroscopy
b/br	Broad
BWA4C	*N*-[(*E*)-3-(3-Phenoxyphenyl)prop-2-enyl]acetohydroxamic acid
CDCl_3_	Deuterated chloroform
CFSE	Carboxyfluorescein *N*-succinimidyl ester
CIs	Confidence intervals
COX	Cyclooxygenase
COXIBs	COX-2 specific inhibitors
CV	Crystal violet
CYP	Cytochrome P_450_
d	Doublet
dd	Doublet of doublets
ddd	Doublet of doublets of doublets
dtd	Doublet of triplets of doublets
DHR 123	Dihydrorhodamine 123
DMAP	4-Dimethylaminopyridine
FLAP	5-Lipoxygenase-activating protein
FT-ICR	Fourier-transform ion cyclotron resonance mass spectrometry
GI	Gastrointestinal
HCT116	Human colorectal carcinoma cell line
5-H(p)ETE	5-Hydroperoxyeicosatetraenoic acid
HPLC	High-performance liquid chromatography
HR-ESI-MS	High-resolution electrospray ionization mass spectrometry
HT29	Human colorectal adenocarcinoma cell line
IC_50_	Half-maximal inhibitory concentration
log*D*	Partition coefficient
LO	Lipoxygenase
LTs	Leukotrienes
LTB_4_	Leukotriene B_4_
m	Medium (IR), multiplet (NMR), *meta*
M	Molarity
3-MA	3-Methyladenine
MDA-MB-231	M. D. Anderson-Metastatic breast-231 triple negative adenocarcinoma cell line
m.p.	Melting point
MTT	3-(4,5-Dimethylthiazol-2-yl)-2,5-diphenyl-2*H*-tetrazolium bromide
*n*BuLi	*n*-Butyllithium
NEt_3_	Triethylamine
n.i.	no inhibition
NMR	Nuclear magnetic resonance
NSAIDs	Non-steroidal anti-inflammatory drugs
*p*	*para*
PBS	Phosphate-buffered saline
PFA	Paraformaldehyde
PGs	Prostaglandins
PGE_2_	Prostaglandin E_2_
PGI_2_	Prostaglandin I_2_
PI	Propidium iodide
PMNL	Polymorphonuclear leukocytes
ppm	Parts per million
*p*-Ts	*p*-Toluenesulfonyl
q	Quartet
*R*_F_	Retention factor
RT	Room temperature
s	Strong (IR), singlet (NMR)
SC-560	5-(4-Chlorophenyl)-1-(4-methoxyphenyl)-3-trifluoromethyl pyrazole
SD	Standard deviation
SI	Selectivity index
t	Triplet
td	Triplet of doublets
TBAF	Tetra-*n*-butylammonium fluoride
TBDMSCl	*Tert*-butyldimethylsilyl chloride
TLC	Thin-layer chromatography
TMS	Tetramethylsilane
*t*_R_	Retention time
TXA_2_	Thromboxane A_2_
UPLC	Ultra-performance liquid chromatography
w	Weak
