# Supplementary material for: Synthesis and In Vitro Biological Evaluation of p-Carborane-Based Di-tert-butylphenol Analogs
Source: Molecules. 2023 Jun 4;28(11):4547. doi: 10.3390/molecules28114547 (PMC10254233; doi:10.3390/molecules28114547)
Supplement: Supplementary file 1 [file molecules-28-04547-s001.zip › molecules-2427769-supplementary.pdf]

Supplementary Materials

Synthesis and In Vitro Biological Evaluation of *p*-Carborane-Based Di-*tert*-butylphenol Analogs

Sebastian Braun[1], Sanja Jelača[2], Markus Laube[3], Sven George[4], Bettina Hofmann[4], Peter Lönnecke[1], Dieter Steinhilber[4], Jens Pietzsch[3,5], Sanja Mijatović[2], Danijela Maksimović-Ivanić[2], and Evamarie Hey-Hawkins*[1]

Table of Contents

Table of Contents 2
1. General methods, materials, and procedures 4
1.1. Biological methods 4
1.1.1. Reagents and cells 4
1.1.2. Annexin V (AnnV)/propidium iodide (PI), apostat and acridine orange (AO) staining 4
1.1.3. Measurement of reactive oxygen and nitrogen species (ROS/RNS) generation 5
1.1.4. PI staining on chamber slides 5
1.1.5. Evaluation of the role of detected autophagy 5
1.1.6. Cell proliferation assays 5
2. Chemical structures of intermediate compounds and selected spectra of analogs **R-830-Cb**, **KME-4-Cb**, **E-5110-Cb**, and **S-2474-Cb** 6
2.1. 1-(*tert*-Butyl-dimethylsiloxy)-12-(thiophen-2'-carbonyl)-1,12-dicarba-*closo*-dodecaborane(12) (**1**)...6
2.2. 1-Hydroxy-12-(thiophen-2'-carbonyl)-1,12-dicarba-*closo*-dodecaborane(12) (**R-830-Cb**) 6
2.2.1. Selected spectra of compound **R-830-Cb** 7
2.3. 1-(*tert*-Butyl-dimethylsiloxy)-12-formyl-1,12-dicarba-*closo*-dodecaborane(12) (**2**) 10
2.4. 1-(*tert*-Butyl-dimethylsiloxy)-12-(hydroxymethyl-[2'-ethylisothiazolidine-1',1'-dioxide])-1,12-dicarba-*closo*-dodecaborane(12) (**3**) 10
2.5. 1-(*tert*-Butyl-dimethylsiloxy)-12-(*p*-toluenesulfonylmethyl-[2'-ethylisothiazolidine-1',1'-dioxide])-1,12-dicarba-*closo*-dodecaborane(12) (**4**) 10
2.6. (3*E*)-1-(*tert*-Butyl-dimethylsiloxy)-12-(methylene-[2'-ethylisothiazolidine-1',1'-dioxide])-1,12-dicarba-*closo*-dodecaborane(12) (**5**) 11
2.7. (3*E*)-1-Hydroxy-12-(methylene-[2'-ethylisothiazolidine-1',1'-dioxide])-1,12-dicarba-*closo*-dodecaborane(12) (**S-2474-Cb**) 11
2.7.1. Selected spectra of compound **S-2474-Cb** 12
2.8. (3*E*)-1-(*tert*-Butyl-dimethylsiloxy)-12-(methylene-[dihydrofurane-2'(3H)-one])-1,12-dicarba-*closo*-dodecaborane(12) (**6**) 14
2.9. (3*E*)-1-(*tert*-Butyl-dimethylsiloxy)-12-(methylene-[dihydrofurane-2'(3H)-one])-1,12-dicarba-*closo*-dodecaborane(12) (**KME-4-Cb**) 15
2.9.1. Selected spectra of compound **KME-4-Cb** 15
2.10. (3*E*)-1-(*tert*-Butyl-dimethylsiloxy)-12-(methylene-[1'-methoxypyrrolidine-2'-one])-1,12-dicarba-*closo*-dodecaborane(12) (**7**) 18
2.11. (3*E*)-1-Hydroxy-12-(methylene-[1'-methoxypyrrolidine-2'-one])-1,12-dicarba-*closo*-dodecaborane(12) (**E-5110-Cb**) 18
2.11.1. Selected spectra of compound **KME-4-Cb** 19
3. Determination of purity by HPLC measurements 22
4. NMR spectroscopic stability studies 25
5. Determination of lipophilicity (log*D*) by HPLC 26
6. Biological evaluation 26
6.1. Intact PMNL whole-cell assay (5-LO) 26
7. Single Crystal X-ray diffraction data 31
7.1. 1-(*tert*-Butyl-dimethylsiloxy-12-(thiophen-2'-carbonyl)-1,12-dicarba-*closo*-decaborane(12) (**1**) 32
7.2. 1-Hydroxy-12-(thiophen-2'-carbonyl)-1,12-dicarba-*closo*-decaborane(12) (**R-830-Cb**) 33
7.3. (3*E*)-1-(*tert*-Butyl-dimethylsiloxy)-12-(methylene-[2'-ethylisothiazolidine-1',1'-dioxide])-1,12-dicarba-*closo*-decaborane(12) (**5**) 35
7.4. (3*E*)-1-Hydroxy-12-(methylene-[2'-ethylisothiazolidine-1',1'-dioxide])-1,12-dicarba-*closo*-decaborane(12) (**S-2474-Cb**) 36
7.5. (3*E*)-1-(*tert*-Butyl-dimethylsiloxy)-12-(methylene-[dihydrofurane-2'(3*H*)-one])-1,12-dicarba-*closo*-decaborane(12) (**6**) 38
7.6. (3*E*)-1-Hydroxy-12-(methylene-[dihydrofurane-2'(3*H*)-one])-1,12-dicarba-*closo*-decaborane(12) (**KME-4-Cb**) 39
7.7. (3*E*)-1-(*tert*-Butyl-dimethylsiloxy)-12-(methylene-[1'-methoxypyrrolidine-2'-one])-1,12-dicarba-*closo*-decaborane(12) (**7**) 41

7.8. (3*E*)-1-Hydroxy-12-(methylene-[1’-methoxypyrrolidine-2’-one])-1,12-dicarba-*closo*-decaborane(12) (**E-5110-Cb**) 42
8. References 44

1. General methods, materials, and procedures

1.1. Biological methods

1.1.1. Reagents and cells

Reagents and cells were obtained from following manufacturers: Capricorn Scientific GmbH (Hessen, Germany) – Culture medium RPMI-1640, Dulbecco's Modified Eagle Medium (DMEM), and fetal bovine serum (FBS). Serva (Heidelberg, Germany) – Paraformaldehyde (PFA). Sigma (St. Louis, MO, USA) – Ethylenediaminetetraacetic acid (EDTA), Dimethyl sulfoxide (DMSO), chloroquine, CV, Triton X-100, RNase, PI, PBS, 3-MA, CFSE, and fluorescent mounting medium. AppliChem (MO, USA) –MTT. Thermo Fisher Scientific (Waltham, MA, USA) – DHR. R&D Systems (Minneapolis, MN, USA) – ApoStat. BD (Pharmingen, San Diego, SAD) – Annexin V-FITC (AnnV). Biological Industries (Cromwell, CT, USA) – Penicillin Streptomycin solution. American Type Culture Collection (ATCC, Manassas, Virginia, USA) – Human lung carcinoma (A549), human breast adenocarcinoma (MDA-MB-231), human colorectal adenocarcinoma (HT-29), human colorectal carcinoma (HCT116) and human melanoma (A375).

Cell lines (A375, HCT116, HT-29 and MDA-MB-231) were cultivated in HEPES-buffered RPMI-1640 medium while A549 cell line was cultivated in Dulbecco's Modified Eagle Medium (DMEM) which were previously supplemented with 10% heat-inactivated FBS, penicillin (100 units/mL), and streptomycin (100 µg/mL). All cells were kept at 37 °C in a humidified atmosphere with 5% CO_2. For viability determination cells were seeded at following densities in 96-well plates: 4 x 10^3 cells/well (A375, HT-29 and MDA-MB-231), 5 x 10^3 cells/well (HCT116), and 3 x 10^3 cells/well (A549). The density of HCT116 cells in 6-well plates for flow cytometric analyses was 1.5 x 10^5 cells/well.

Primary peritoneal exudates cells (PEC) isolated from C57BL/6 mice were used to estimate the selectivity of compounds **R-830** and **R-830-Cb** to malignant phenotype. Mice were obtained from the animal facility at the Institute for Biological Research "Siniša Stanković" - National Institute of the Republic of Serbia, University of Belgrade (Belgrade, Serbia). PEC were cultivated in HEPES-buffered (HEPES = 4-(2-hydroxyethyl)-1-piperazineethanesulfonic acid) RPMI-1640 medium, previously supplemented with 5% heat-inactivated fetal bovine serum (FBS) and antibiotics under standard growth conditions (37 °C, 5% CO_2). For viability determination cells were seeded at a density of 2 x 10^5 cells/well in 96-well plates. Non-adherent cells were removed two hours after seeding and treatment was applied. Cell viability was determined after 72 h using a crystal violet (CV) assay.

1.1.2. Annexin V (AnnV)/propidium iodide (PI), apostat and acridine orange (AO) staining

For detection of apoptosis, HCT116 cells were exposed to an IC_{50} dose of compounds **R-830** (30 µM) and **R-830-Cb** (50 µM) for 72 h. Afterwards, cells were washed with phosphate-buffered saline (PBS) and stained with AnnV (15 µg/mL) and PI (15 µg/mL). After 15 min incubation at room temperature (RT) protected from light the cells were resuspended in AnnV-binding buffer and analysed using flow cytometry. HCT116 cells were incubated with pan-caspase inhibitor Apostat for 30 min at 37 °C to determine whether detected apoptosis was mediated by caspase activation. Afterwards, cells were washed with PBS and analyzed using flow cytometry. HCT116 cells were stained with 1 µg/mL of AO solution for 15 min at 37 °C to detect the presence of autophagic cell death. Afterwards cells were washed with PBS, resuspended and analyzed by flow cytometry (CyFlow® Space Partec using the PartecFloMax® software (Münster, Germany)).

1.1.3. Measurement of reactive oxygen and nitrogen species (ROS/RNS) generation

Dihydrorhodamine 123 (DHR) staining was used for detection of production of ROS/RNS species. HCT116 cells were prestained with 1 µM DHR for 20 min at 37 °C and treated with an IC_{50} dose of compounds **R-830** (30 µM) and **R-830-Cb** (50 µM). Finally, after 72 h incubation, cells were washed, trypsinized, and analyzed using flow cytometry (CyFlow® Space Partec using the PartecFloMax® software (Münster, Germany)).

1.1.4. PI staining on chamber slides

For evaluation of morphological signs of apoptosis HCT116 cells were seeded overnight in 4-chamber slides at density of 4×10^4 followed by treatment with an IC_{50} dose of selected compounds (**R-830** (30 µM) and **R-830-Cb** (50 µM)). After 72 h incubation, cells were washed with PBS and fixed with 4% PFA for 15 min at RT. Thereafter, cells were washed with PBS, and stained with a solution of PI in a concentration of 50 µg/mL with 0.1 mM EDTA pH 8.0, 0.1% Triton X-100 and RNase (85 µg/mL) in PBS for 1 min. Finally, for covering the slides fluorescent mounting medium was used and slides were analyzed with Zeiss AxioObserver Z1 inverted fluorescence microscope (Carl Zeiss AG, Oberkochen, Germany) at 400 x magnification.

1.1.5. Evaluation of the role of detected autophagy

Concomitant treatment of **R-830-Cb** and autophagy inhibitors chloroquine and 3-MA was performed to determine the nature of detected autophagy. HCT116 cells were exposed to IC_{50} dose of **R-830-Cb** (50×10^{-6} M) and 20×10^{-6} M concentration of chloroquine and 1×10^{-3} M concentration of 3-MA concomitantly. Cell viability was evaluated using CV assay after 72 h.

1.1.6. Cell proliferation assays (CFSE staining)

CFSE staining was used for detection of cell proliferation. Prior to seeding, HCT116 cells were stained with CFSE to a final concentration of 1 µM for 10 min at 37 °C, washed, seeded, and treated with an IC_{50} dose of selected compounds (**R-830** (30 µM) and **R-830-Cb** (50 µM)). Finally, after 72 h incubation, cells were tripsynized, washed, resuspended in PBS, and analyzed by flow cytometry (CyFlow® Space Partec using the PartecFloMax® software (Munster, Germany)).

2. Chemical structures of intermediate compounds and selected spectra of analogs **R-830-Cb**, **KME-4-Cb**, **E-5110-Cb**, and **S-2474-Cb**

2.1. 1-(*tert*-Butyl-dimethylsiloxy)-12-(thiophen-2’-carbonyl)-1,12-dicarba-*closo*-dodecaborane(12) (**1**)

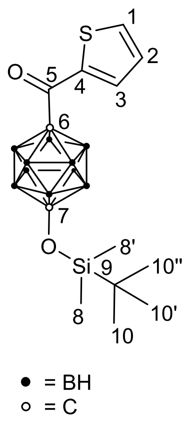

1

2.2. 1-Hydroxy-12-(thiophen-2’-carbonyl)-1,12-dicarba-*closo*-dodecaborane(12) (**R-830-Cb**)

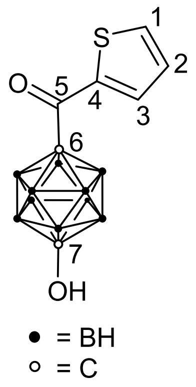

R-830-Cb

2.2.1. Selected spectra of compound **R-830-Cb**

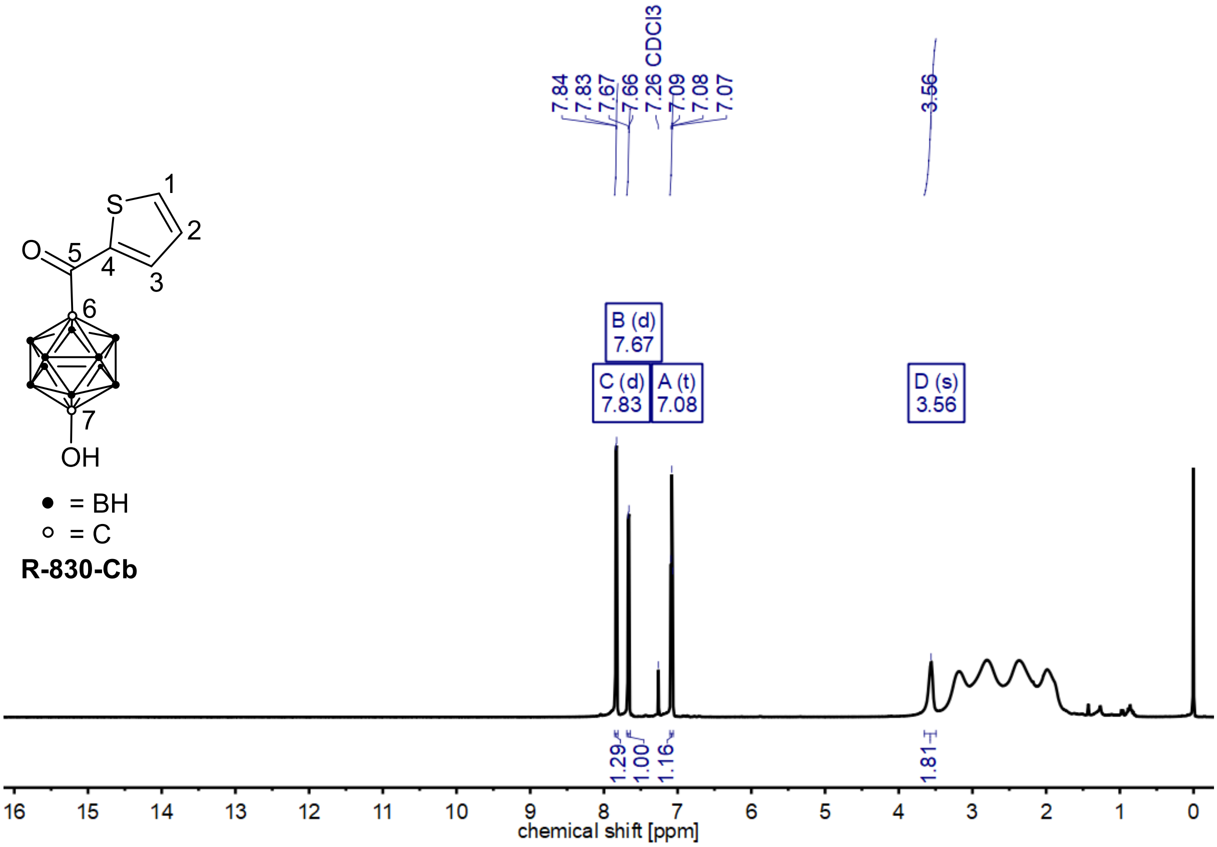

Figure S1. ^{1}H-NMR spectrum of compound **R-830-Cb** in deuterated chloroform.

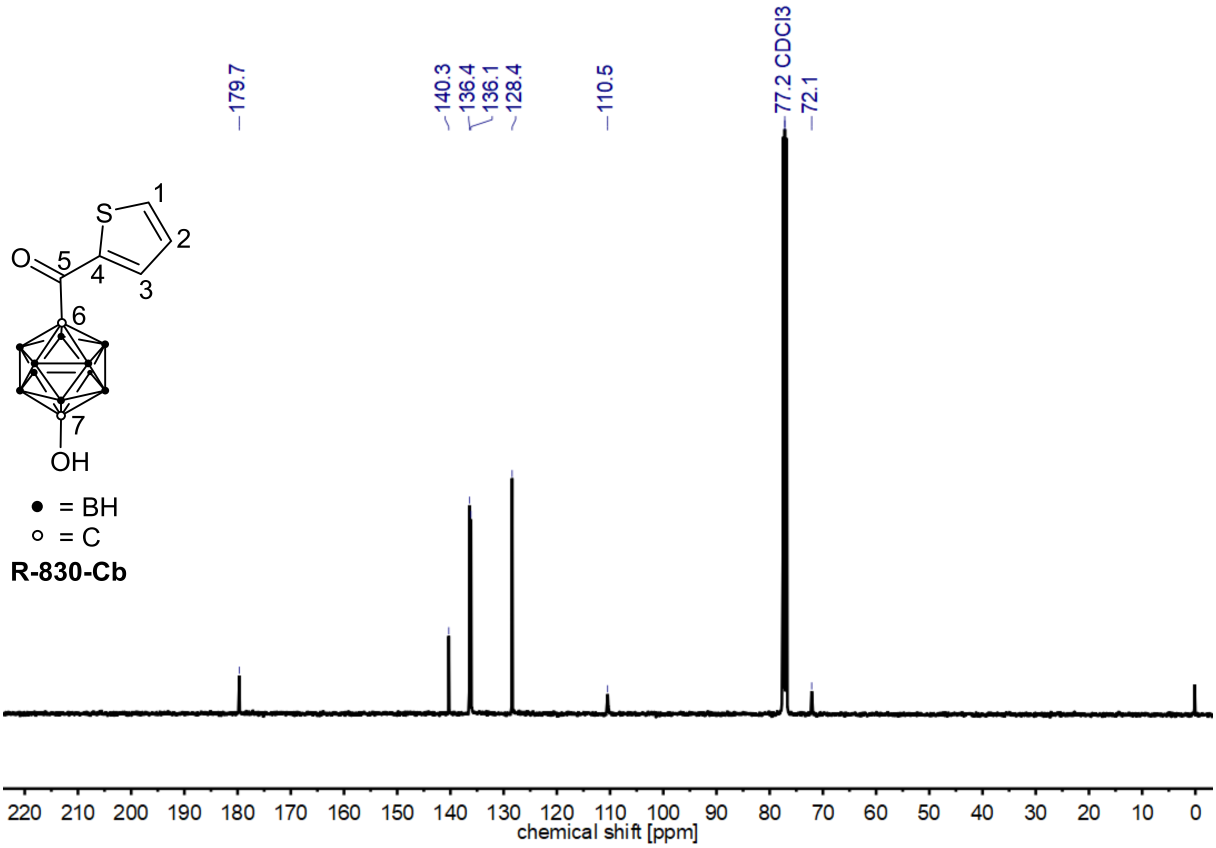

Figure S2. ^{13}C{^{1}H}-NMR spectrum of compound **R-830-Cb** in deuterated chloroform.

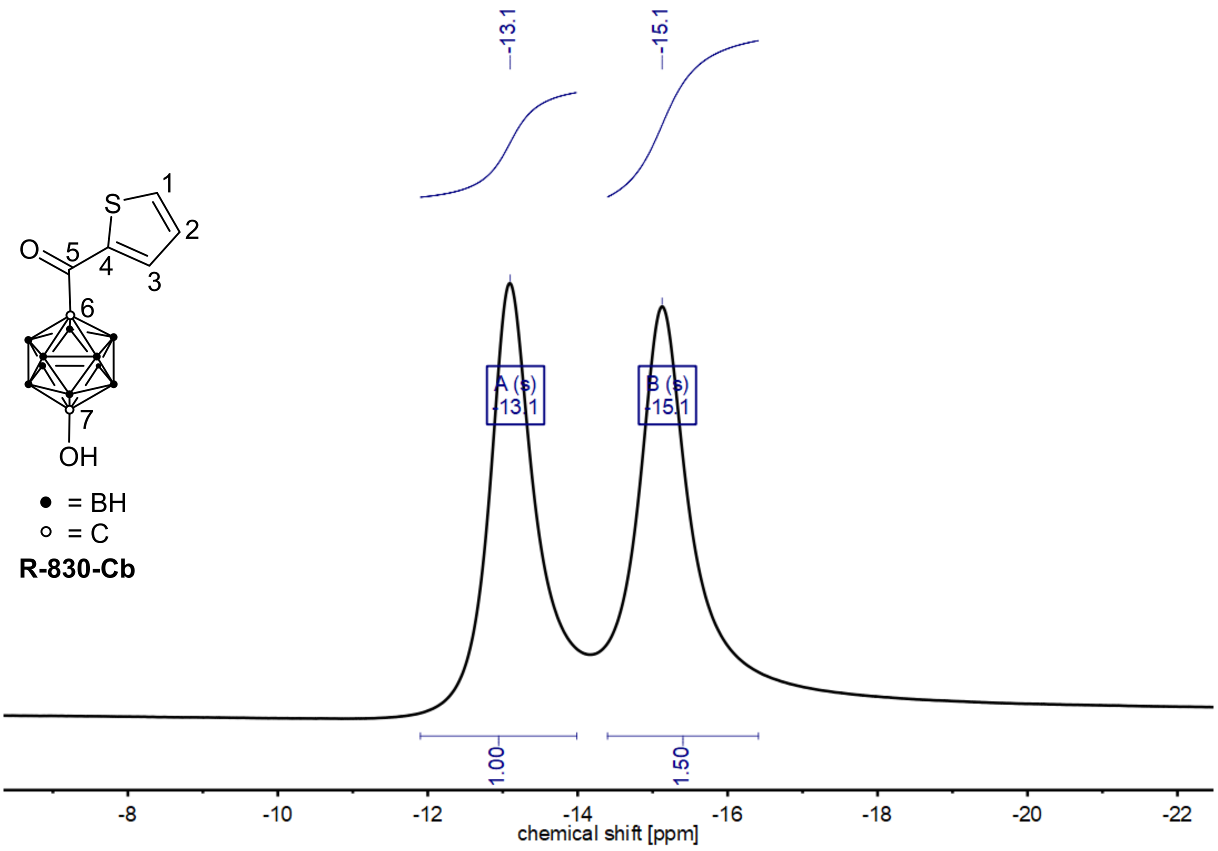

Figure S3. $^{11}B\{^{1}H\}$-NMR spectrum of compound **R-830-Cb** in deuterated chloroform.

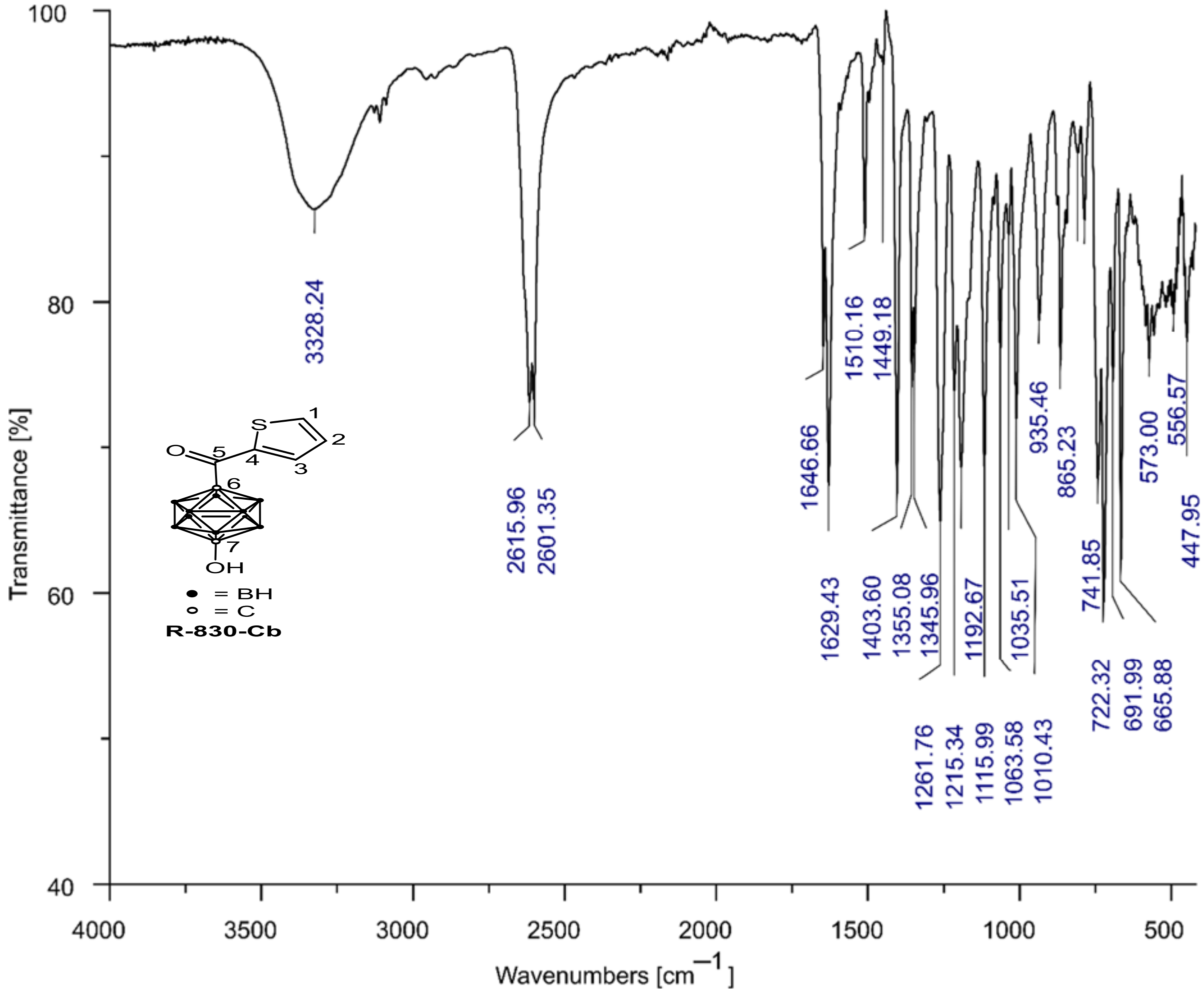

Figure S4. FT-IR spectrum of compound **R-830-Cb**.

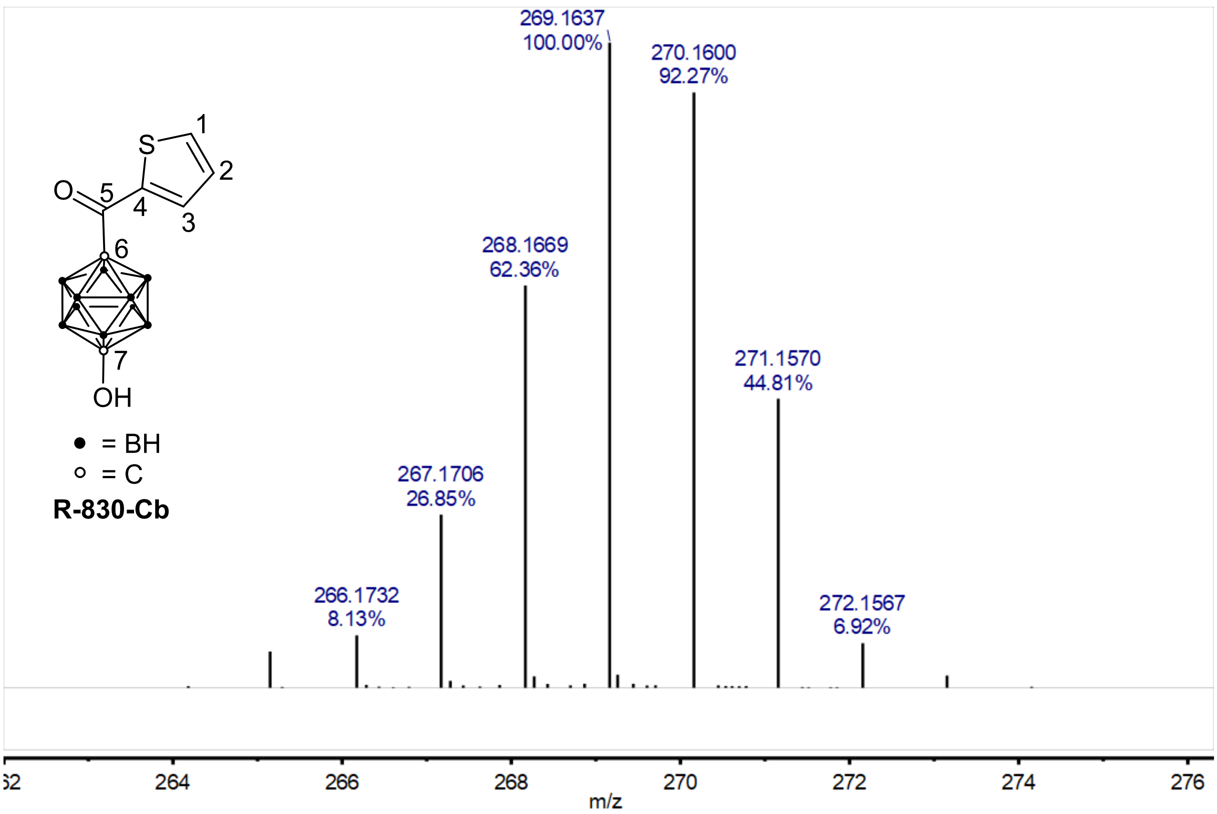

Figure S5. Selected isotopic pattern of HR-ESI-MS spectrum of compound **R-830-Cb** in methanol.

2.3. 1-(*tert*-Butyl-dimethylsiloxy)-12-formyl-1,12-dicarba-*closo*-dodecaborane(12**)** (**2**)

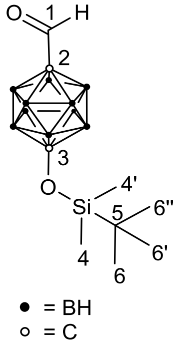

2

2.4. 1-(*tert*-Butyl-dimethylsiloxy)-12-(hydroxymethyl-[2’-ethylisothiazolidine-1’,1’-dioxide])-1,12-dicarba-*closo*-dodecaborane(12) (**3**)

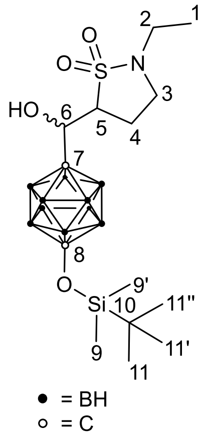

3

2.5. 1-(*tert*-Butyl-dimethylsiloxy)-12-(*p*-toluenesulfonylmethyl-[2’-ethylisothiazolidine-1’,1’-dioxide])-1,12-dicarba-*closo*-dodecaborane(12) (**4**)

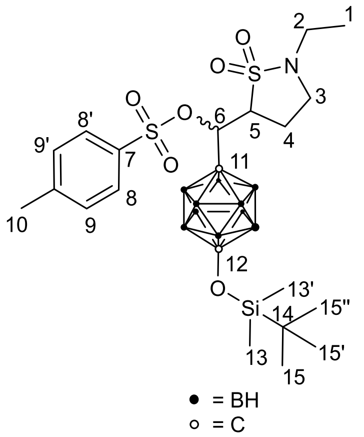

4

2.6. (3*E*)-1-(*tert*-Butyl-dimethylsiloxy)-12-(methylene-[2’-ethylisothiazolidine-1’,1’-dioxide])-1,12-dicarba-*closo*-dodecaborane(12) (**5**)

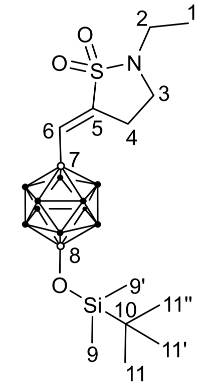

• = BH
○ = C

5

2.7. **(**3*E*)-1-Hydroxy-12-(methylene-[2’-ethylisothiazolidine-1’,1’-dioxide])-1,12-dicarba-*closo*-dodecaborane(12) (**S-2474-Cb**)

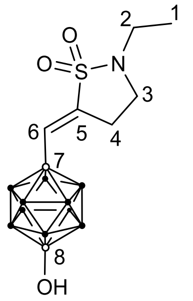

• = BH
○ = C

S-2474-Cb

2.7.1. Selected spectra of compound **S-2474-Cb**

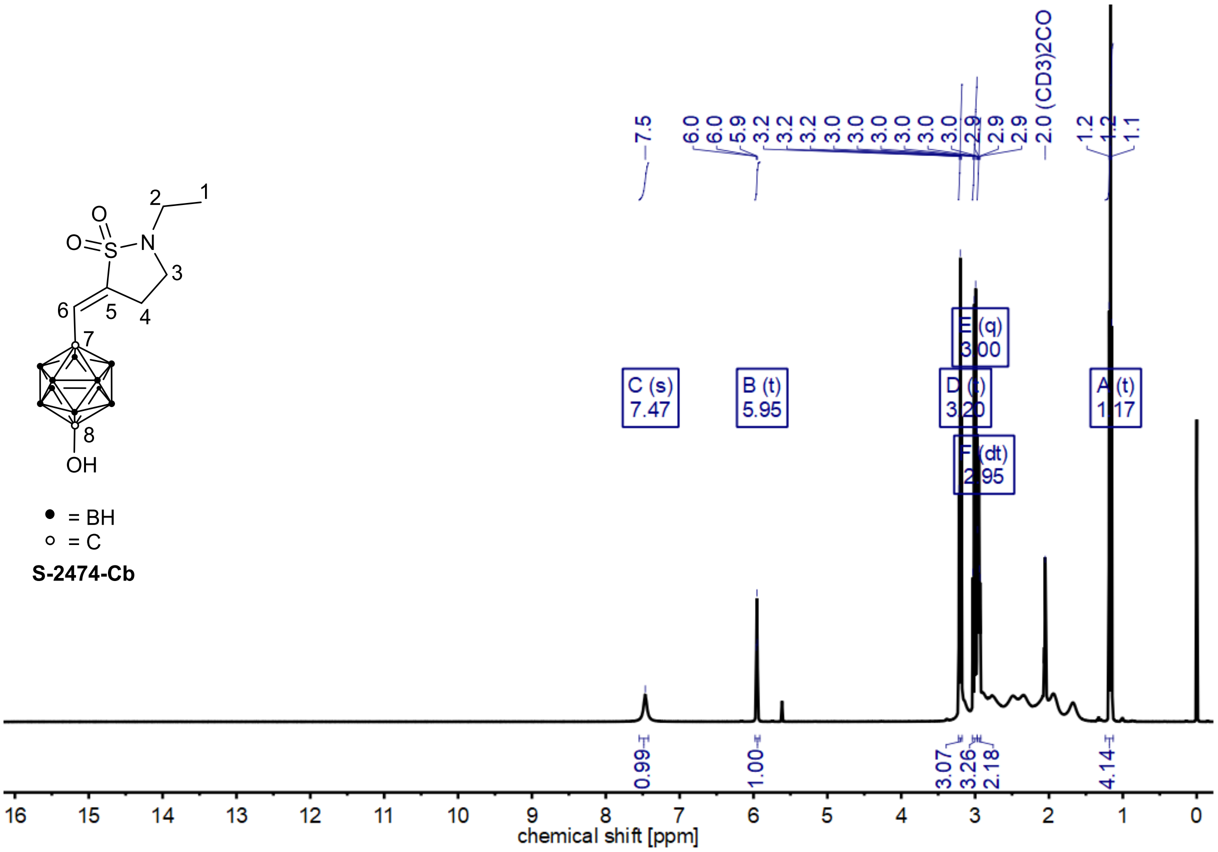

Figure S6. ^{1}H-NMR spectrum of compound **S-2474-Cb** in deuterated acetone.

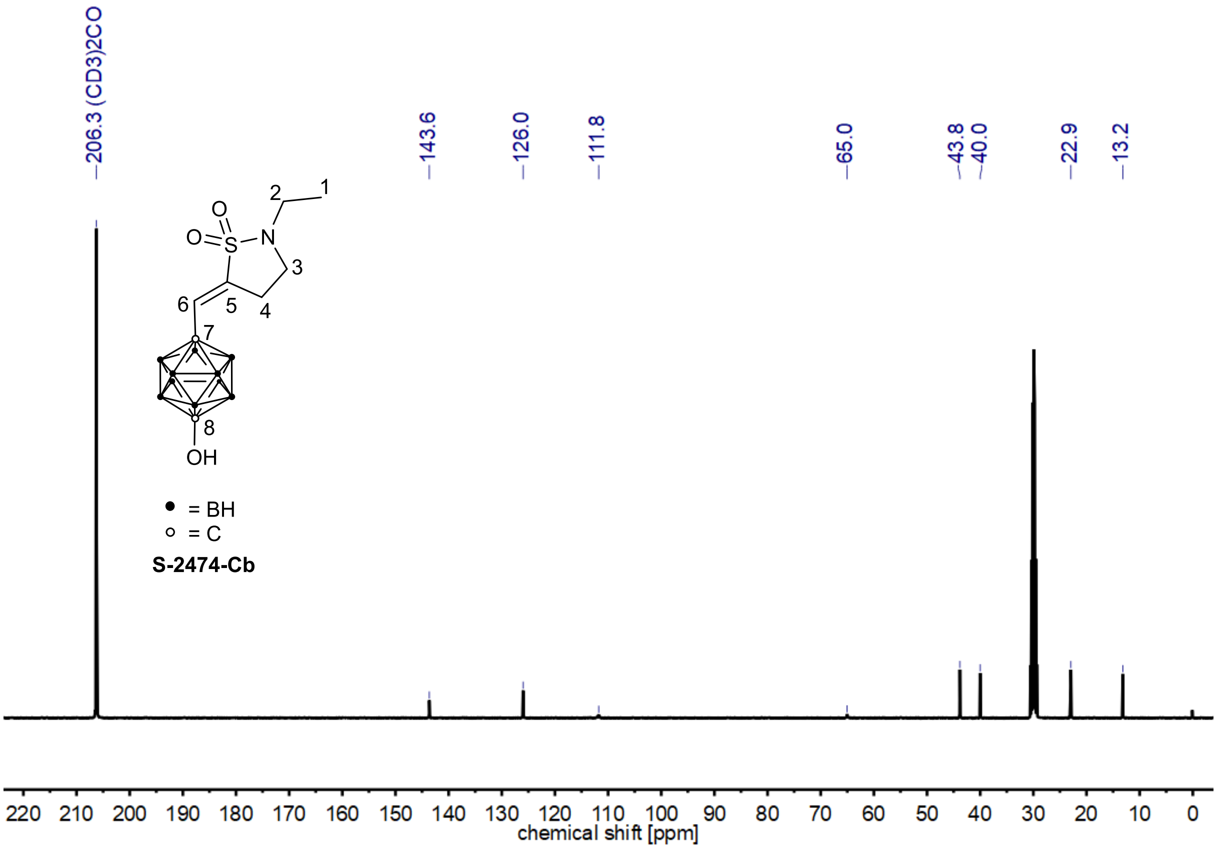

Figure S7. $^{13}C\{^{1}H\}$-NMR spectrum of compound **S-2474-Cb** in deuterated acetone.

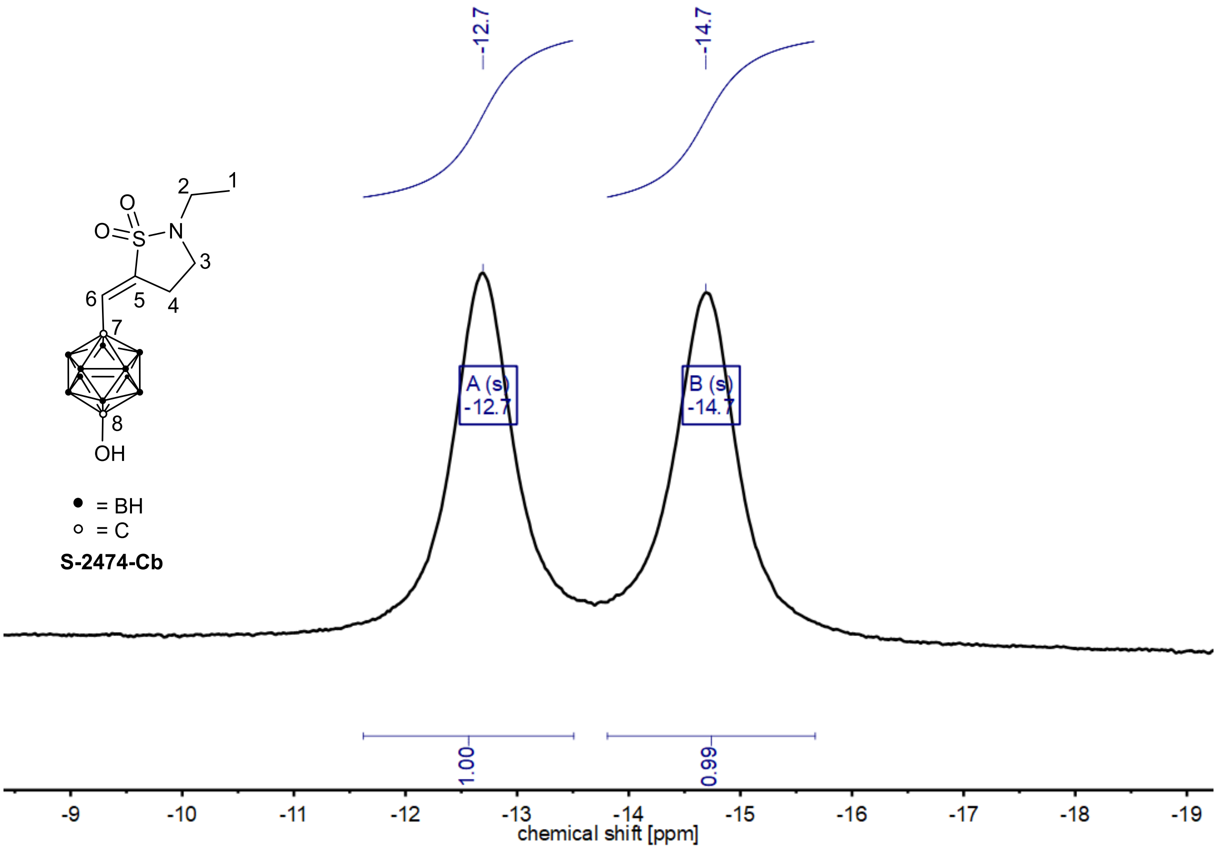

Figure S8. $^{11}B\{^{1}H\}$-NMR spectrum of compound **S-2474-Cb** in deuterated acetone.

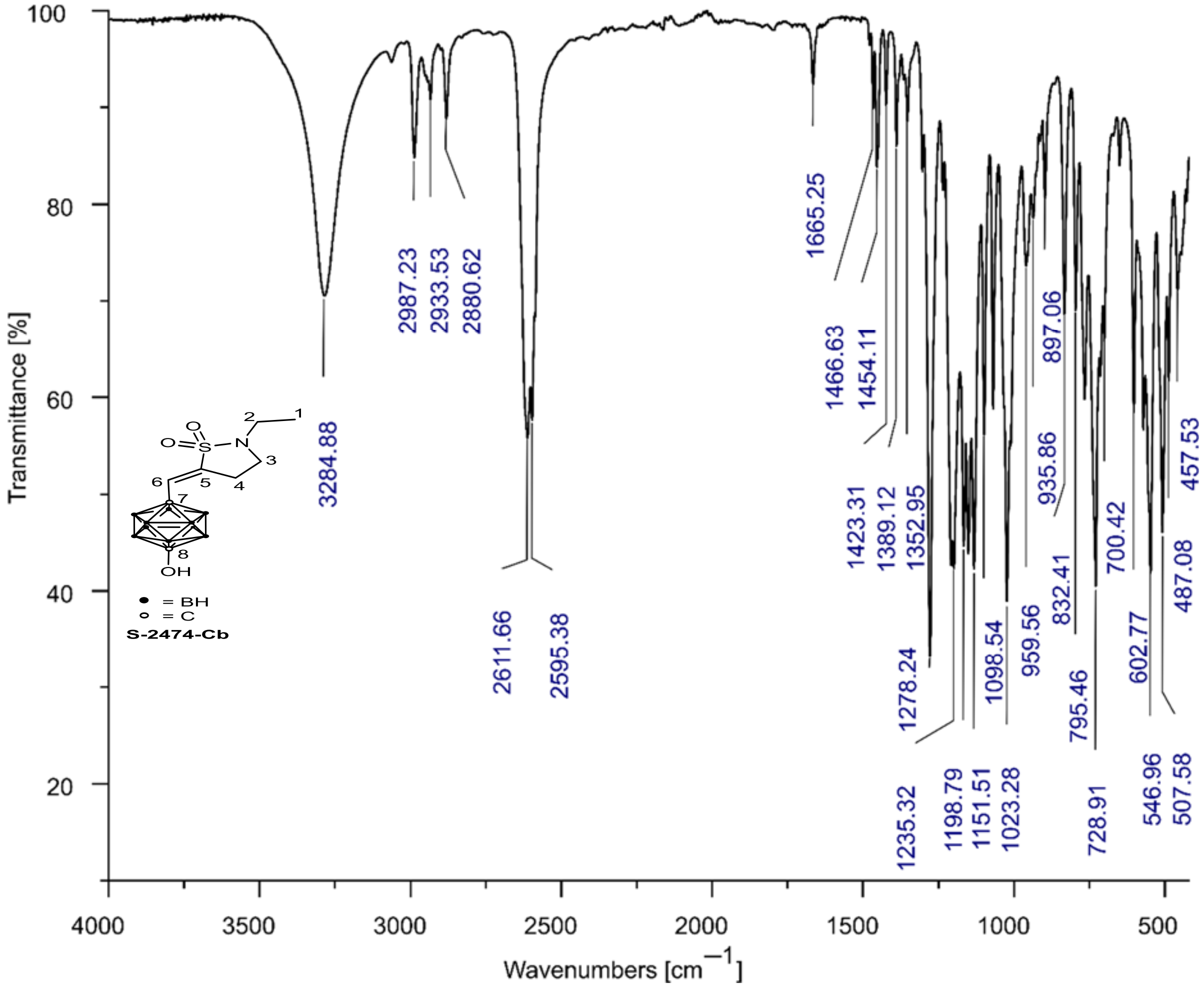

Figure S9. FT-IR spectrum of compound **S-2474-Cb**.

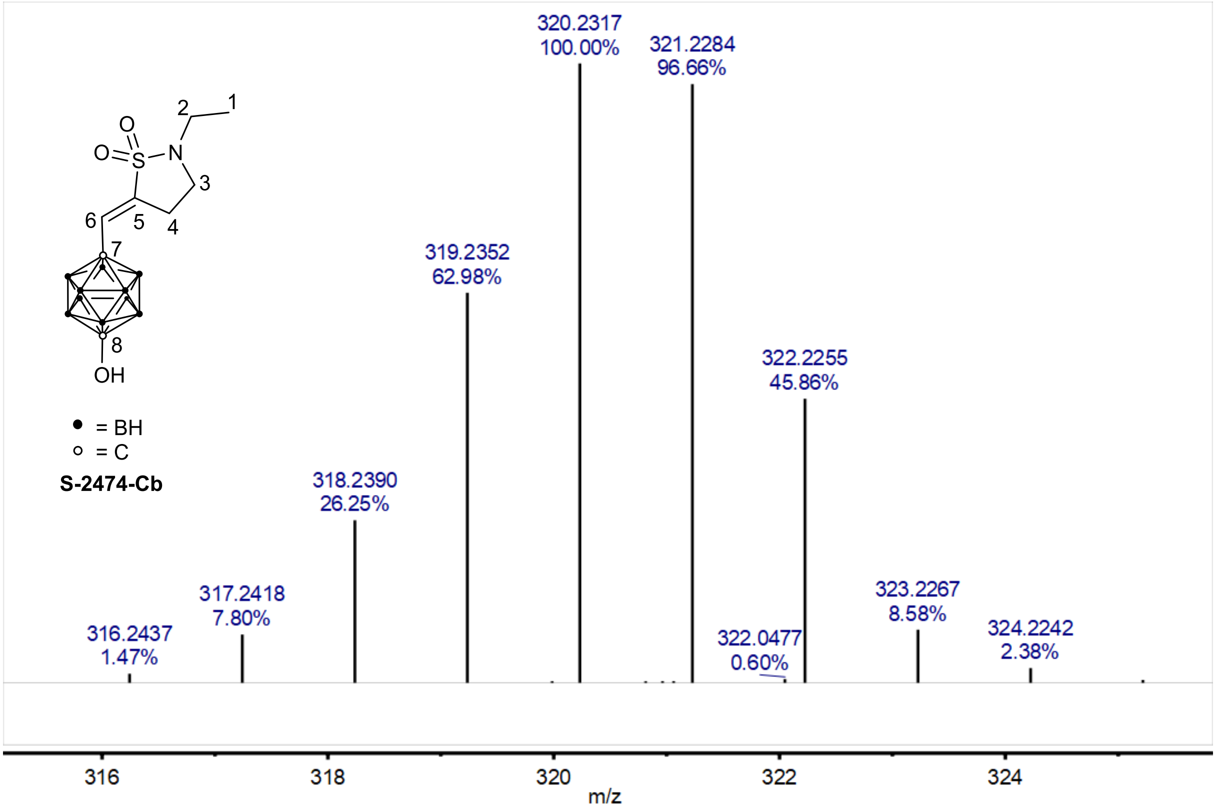

Figure S10. Selected isotopic pattern of HR-ESI-MS spectrum of compound **S-2474-Cb** in methanol.

2.8. **(**3*E*)-1-(*tert*-Butyl-dimethylsiloxy)-12-(methylene-[dihydrofurane-2'(3*H*)-one])-1,12-dicarba-*closo*-dodecaborane(12) (**6**)

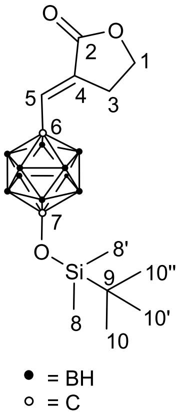

6

2.9. (3*E*)-1-(*tert*-Butyl-dimethylsiloxy)-12-(methylene-[dihydrofurane-2'(3*H*)-one])-1,12-dicarba-*closo*-dodecaborane(12) (**KME-4-Cb**)

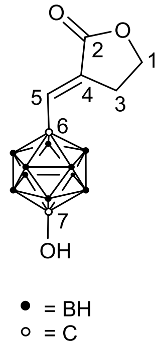

KME-4-Cb

2.9.1. Selected spectra of compound **KME-4-Cb**

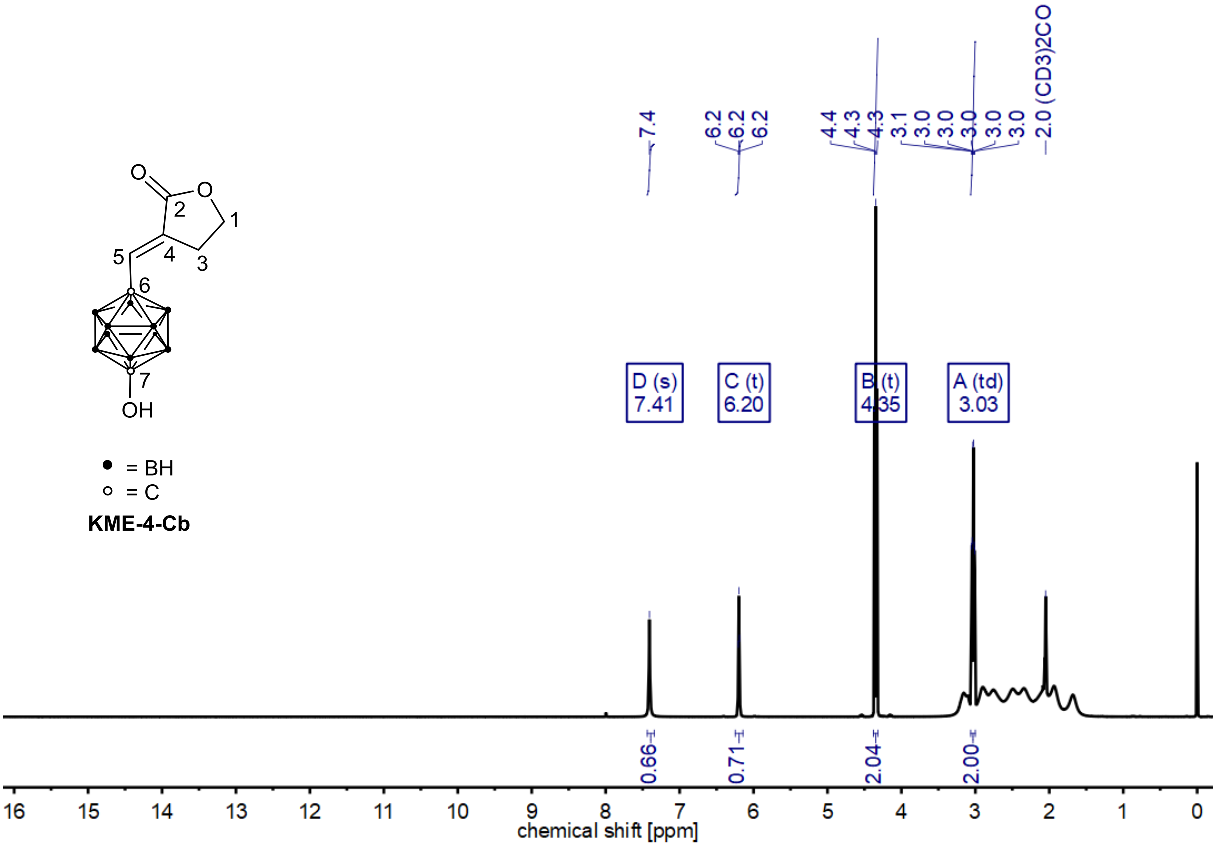

Figure S11. ^{1}H-NMR spectrum of compound **KME-4-Cb** in deuterated acetone.

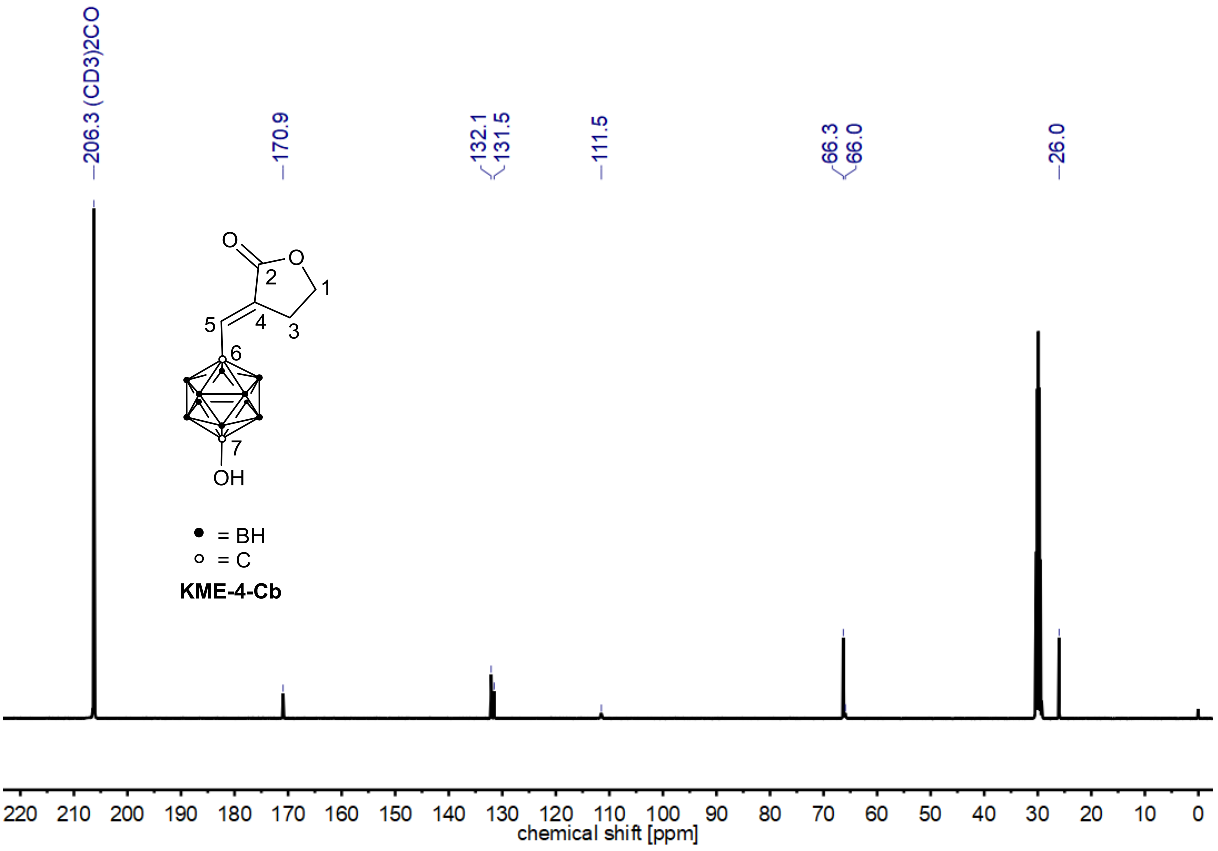

Figure S12. $^{13}C\{^{1}H\}$-NMR spectrum of compound **KME-4-Cb** in deuterated acetone.

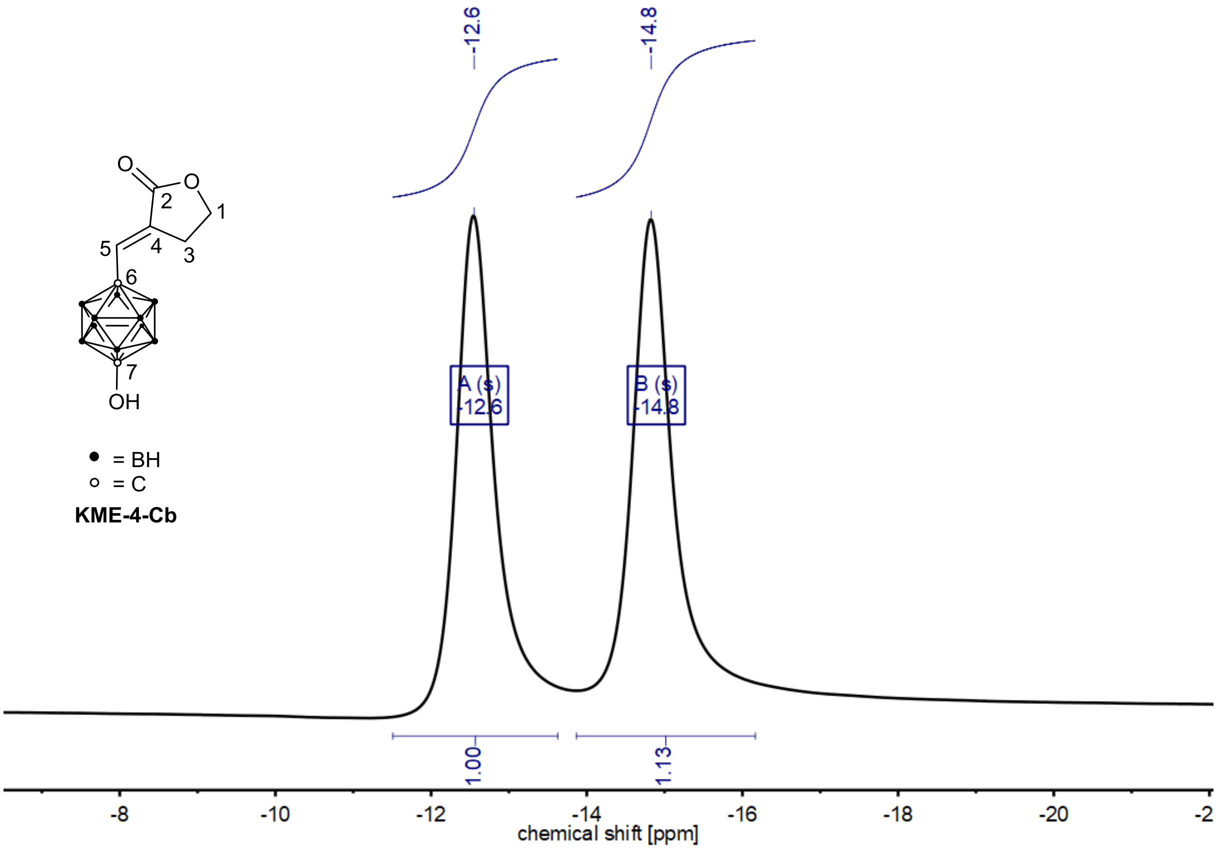

Figure S13. $^{11}B\{^{1}H\}$-NMR spectrum of compound **KME-4-Cb** in deuterated acetone.

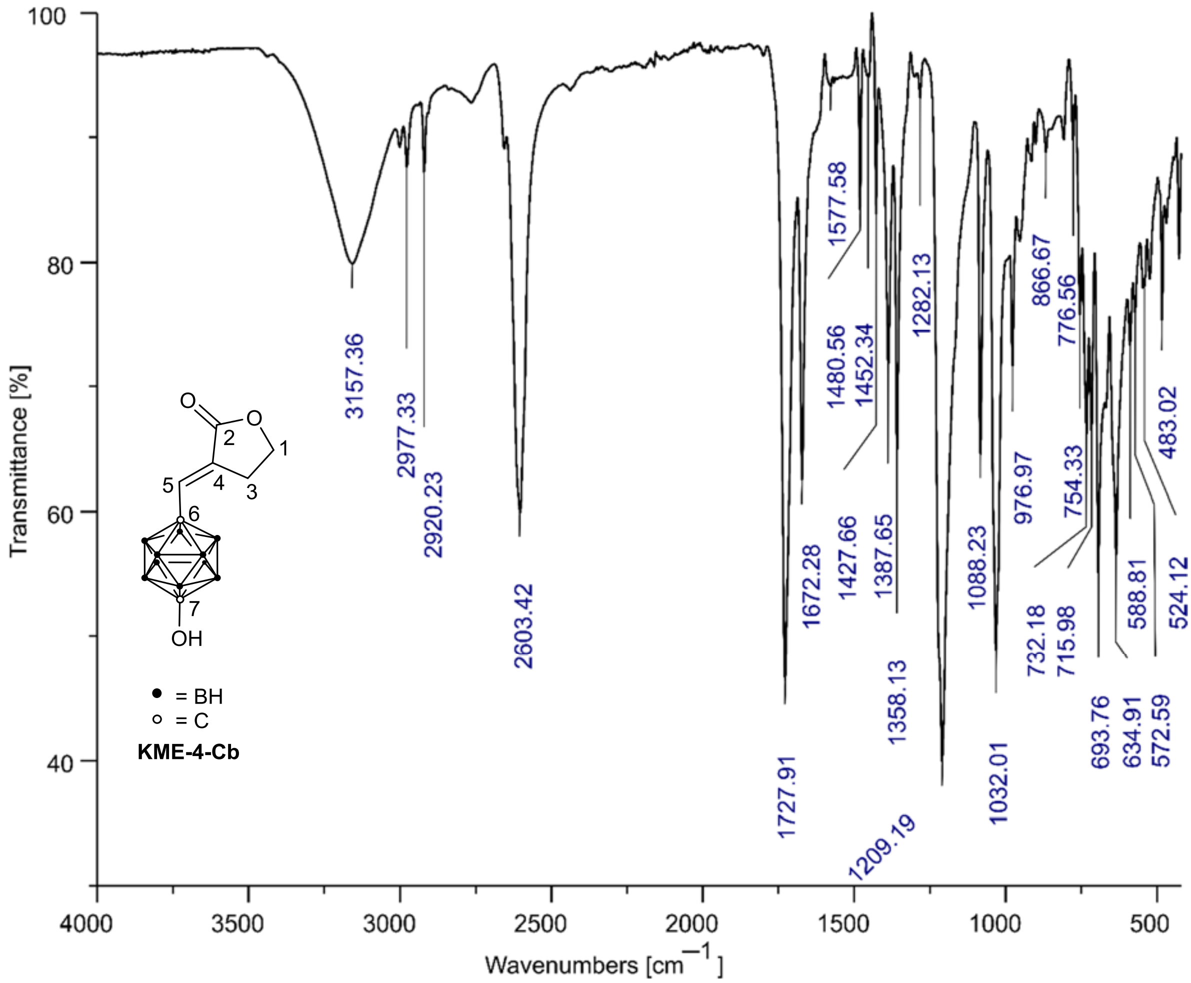

Figure S14. FT-IR spectrum of compound **KME-4-Cb.**

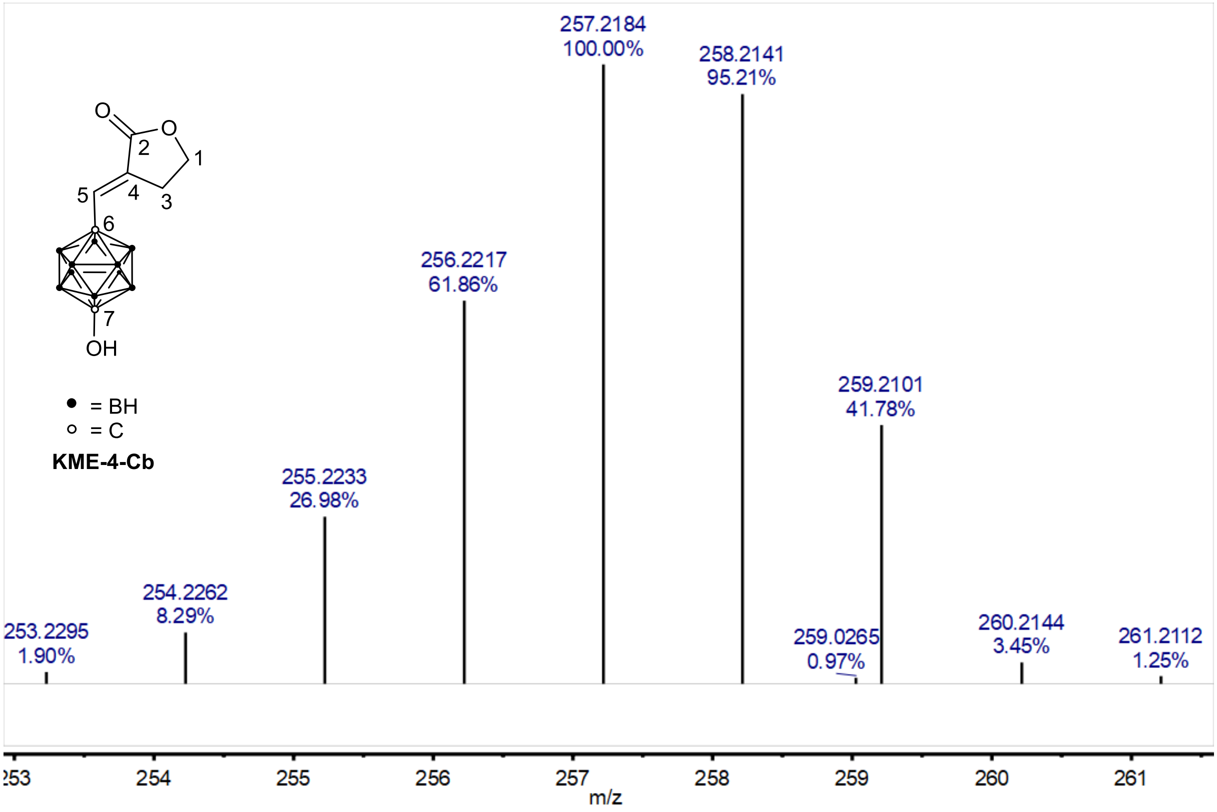

Figure S15. Selected isotopic pattern of HR-ESI-MS spectrum of compound **KME-4-Cb** in acetonitrile.

2.10. (3*E*)-1-(*tert*-Butyl-dimethylsiloxy)-12-(methylene-[1’-methoxypyrrolidine-2’-one])-1,12-dicarba-*closo*-dodecaborane(12) (**7**)

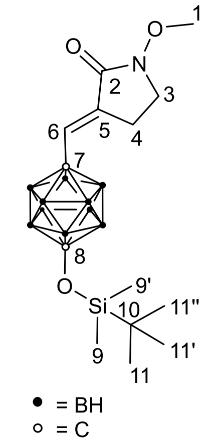

7

2.11. (3*E*)-1-Hydroxy-12-(methylene-[1’-methoxypyrrolidine-2’-one])-1,12-dicarba-*closo*-dodecaborane(12) (**E-5110-Cb**)

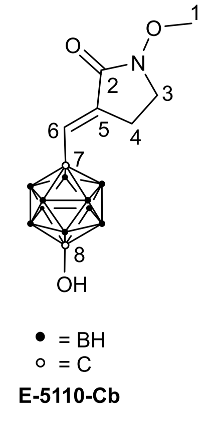

E-5110-Cb

2.11.1. Selected spectra of compound **KME-4-Cb**

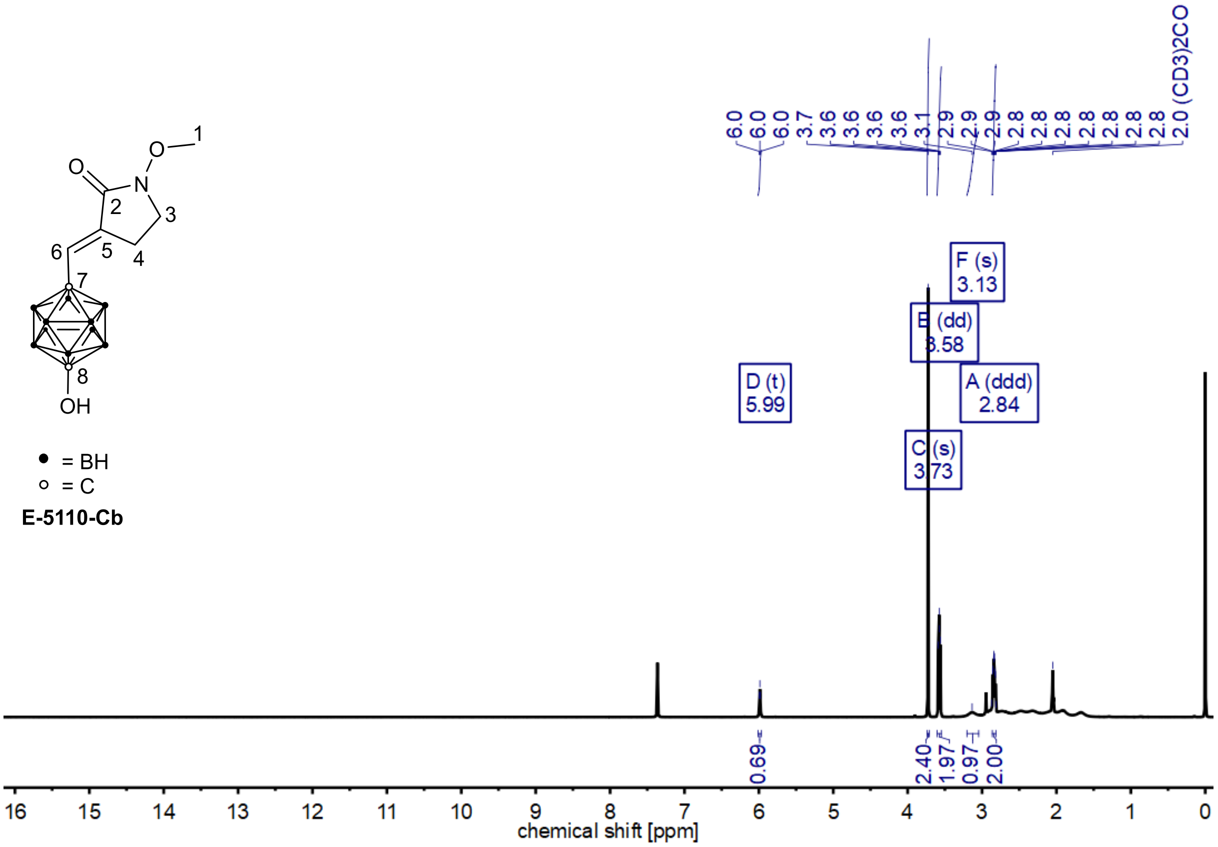

Figure S16. ^{1}H-NMR spectrum of compound **E-5110-Cb** in deuterated acetone.

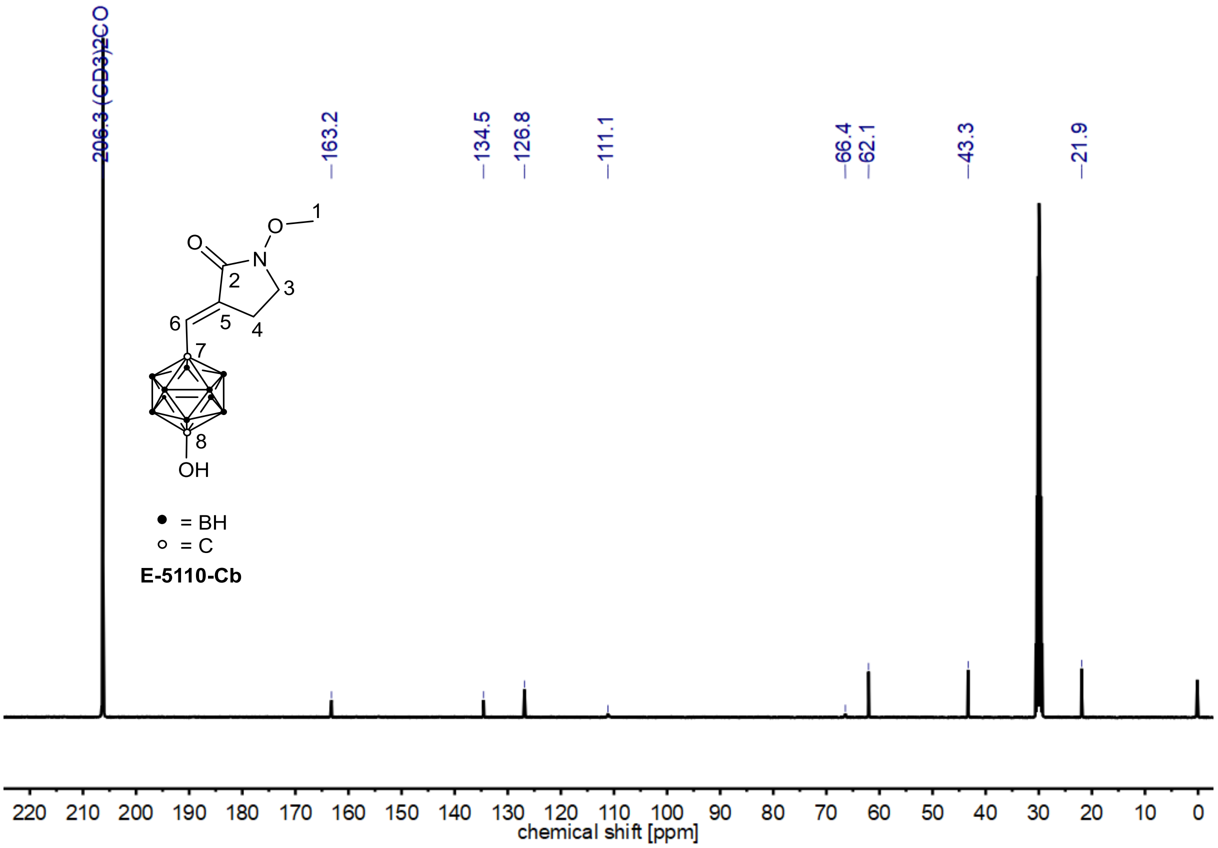

Figure S17. ^{13}C{^{1}H}-NMR spectrum of compound **E-5110-Cb** in deuterated acetone.

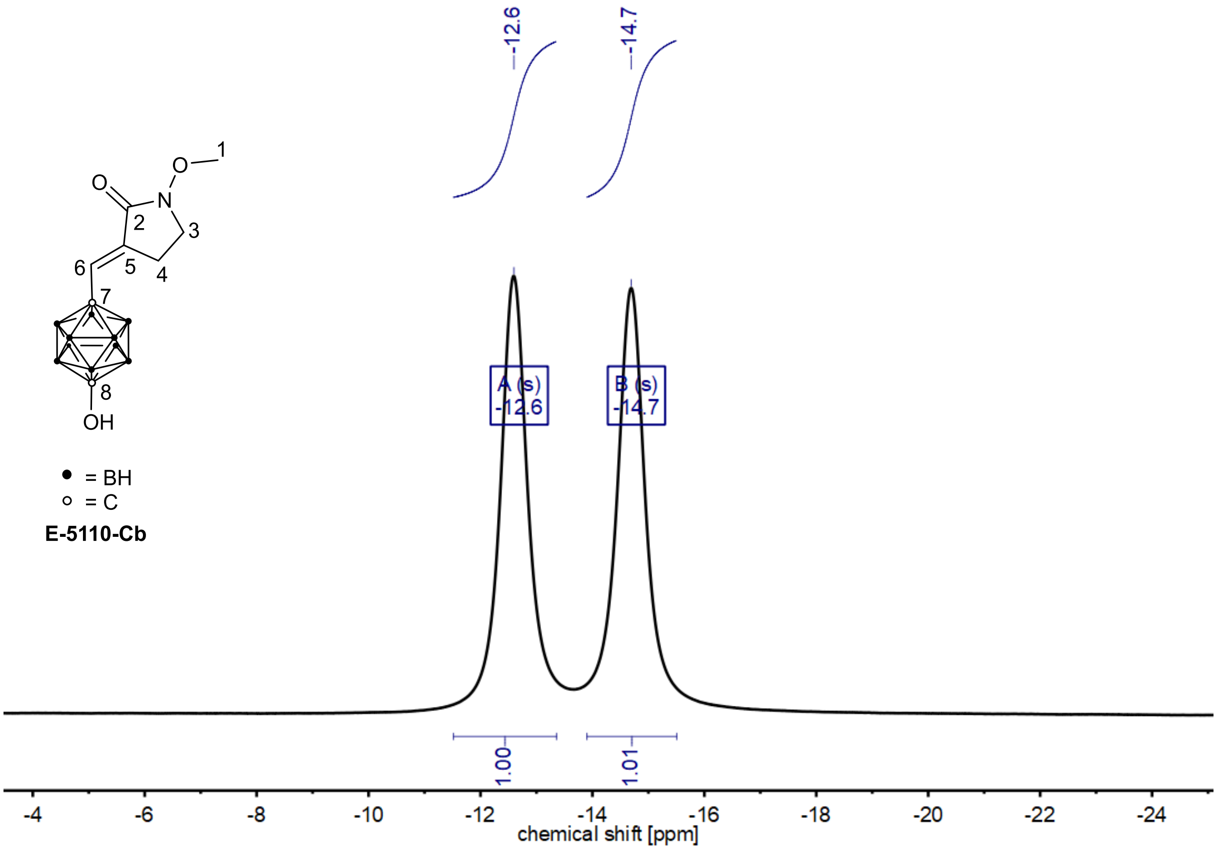

Figure S18. $^{11}B\{^{1}H\}$-NMR spectrum of compound **E-5110-Cb** in deuterated acetone.

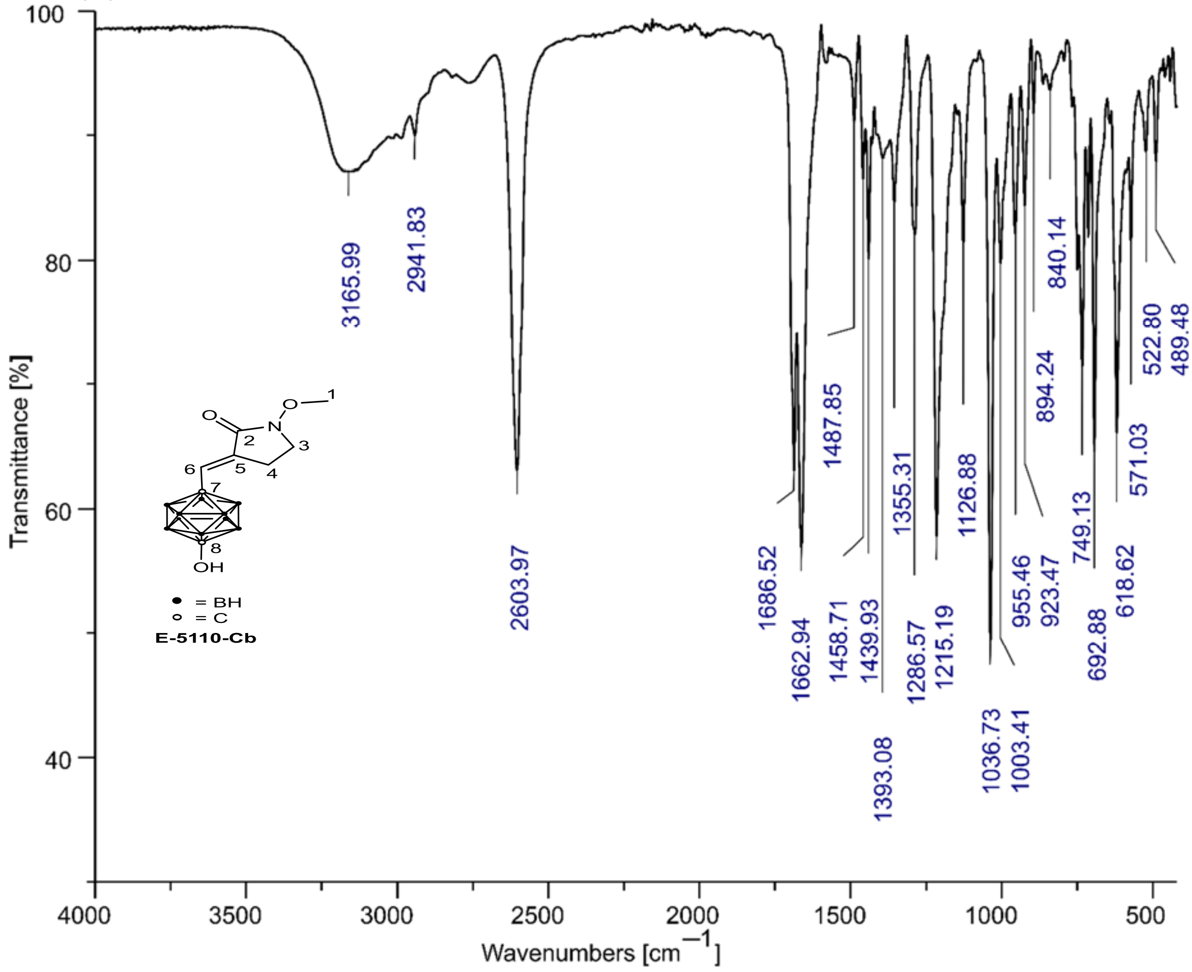

Figure S19. FT-IR spectrum of compound **E-5110-Cb**.

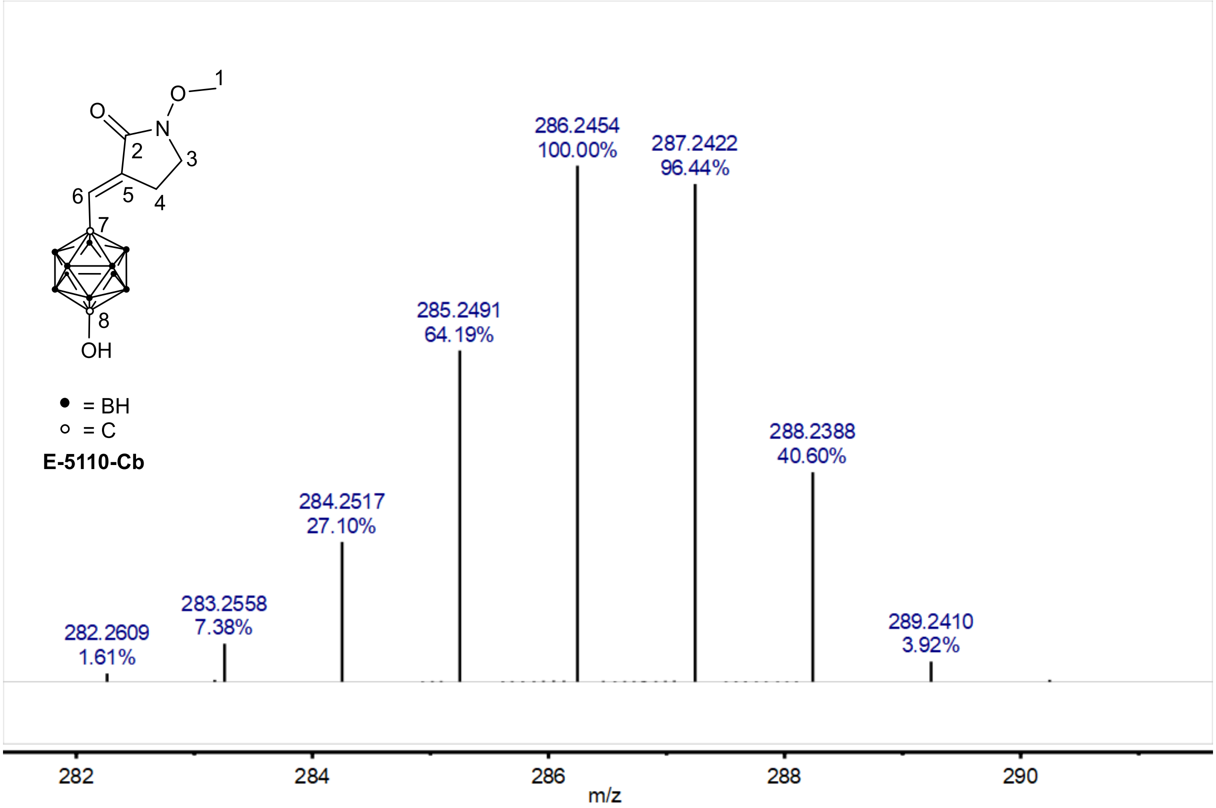

Figure S20. Selected isotopic pattern of HR-ESI-MS spectrum of compound **E-5110-Cb** in methanol.

3. Determination of purity by HPLC measurements

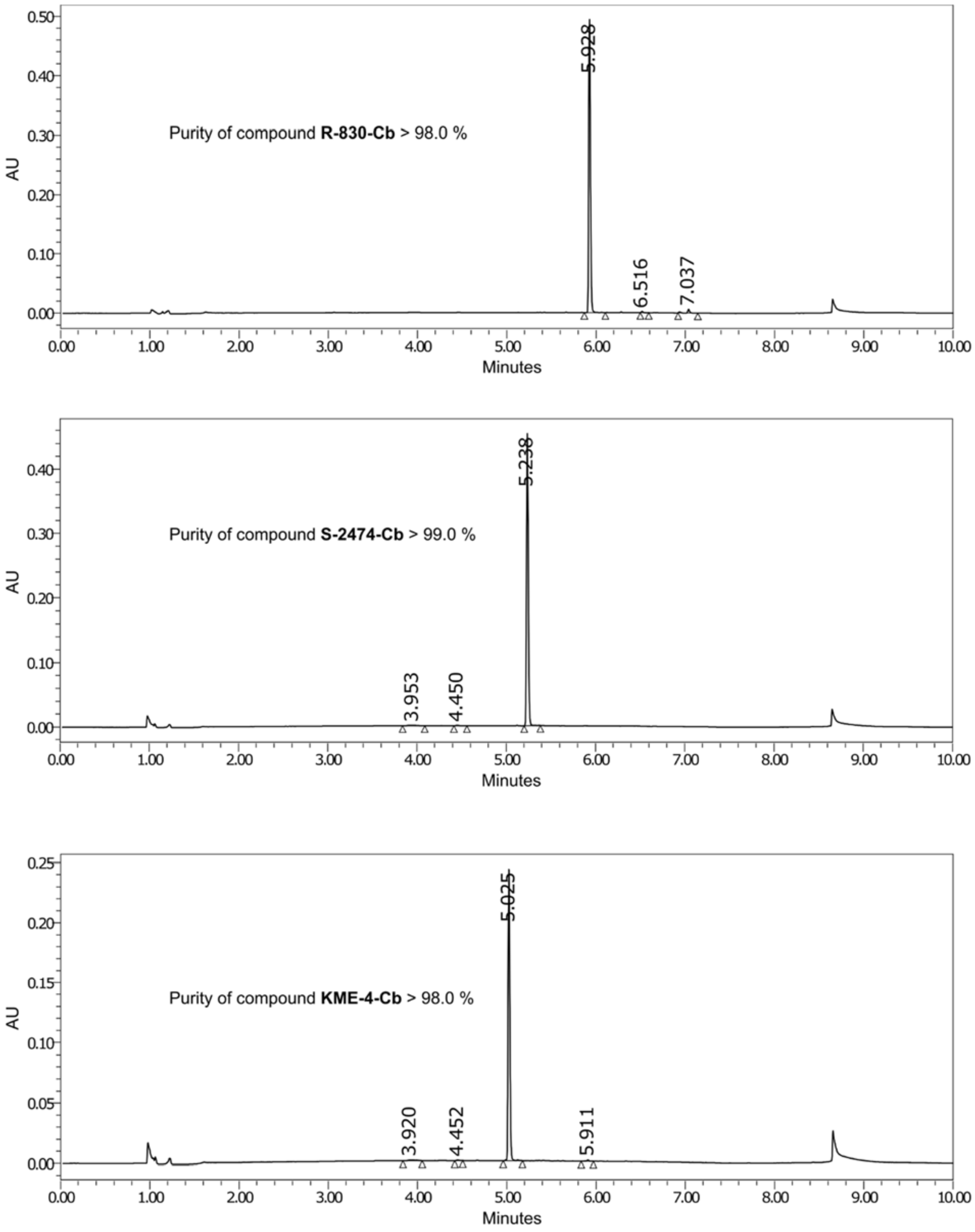

Figure S21. HPLC measurements of **R-830-Cb**, **S-2474-Cb,** and **KME-4-Cb**.

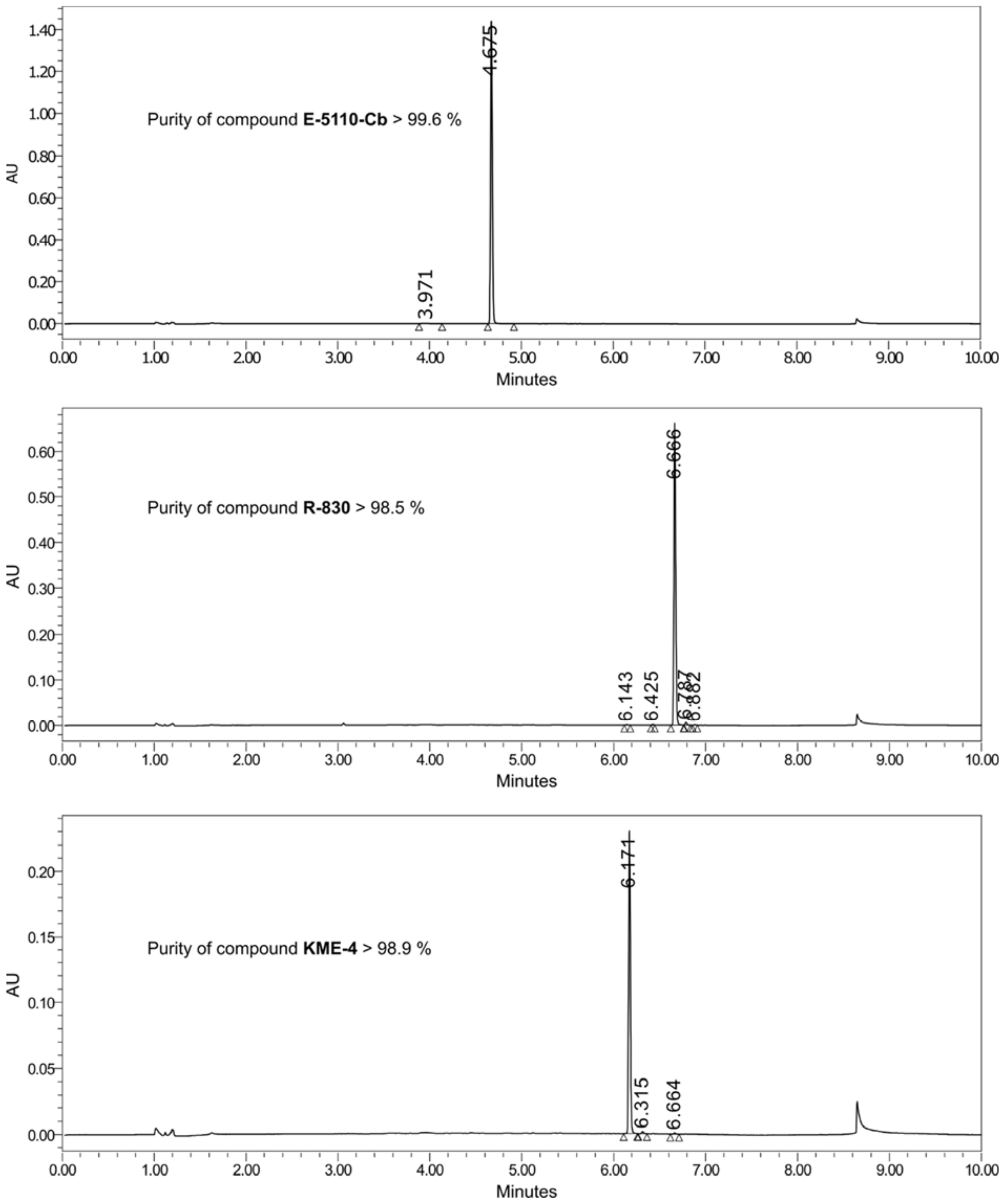

Figure S22. HPLC measurements of **E-5110-Cb**, **R-830,** and **KME-4**.

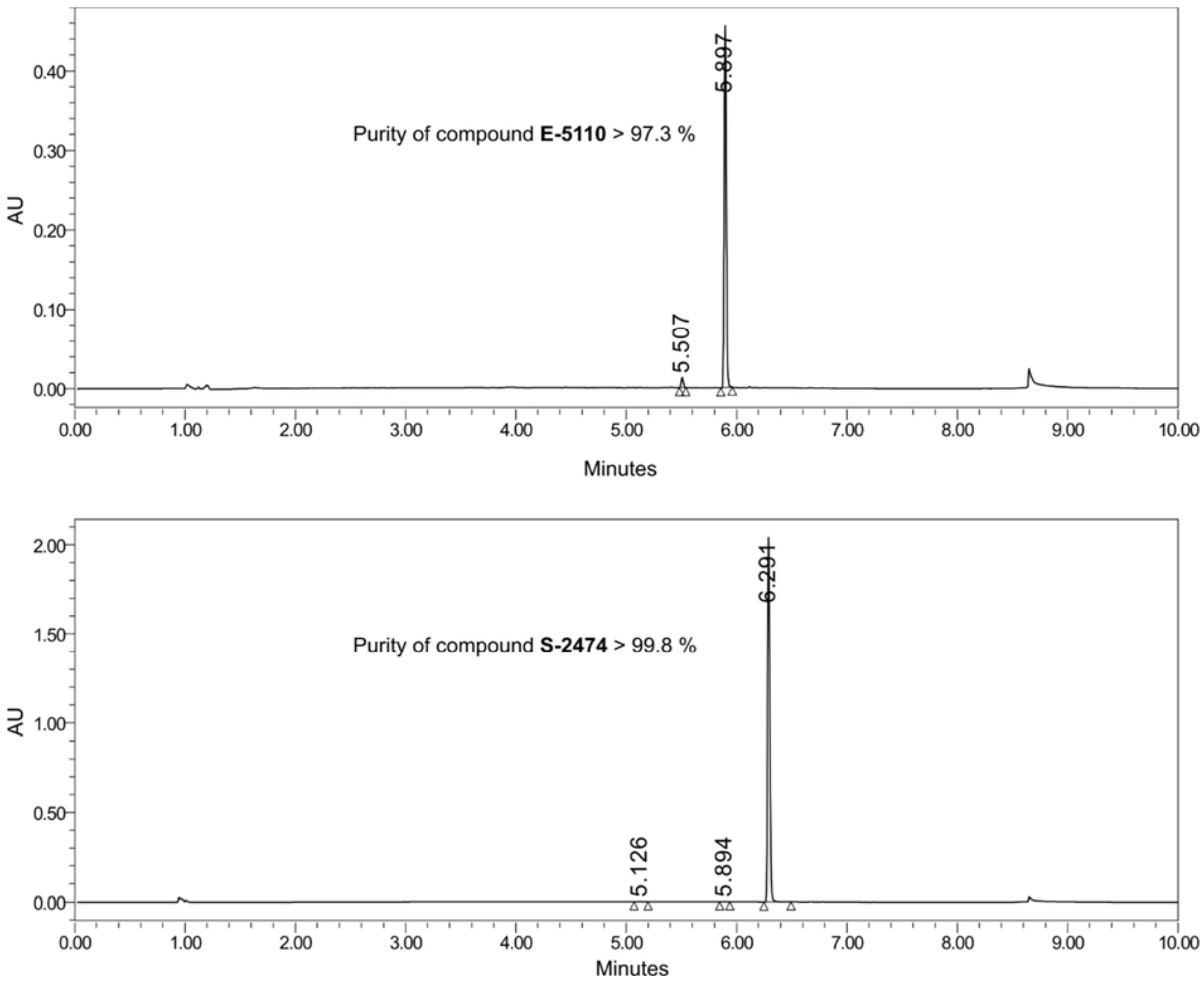

Figure S23. HPLC measurements of **E-5110** and **S-2474**.

4. NMR spectroscopic stability studies

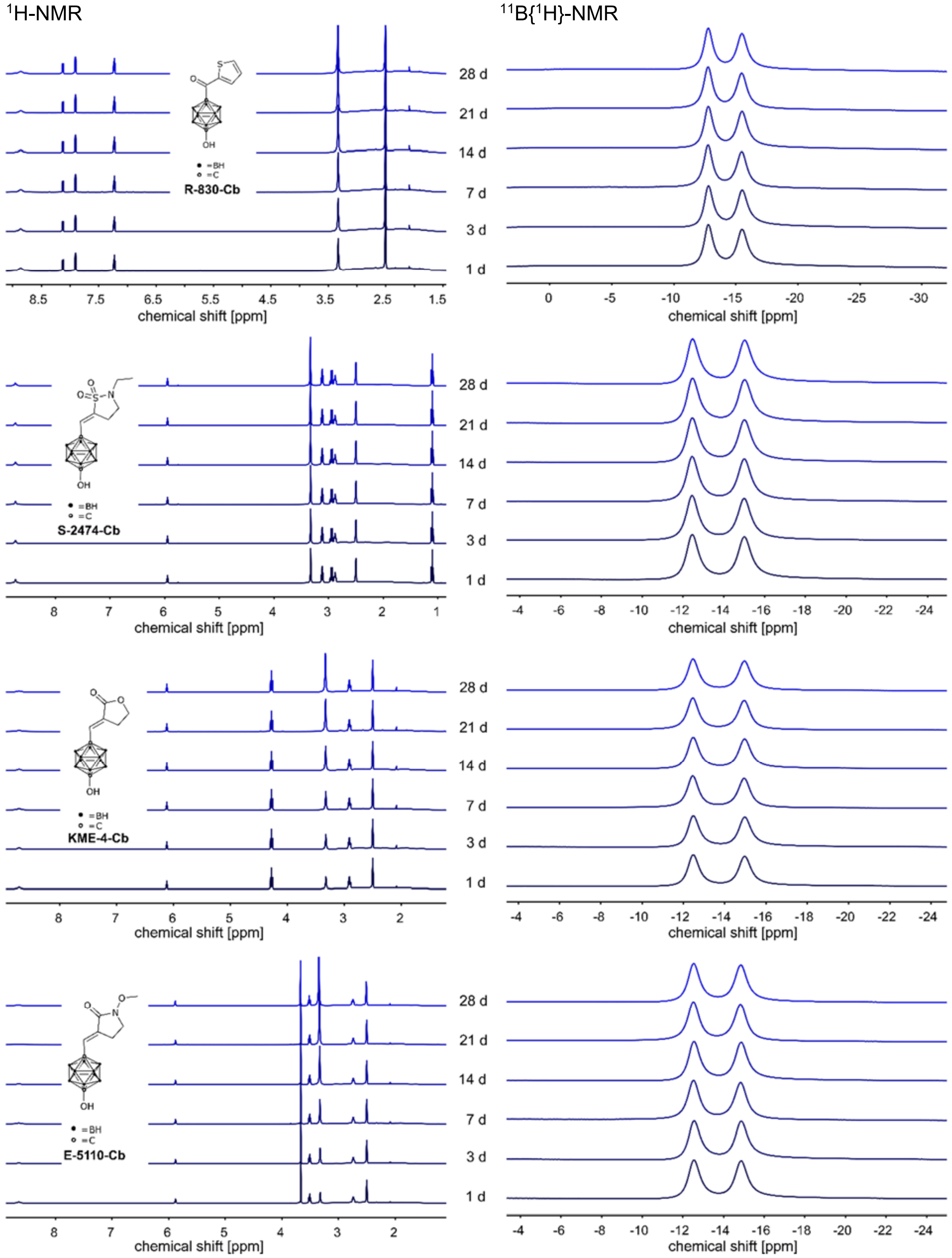

Figure S24. ^{1}H-NMR (left) and ^{11}B{^{1}H}-NMR spectra (right) of **R-830-Cb**, **S-2474-Cb**, **KME-4-Cb**, and **E-5110-Cb** (in DMSO-d_6).

5. Determination of lipophilicity (log*D*) by HPLC

Table S1. The log$D_{7.4,\ HPLC}$ values and corresponding retention times.

Compound	log$D_{7.4,\ HPLC}$ ± SD	t_R ± SD [min]
**R-830**	3.91 ± 0.02	22.07 ± 0.07
**R-830-Cb**	2.37 ± 0.01	14.86 ± 0.03
**KME-4**	3.79 ± 0.01	21.48 ± 0.03
**KME-4-Cb**	1.87 ± 0	12.48 ± 0.02
**E-5110**	3.35 ± 0.01	19.42 ± 0.04
**E-5110-Cb**	1.5 ± 0	10.74 ± 0
**S-2474**	3.75 ± 0	21.3 ± 0
**S-2474-Cb**	1.88 ± 0.02	12.55 ± 0.09

6. Biological evaluation

6.1. Intact PMNL whole-cell assay (5-LO)

Table S2. IC_{50} values for inhibition of 5-LO product formation in intact PMNL for the carborane derivatives **R-830-Cb**, **S-2474-Cb**, **KME-4-Cb**, **E-5110-Cb**, and their respective reference compounds **R-830**, **S-2474**, **KME-4**, and **E-5110**. Data are presented as mean of at least three independent experiments with 95% CIs (CIs = confidence intervals).

Compound	IC_{50} [µM] (95% CI)	Compound	IC_{50} [µM] (95% CI)
**R-830-Cb**	0.65 (0.23-1.8)	**R-830**	0.26 (0.12-0.58)
**KME-4-Cb**	0.07 (0.01-0.53)	**KME-4**	0.15 (0.08-0.27)
**E-5110-Cb**	0.22 (0.11-0.47)	**E-5110**	0.12 (0.07-0.21)
**S-2474-Cb**	< 0.03	**S-2474**	< 0.03

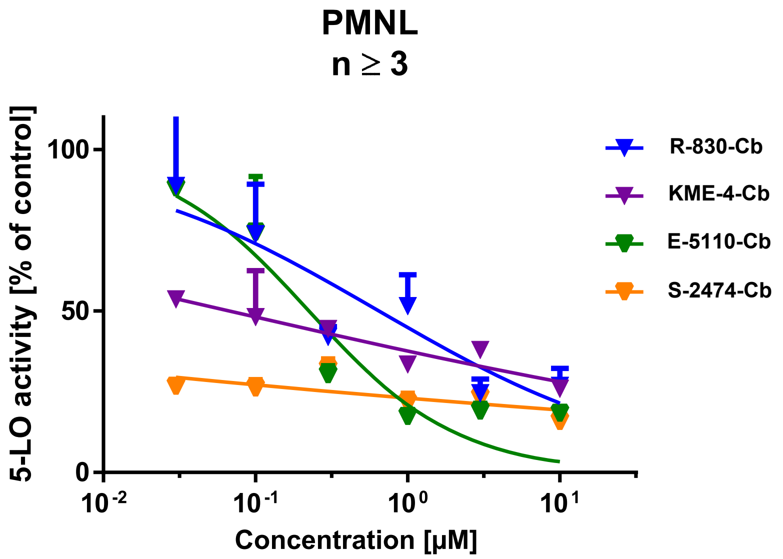

Figure S25. Dose-response curves for calculation of IC_{50} values for inhibition of 5-LO product formation in intact PMNL for the carborane derivatives **R-830-Cb**, **KME-4-Cb**, **E-5110-Cb**, and **S-2474-Cb**.

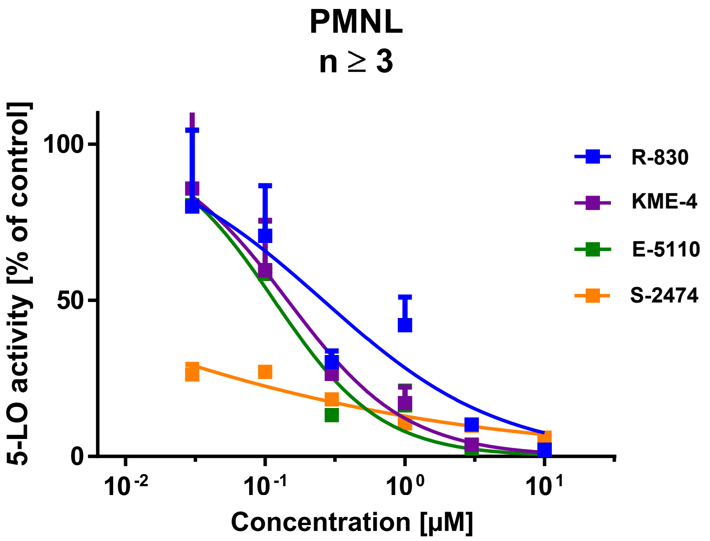

Figure S26. Dose-response curves for calculation of IC_{50} values for inhibition of 5-LO product formation in intact PMNL for the reference compounds **R-830**, **KME-4**, **E-5110**, and **S-2474**. Data are presented as mean of at least three independent experiments.

6.2. Cell viability studies on cancer cell lines and elucidation of the related mechanism

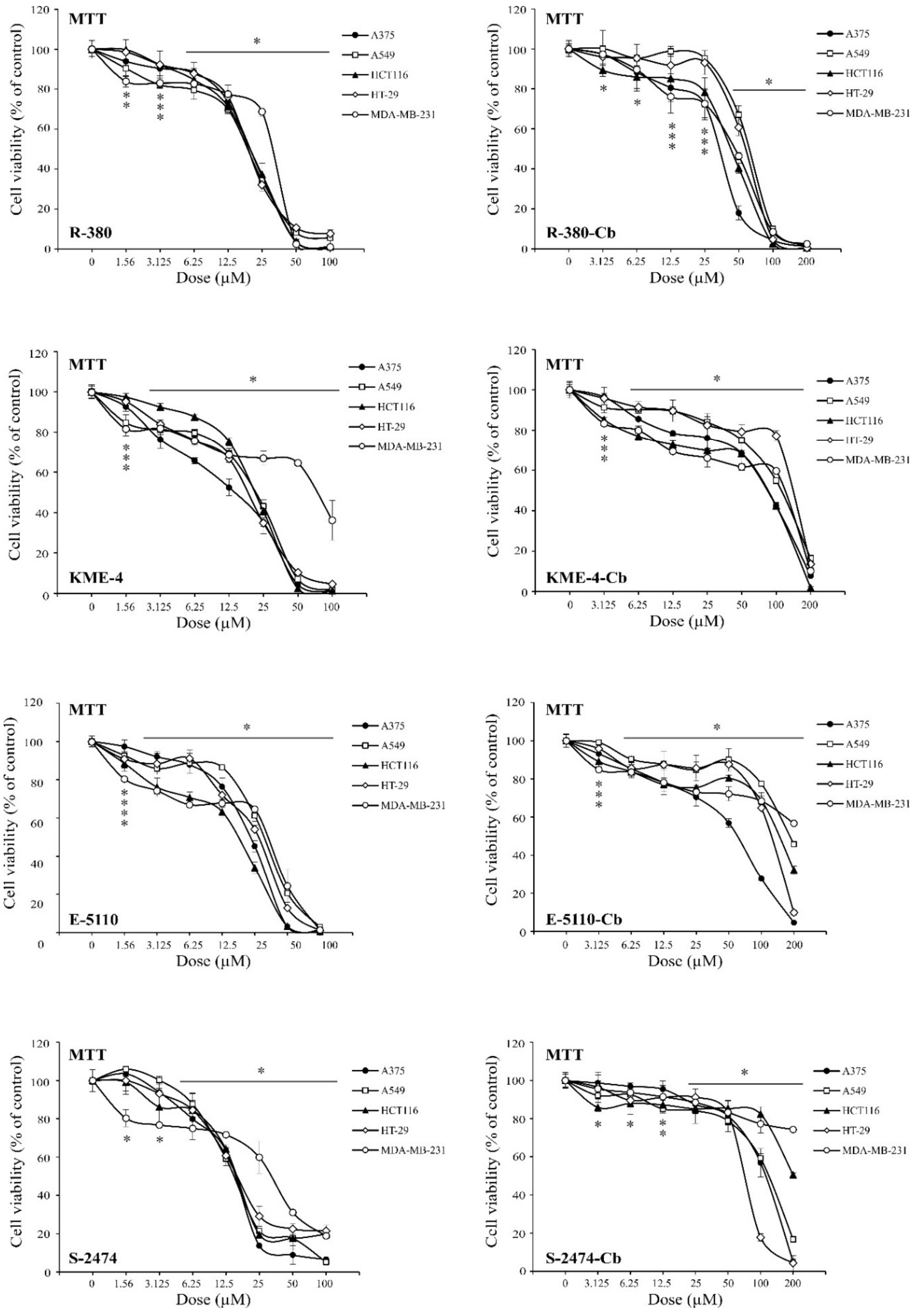

Figure S27. Dual inhibitors and carborane derivatives reduced the viability of cancer cells in a dose-dependent manner. Cells were treated with experimental compounds in concentrations ranging from 0 to 100 or 200 µM for 72 h. Cell viability was determined by the MTT test (MTT = 3-(4,5-dimethylthiazol-2-yl)-2,5-diphenyltetrazolium bromide). The data are presented as a mean ± SD from one representative out of three inde-pendent experiments. *p<0.05 refer to untreated cultures.

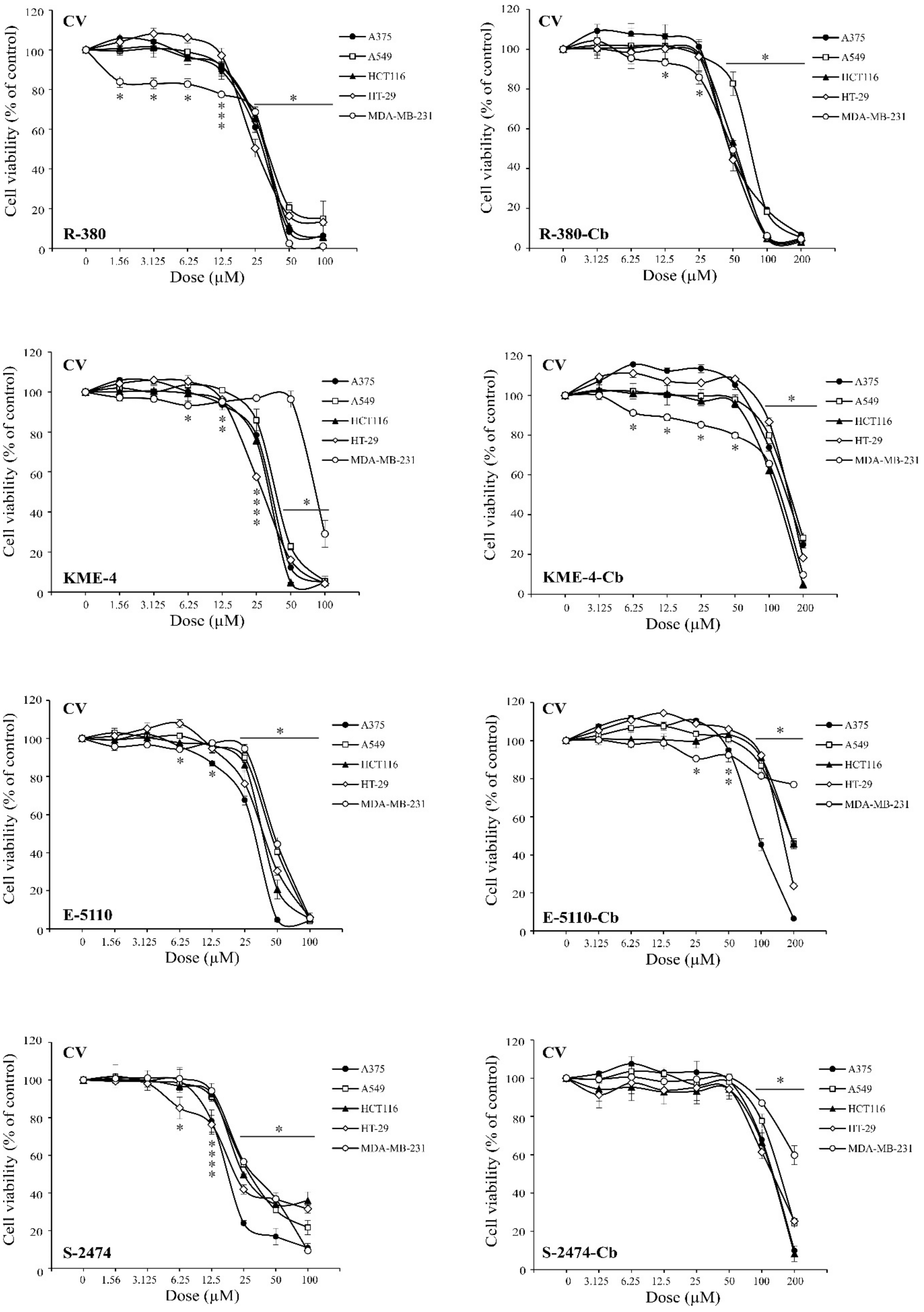

Figure S28. Dual inhibitors and carborane derivatives reduced the viability of cancer cells in a dose-dependent manner. Cells were treated with experimental compounds in concentrations ranging from 0 to 100 or 200 μM for 72 h. Cell viability was determined by the CV (CV = crystal violet) test. The data are presented as a mean ± SD from one representative out of three independent experiments. *$p<0.05$ refer to untreated cultures.

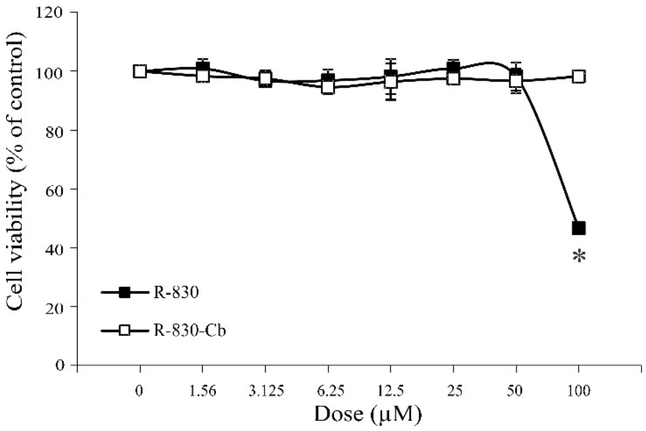

Figure S29. The influence of **R-830** and **R-830-Cb** on the viability of peritoneal exudate cells. Cells were treated with **R-830** and **R-830-Cb** in the indicated concentrations for 72 h. Cell viability was determined by the CV test. The data are presented as a mean ± SD from one representative out of three independent experiments. *$p<0.05$ refer to untreated cultures.

7. Single Crystal X-ray diffraction data

The data were collected on a Gemini diffractometer (Rigaku Oxford Diffraction) using Mo-Kα radiation and ω-scan rotation. Data reduction was performed with CrysAlisPro [1] including the program SCALE3 ABSPACK [2] for empirical absorption correction. All structures were solved by dual space methods with SHELXT [3] and the refinement was performed with SHELXL [4]. Structure figures were generated with DIAMOND-4 [5]

CCDC deposition numbers given in Table 7.1 to 7.8 contains the supplementary crystallographic data for this paper. These data can be obtained free of charge via https://summary.ccdc.cam.ac.uk/structure-summary-form (or from the Cambridge Crystallographic Data Centre, 12 Union Road, Cambridge CB2 1EZ, UK; fax: (+44)1223-336-033; or deposit@ccdc.cam.ac.uk).

7.1. 1-(*tert*-Butyl-dimethylsiloxy-12-(thiophen-2'-carbonyl)-1,12-dicarba-*closo*-decaborane(12) (**1**)

Empirical formula	$C_{13}H_{28}B_{10}O_2SSi$	
Formula weight	384.60	
Temperature	130(2) K	
Wavelength	71.073 pm	
Crystal system	Monoclinic	
Space group	$P2_1/n$	
Unit cell dimensions	a = 677.29(1) pm	α = 90°
	b = 3031.31(8) pm	β = 101.577(2)°
	c = 1076.31(3) pm	γ = 90°
Volume	2.16479(9) nm^3	
Z	4	
Density (calculated)	1.180 Mg/m^3	
Absorption coefficient	0.211 mm^{-1}	
F(000)	808	
Crystal size	0.60 x 0.10 x 0.04 mm^3	
Θ range for data collection	2.045 to 28.333°	
Index ranges	$-8 \leq h \leq 8$, $-39 \leq k \leq 39$, $-14 \leq l \leq 14$	
Reflections collected	19459	
Independent reflections	4968 [R(int) = 0.0406]	
Completeness to Θ = 26.375°	100.0 %	
Absorption correction	Semi-empirical from equivalents	
Max. and min. transmission	1.00000 and 0.96375	
Refinement method	Full-matrix least-squares on F^2	
Data / restraints / parameters	4968 / 0 / 356	
Goodness-of-fit on F^2	1.081	
Final R indices [I>2σ(I)]	R1 = 0.0486, wR2 = 0.0977	
R indices (all data)	R1 = 0.0667, wR2 = 0.1045	
Largest diff. peak and hole	0.316 and −0.246 e·Å^{-3}	
CCDC deposition number	2262623	

Comments: All H atoms were located on difference Fourier maps calculated at the final stage of the structure refinement. Carborane carbon atoms localized with bond length and displacement parameter analysis.

7.2. 1-Hydroxy-12-(thiophen-2’-carbonyl)-1,12-dicarba-*closo*-decaborane(12) (**R-830-Cb**)

Empirical formula	$C_7H_{14}B_{10}O_2S$	
Formula weight	270.34	
Temperature	130(2) K	
Wavelength	71.073 pm	
Crystal system	Monoclinic	
Space group	$P2_1/c$	
Unit cell dimensions	a = 1350.13(5) pm	α = 90°
	b = 1504.54(6) pm	β = 106.624(4)°
	c = 1404.56(5) pm	γ = 90°
Volume	2.7339(2) nm^3	
Z	8	
Density (calculated)	1.314 Mg/m^3	
Absorption coefficient	0.220 mm^{-1}	
F(000)	1104	
Crystal size	0.50 x 0.05 x 0.01 mm^3	
Θ range for data collection	2.030 to 28.999°	
Index ranges	−18 ≤ h ≤ 17, −19 ≤ k ≤ 20, −19 ≤ l ≤ 18	
Reflections collected	26151	
Independent reflections	6558 [R(int) = 0.0720]	
Completeness to Θ = 26.375°	100.0 %	
Absorption correction	Semi-empirical from equivalents	
Max. and min. transmission	1.00000 and 0.99179	
Refinement method	Full-matrix least-squares on F^2	
Data / restraints / parameters	6558 / 0 / 475	
Goodness-of-fit on F^2	1.016	
Final R indices [I>2σ(I)]	R1 = 0.0575, wR2 = 0.1058	
R indices (all data)	R1 = 0.1137, wR2 = 0.1271	
Largest diff. peak and hole	0.352 and −0.306 e·$Å^{-3}$	
CCDC deposition number	2262624	

Comments: Anisotropic refinement of all non-hydrogen atoms, except disordered atoms S1F and S2F. Excluding H(5) and H(12) of the disordered region, all hydrogen atoms were located on difference Fourier maps calculated at the final stage of the structure refinement. All C_4H_3S-rings are marginally disordered. For these rings only the disordered S atom was used for the disorder calculation. S1 : S1F = 0.944(2) : 0.056(2); S2 : S2F = 0.934(2) : 0.066(2). With intermolecular OH···O hydrogen donor acceptor bonds chains along (010) are formed.

Table S3. Selected bond lengths [pm] and bond angles [°] of compound **R-830-Cb**.

Bond lengths [pm]		Bond angles [°]		Bond lengths [pm]		Bond angles [°]	
S(1)-C(4)	171.7(2)	C(3)-C(4)-S(1)	114.6(3)	S(2)-C(11)	171.2(3)	C(10)-C(11)-S(2)	115.9(2)
C(3)-C(4)	145.2(3)	O(2)-C(3)-C(4)	119.5(2)	C(10)-C(11)	145.1(3)	O(4)-C(10)-C(11)	118.6(2)
O(2)-C(3)	121.8(3)	C(4)-C(3)-C(2)	123.0(2)	O(4)-C(10)	122.0(3)	C(11)-C(10)-C(9)	122.8(2)
C(2)-C(3)	152.7(3)	O(2)-C(3)-C(2)	117.6(2)	C(9)-C(10)	151.8(3)	O(4)-C(10)-C(9)	118.5(2)
C(2)-B(8)	171.1(4)	C(3)-C(2)-B(8)	121.4(2)	C(9)-B(19)	172.4(3)	C(10)-C(9)-B(19)	119.4(2)
B(2)-B(8)	176.0(4)	C(2)-B(8)-B(2)	105.0(2)	B(13)-B(19)	175.1(4)	C(9)-B(19)-B(13)	104.3(2)
C(1)-B(2)	170.0(4)	C(1)-B(2)-B(8)	104.7(7)	C(8)-B(13)	169.4(3)	C(8)-B(13)-B(19)	105.2(2)
O(1)-C(1)	138.3(3)	O(1)-C(1)-B(2)	115.4(2)	O(3)-C(8)	138.3(3)	O(3)-C(8)-B(13)	113.8(2)

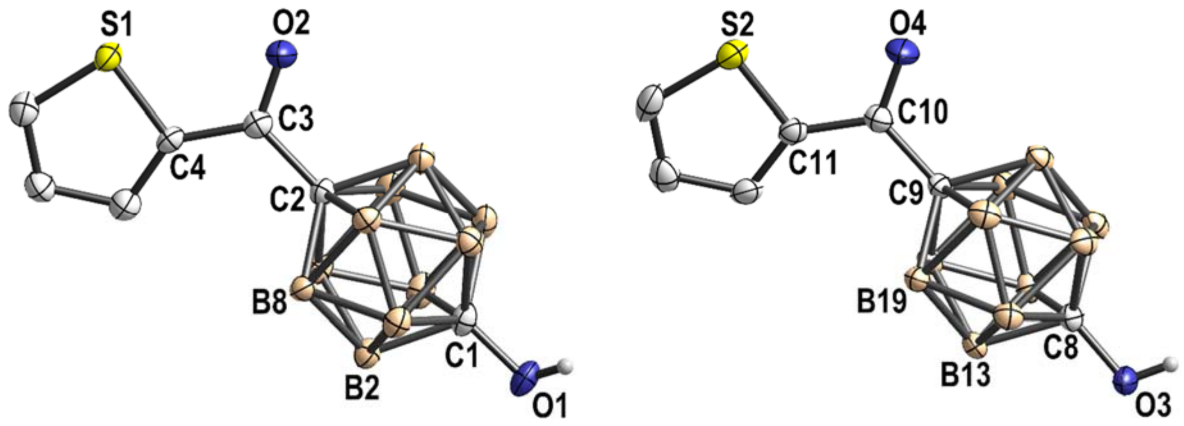

Figure S30. Asymmetric unit of (**R-830-Cb**). Hydrogen atoms, except OH, are omitted for clarity. Thermal ellipsoids are drawn at the 50% probability level.

7.3. (3*E*)-1-(*tert*-Butyl-dimethylsiloxy)-12-(methylene-[2’-ethylisothiazolidine-1’,1’-dioxide])-1,12-di-carba-*closo*-decaborane(12) (**5**)

Empirical formula	$C_{14}H_{35}B_{10}NO_3SSi$	
Formula weight	433.68	
Temperature	130(2) K	
Wavelength	71.073 pm	
Crystal system	Orthorhombic	
Space group	$Pca2_1$	
Unit cell dimensions	a = 2004.11(3) pm	α = 90°
	b = 677.24(1) pm	β = 90°
	c = 3625.62(4) pm	γ = 90°
Volume	4.9209(1) nm^3	
Z	8	
Density (calculated)	1.171 Mg/m^3	
Absorption coefficient	0.196 mm^{-1}	
F(000)	1840	
Crystal size	0.50 x 0.36 x 0.10 mm^3	
Θ range for data collection	2.109 to 26.703°	
Index ranges	$-24 \leq h \leq 24$, $-8 \leq k \leq 7$, $-45 \leq l \leq 45$	
Reflections collected	33480	
Independent reflections	9441 [R(int) = 0.0333]	
Completeness to Θ = 25.350°	99.9 %	
Absorption correction	Semi-empirical from equivalents	
Max. and min. transmission	1.00000 and 0.99286	
Refinement method	Full-matrix least-squares on F^2	
Data / restraints / parameters	9441 / 1 / 634	
Goodness-of-fit on F^2	1.049	
Final R indices [I>2σ(I)]	R1 = 0.0394, wR2 = 0.0945	
R indices (all data)	R1 = 0.0454, wR2 = 0.0987	
Absolute structure parameter	0.20(9)	
Largest diff. peak and hole	0.759 and −0.292 $e{\cdot}Å^{-3}$	
CCDC deposition number	2262625	

Comments: All CH hydrogen atoms were calculated on idealized positions whereas all BH hydrogen atoms were located on difference Fourier maps calculated at the final stage of the structure refinement. Carborane carbon atoms localized with bond length and displacement parameter analysis. Refined as a racemic twin. Twin domain ratio: 0.80(9): 0.20(9).

7.4. (3*E*)-1-Hydroxy-12-(methylene-[2'-ethylisothiazolidine-1',1'-dioxide])-1,12-dicarba-*closo*-decaborane(12) (**S-2474-Cb**)

Empirical formula	$C_8H_{21}B_{10}NO_3S$	
Formula weight	319.42	
Temperature	130(2) K	
Wavelength	71.073 pm	
Crystal system	Monoclinic	
Space group	$P2_1/c$	
Unit cell dimensions	a = 703.72(1) pm	α = 90°
	b = 1391.56(2) pm	β = 98.845(2)°
	c = 1718.73(3) pm	γ = 90°
Volume	1.66308(5) nm^3	
Z	4	
Density (calculated)	1.276 Mg/m^3	
Absorption coefficient	0.197 mm^{-1}	
F(000)	664	
Crystal size	0.35 x 0.25 x 0.15 mm^3	
Θ range for data collection	2.399 to 32.603°	
Index ranges	$-10 \le h \le 10$, $-20 \le k \le 21$, $-25 \le l \le 25$	
Reflections collected	21270	
Independent reflections	5616 [R(int) = 0.0319]	
Completeness to Θ = 30.510°	100.0 %	
Absorption correction	Semi-empirical from equivalents	
Max. and min. transmission	1.00000 and 0.99708	
Refinement method	Full-matrix least-squares on F^2	
Data / restraints / parameters	5616 / 0 / 292	
Goodness-of-fit on F^2	1.056	
Final R indices [I>2σ(I)]	R1 = 0.0371, wR2 = 0.0838	
R indices (all data)	R1 = 0.0508, wR2 = 0.0901	
Largest diff. peak and hole	0.323 and −0.380 e·Å^{-3}	
CCDC deposition number	2262626	

Comments: All H atoms were located on difference Fourier maps calculated at the final stage of the structure refinement. Carborane carbon atoms localized with bond length and displacement parameter analysis. With intermolecular OH···O hydrogen donor acceptor bonds zig-zag chains along (010) are formed.

Table S4. Selected bond lengths [pm] and bond angles [°] of compound **S-2474-Cb**.

**Bond lengths [pm]**		**Bond angles [°]**	
S(1)-N(1)	163.6(3)	O(2)-S(1)-N(1)	109.7(4)
S(1)-O(2)	144.1(3)	O(2)-S(1)-C(4)	113.1(4)
S(1)-C(4)	176.5(1)	N(1)-S(1)-C(4)	93.5(7)
C(4)-C(3)	132.8(8)	C(3)-C(4)-S(1)	116.7(5)
C(2)-C(3)	149.1(3)	C(4)-C(3)-C(2)	127.5(5)
C(2)-B(9)	171.7(3)	C(3)-C(2)-B(9)	121.8(1)
B(4)-B(9)	175.4(3)	C(2)-B(9)-B(4)	104.9(9)
C(1)-B(4)	171.8(6)	C(1)-B(4)-B(9)	104.6(2)
O(1)-C(1)	137.4(2)	O(1)-C(1)-B(4)	115.2(0)

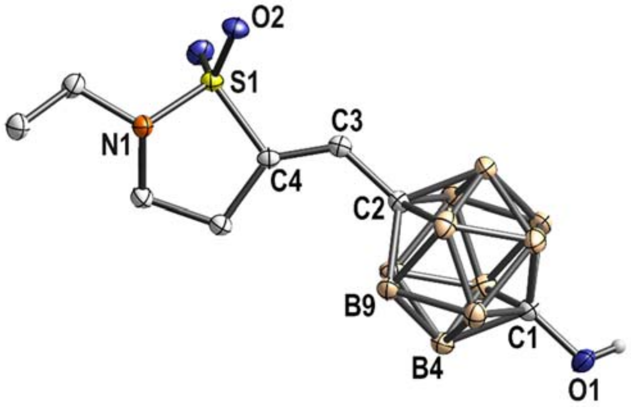

Figure S31. Asymmetric unit of **S-2474-Cb**. Hydrogen atoms, except OH, are omitted for clarity. Thermal ellipsoids are drawn at the 50% probability level.

7.5. (3*E*)-1-(*tert*-Butyl-dimethylsiloxy)-12-(methylene-[dihydrofurane-2'(3*H*)-one])-1,12-dicarba-*closo*-decaborane(12) (**6**)

Empirical formula	$C_{13}H_{30}B_{10}O_3Si$	
Formula weight	370.56	
Temperature	130(2) K	
Wavelength	71.073 pm	
Crystal system	Monoclinic	
Space group	$P2_1/c$	
Unit cell dimensions	a = 2321.09(8) pm	α = 90°
	b = 685.76(2) pm	β = 107.415(3)°
	c = 1398.88(4) pm	γ = 90°
Volume	2.1246(1) nm^3	
Z	4	
Density (calculated)	1.159 Mg/m^3	
Absorption coefficient	0.121 mm^{-1}	
F(000)	784	
Crystal size	0.40 x 0.30 x 0.03 mm^3	
Θ range for data collection	2.759 to 29.287°	
Index ranges	$-31 \leq h \leq 31$, $-9 \leq k \leq 8$, $-19 \leq l \leq 18$	
Reflections collected	25724	
Independent reflections	5190 [R(int) = 0.0528]	
Completeness to Θ = 26.375°	100.0 %	
Absorption correction	Semi-empirical from equivalents	
Max. and min. transmission	1.00000 and 0.84703	
Refinement method	Full-matrix least-squares on F^2	
Data / restraints / parameters	5190 / 136 / 423	
Goodness-of-fit on F^2	1.052	
Final R indices [I>2σ(I)]	R1 = 0.0539, wR2 = 0.1204	
R indices (all data)	R1 = 0.0782, wR2 = 0.1335	
Largest diff. peak and hole	0.361 and −0.307 e·Å^{-3}	
CCDC deposition number	2262627	

Comments: Excluding disordered regions, all H atoms were located on difference Fourier maps calculated at the final stage of the structure refinement. Marginally disordered molecule with a ratio of 0.933(2) : 0.067(2). Carborane carbon atoms localized with bond length and displacement parameter analysis.

7.6. (3*E*)-1-Hydroxy-12-(methylene-[dihydrofurane-2'(3*H*)-one])-1,12-dicarba-*closo*-decaborane(12) (**KME-4-Cb**)

Empirical formula	$C_7H_{16}B_{10}O_3$	
Formula weight	256.30	
Temperature	130(2) K	
Wavelength	71.073 pm	
Crystal system	Monoclinic	
Space group	$P2_1/n$	
Unit cell dimensions	a = 969.03(1) pm	α = 90°
	b = 1358.84(2) pm	β = 95.499(1)°
	c = 1025.96(1) pm	γ = 90°
Volume	1.34472(3) nm^3	
Z	4	
Density (calculated)	1.266 Mg/m^3	
Absorption coefficient	0.076 mm^{-1}	
F(000)	528	
Crystal size	0.35 x 0.30 x 0.25 mm^3	
Θ range for data collection	2.495 to 34.789°	
Index ranges	$-15 \le h \le 15$, $-21 \le k \le 21$, $-16 \le l \le 16$	
Reflections collected	36371	
Independent reflections	5583 [R(int) = 0.0381]	
Completeness to Θ = 33.140°	100.0 %	
Absorption correction	Semi-empirical from equivalents	
Max. and min. transmission	1.00000 and 0.95485	
Refinement method	Full-matrix least-squares on F^2	
Data / restraints / parameters	5583 / 0 / 245	
Goodness-of-fit on F^2	1.022	
Final R indices [I>2σ(I)]	R1 = 0.0440, wR2 = 0.1137	
R indices (all data)	R1 = 0.0581, wR2 = 0.1214	
Largest diff. peak and hole	0.422 and −0.202 e·Å^{-3}	
CCDC deposition number	2262628	

Comments: All H atoms were located on difference Fourier maps calculated at the final stage of the structure refinement. Carborane carbon atoms localized with bond length and displacement parameter analysis. With intermolecular OH···O donor acceptor bonds zig-zag chains along (010) are formed.

Table S5. Selected bond lengths [pm] and bond angles [°] of compound **KME-4-Cb**.

**Bond lengths [pm]**		**Bond angles [°]**	
O(2)-C(7)	133.4(0)	O(3)-C(7)-O(2)	120.5(2)
C(7)-O(3)	121.1(5)	O(2)-C(7)-C(4)	110.1(4)
C(7)-C(4)	148.7(0)	O(3)-C(7)-C(4)	129.3(4)
C(4)-C(3)	133.2(4)	C(3)-C(4)-C(7)	119.0(6)
C(2)-C(3)	148.9(6)	C(4)-C(3)-C(2)	128.7(8)
C(2)-B(10)	171.6(3)	C(3)-C(2)-B(10)	122.8(4)
B(5)-B(10)	175.8(9)	C(2)-B(10)-B(5)	104.7(1)
C(1)-B(5)	169.7(8)	C(1)-B(5)-B(10)	105.1(5)
O(1)-C(1)	137.3(2)	O(1)-C(1)-B(5)	115.1(4)

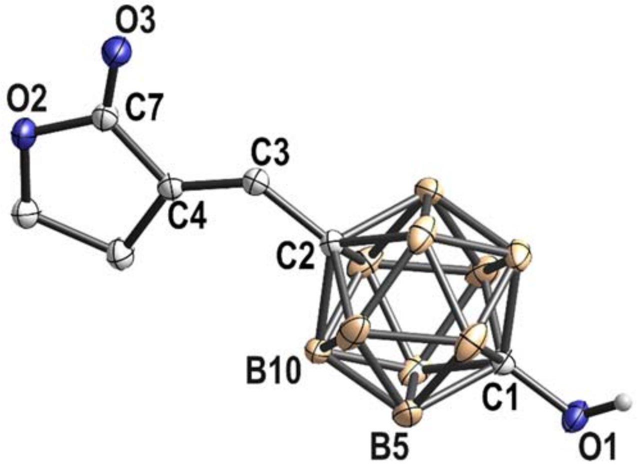

Figure S32. Asymmetric unit of **KME-4-Cb**. Hydrogen atoms, except OH, are omitted for clarity. Thermal ellipsoids are drawn at the 50 % probability level.

7.7. (3*E*)-1-(*tert*-Butyl-dimethylsiloxy)-12-(methylene-[1'-methoxypyrrolidine-2'-one])-1,12-dicarba-*closo*-decaborane(12) (**7**)

Empirical formula	$C_{14}H_{33}B_{10}NO_3Si$	
Formula weight	399.60	
Temperature	130(2) K	
Wavelength	71.073 pm	
Crystal system	Monoclinic	
Space group	$P2_1/n$	
Unit cell dimensions	a = 670.44(1) pm	α = 90°
	b = 2905.91(4) pm	β = 95.204(1)°
	c = 1181.73(1) pm	γ = 90°
Volume	2.29280(5) nm^3	
Z	4	
Density (calculated)	1.158 Mg/m^3	
Absorption coefficient	0.118 mm^{-1}	
F(000)	848	
Crystal size	0.37 x 0.28 x 0.24 mm^3	
Θ range for data collection	2.227 to 32.344°	
Index ranges	$-9 \le h \le 9$, $-42 \le k \le 42$, $-17 \le l \le 16$	
Reflections collected	39169	
Independent reflections	7710 [R(int) = 0.0293]	
Completeness to Θ = 30.510°	100.0 %	
Absorption correction	Semi-empirical from equivalents	
Max. and min. transmission	1.00000 and 0.99262	
Refinement method	Full-matrix least-squares on F^2	
Data / restraints / parameters	7710 / 76 / 440	
Goodness-of-fit on F^2	1.046	
Final R indices [I>2σ(I)]	R1 = 0.0501, wR2 = 0.1202	
R indices (all data)	R1 = 0.0658, wR2 = 0.1297	
Largest diff. peak and hole	0.398 and −0.351 $e\cdot Å^{-3}$	
CCDC deposition number	2262629	

Comments: Except disordered regions all H atoms were located on difference Fourier maps calculated at the final stage of the structure refinement. Disordered carborane substituent on two positions with a ratio of 0.846(2) : 0.154(2). Carborane carbon atoms localized with bond length and displacement parameter analysis.

7.8. (3*E*)-1-Hydroxy-12-(methylene-[1'-methoxypyrrolidine-2'-one])-1,12-dicarba-*closo*-decaborane(12) (**E-5110-Cb**)

Empirical formula	$C_8H_{19}B_{10}NO_3$	
Formula weight	285.34	
Temperature	130(2) K	
Wavelength	71.073 pm	
Crystal system	Monoclinic	
Space group	$P2_1/c$	
Unit cell dimensions	a = 746.89(1) pm	α = 90°
	b = 1894.94(4) pm	β = 94.865(2)°
	c = 1069.61(2) pm	γ = 90°
Volume	1.50838(5) nm^3	
Z	4	
Density (calculated)	1.257 Mg/m^3	
Absorption coefficient	0.076 mm^{-1}	
F(000)	592	
Crystal size	0.30 x 0.15 x 0.10 mm^3	
Θ range for data collection	2.149 to 30.228°	
Index ranges	$-10 \le h \le 10$, $-26 \le k \le 26$, $-14 \le l \le 13$	
Reflections collected	16112	
Independent reflections	4184 [R(int) = 0.0328]	
Completeness to Θ = 28.285°	100.0 %	
Absorption correction	Semi-empirical from equivalents	
Max. and min. transmission	1.00000 and 0.99504	
Refinement method	Full-matrix least-squares on F^2	
Data / restraints / parameters	4184 / 0 / 275	
Goodness-of-fit on F^2	1.020	
Final R indices [I>2σ(I)]	R1 = 0.0417, wR2 = 0.0999	
R indices (all data)	R1 = 0.0649, wR2 = 0.1118	
Largest diff. peak and hole	0.322 and −0.204 $e \cdot Å^{-3}$	
CCDC deposition number	2262630	

Comments: All H atoms were located on difference Fourier maps calculated at the final stage of the structure refinement. Carborane carbon atoms localized with bond length and displacement parameter analysis. With intermolecular hydrogen donor acceptor bonds chains along (001) are formed.

Table S6. Selected bond lengths [pm] and bond angles [°] of compound **E-5110-Cb**.

**Bond lengths [pm]**		**Bond angles [°]**	
O(3)-N(1)	138.1(9)	C(7)-N(1)-O(3)	120.9(3)
N(1)-C(7)	134.4(8)	O(2)-C(7)-N(1)	126.4(9)
O(2)-C(7)	122.9(3)	O(2)-C(7)-C(4)	127.8(4)
C(4)-C(7)	149.0(8)	N(1)-C(7)-C(4)	105.6(6)
C(3)-C(4)	132.8(4)	C(3)-C(4)-C(7)	118.3(2)
C(2)-C(3)	149.1(0)	C(4)-C(3)-C(2)	128.8(4)
C(2)-B(6)	171.2(9)	C(3)-C(2)-B(6)	121.1(7)
B(5)-B(6)	176.0(2)	C(2)-B(6)-B(5)	104.8(9)
C(1)-B(5)	171.8(8)	C(1)-B(5)-B(6)	105.1(5)
O(1)-C(1)	137.1(2)	O(1)-C(1)-B(5)	120.2(9)

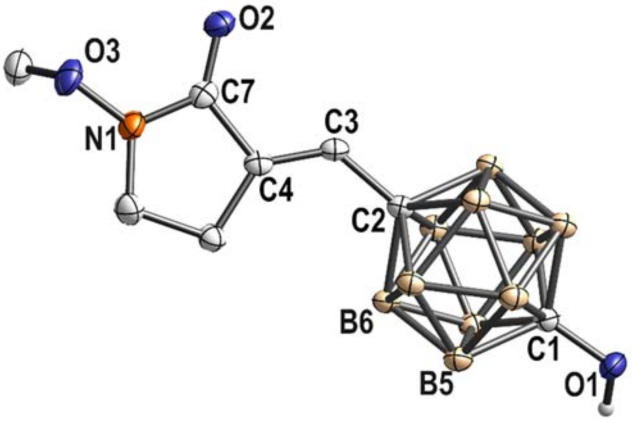

Figure S33. Asymmetric unit of **E-5110-Cb**. Hydrogen atoms, except OH, are omitted for clarity. Thermal ellipsoids are drawn at the 50 % probability level.

8. References

[1] Rigaku Oxford Diffraction. CrysAlisPro Software system, Rigaku Corporation, Wroclaw, Poland.

[2] SCALE3 ABSPACK. *Empirical absorption correction using spherical harmonics*; Oxford Diffraction: Abingdon, UK **2006**.

[3] G. M. Sheldrick. SHELXT – Integrated space-group and crystal-structure determination. *Acta Cryst A* **2015**, *71*, pp. 3–8.

[4] G. M. Sheldrick. Crystal structure refinement with SHELXL. *Acta Cryst C* **2015**, *71*, pp. 3–8.

[5] H. Putz, K. Brandenburg. *DIAMOND: Crystal and Molecular Structure Visualization*; Crystal Impact GbR: Bonn, Germany **2022**.
